# Transcriptome-wide profiling and quantification of *N*^6^-methyladenosine by enzyme-assisted adenosine deamination

**DOI:** 10.1038/s41587-022-01587-6

**Published:** 2023-01-02

**Authors:** Yu-Lan Xiao, Shun Liu, Ruiqi Ge, Yuan Wu, Chuan He, Mengjie Chen, Weixin Tang

**Affiliations:** 1 Department of Chemistry, The University of Chicago, Chicago, IL, USA.; 2 Institute for Biophysical Dynamics, The University of Chicago, Chicago, IL, USA.; 3 Department of Biochemistry and Molecular Biology, The University of Chicago, Chicago, IL, USA.; 4 Howard Hughes Medical Institute, The University of Chicago, Chicago, IL, USA.; 5 Department of Medicine, The University of Chicago, Chicago, IL, USA.; 6 Department of Human Genetics, The University of Chicago, Chicago, IL, USA.

## Abstract

*N*^6^-methyladenosine (m^6^A), the most abundant internal mRNA modification in higher eukaryotes, serves myriad roles in regulating cellular processes. Functional dissection of m^6^A, however, is hampered in part by the lack of high-resolution and quantitative detection methods. Here, we present evolved TadA-assisted *N*^6^-methyladenosine sequencing (eTAM-seq), an enzyme-assisted sequencing technology that detects and quantifies m^6^A by global adenosine deamination. With eTAM-seq, we analyze the transcriptome-wide distribution of m^6^A in HeLa and mouse embryonic stem cells. The enzymatic deamination route employed by eTAM-seq preserves RNA integrity, facilitating m^6^A detection from limited input samples. In addition to transcriptome-wide m^6^A profiling, we demonstrate site-specific, deep sequencing-free m^6^A quantification with as few as 10 cells, an input demand orders of magnitude lower than existing quantitative profiling methods. eTAM-seq will enable researchers to not only survey the m^6^A landscape at unprecedented resolution, but also detect m^6^A at user-specified loci with a simple workflow.

*N*^6^-methyladenosine (m^6^A), the most prevalent internal mRNA modification in higher eukaryotes, constitutes a regulatory network extensively involved in physiological and pathological processes^1–4^. m^6^A alters mRNA processing, structure, translation, and decay without changing the genetic code^5^. The regulatory mechanisms governed by m^6^A are highly heterogeneous; functional outcomes of m^6^A modifications vary significantly across different transcripts, different regions in the same transcript, and different cell types^5^. Comprehensive and quantitative mapping of m^6^A, aimed at elucidating the multitude of roles served by the modification, remains challenging, especially with limited input materials.

m^6^A-seq^6^ and MeRIP-seq^7^, the most widely used m^6^A-mapping methods, capture m^6^A-containing transcripts by antibody-mediated immunoprecipitation and detect m^6^A at a resolution of 100–200 nucleotides (nt). The enrichment process requires bulk input materials: a typical m^6^A-seq or MeRIP-seq workflow starts with mRNA extraction from millions of cells, precluding their application to samples of limited quantities. Although additional solutions have been proposed towards transcriptome-wide m^6^A profiling, including miCLIP^8^, MAZTER-seq^9^, m^6^A-REF-seq^10^, m^6^A-SEAL^11^, m^6^A-label-seq^12^, DART-seq^13^, and m^6^A-SAC-seq^14^, their applications are limited by input amounts, antibody specificities, crosslinking efficiencies, non-quantitative readouts, predefined or biased sequence contexts, overexpression of effector proteins, or complicated workflows.

Furthermore, the m^6^A field lacks a simple and cost-efficient method that quantifies the modification level at individual m^6^A sites to connect the methylation density, on for example pluripotency transcription factors in stem cells, with transcript abundances or development stage. Existing methods rely on oligonucleotide probes, which anneal to individual transcripts to enable m^6^A-dependent biochemical readout^11, 15, 16^. However, these methods demand large input materials (micrograms of total RNA, or millions of cells) and the input requirement scales with the number of m^6^A sites subject to evaluation. As a result, site-specific m^6^A quantification has so far only been demonstrated for abundant RNA species in cultured cell lines. The probe annealing process also faces specificity challenges, especially when targeting transcripts of low abundance, leading to inaccurate quantification. A method that allows facile detection of individual m^6^A sites with stoichiometry information would bridge this critical gap in epitranscriptomic research.

Here we report evolved TadA-assisted *N*^6^-methyladenosine sequencing (eTAM-seq), an enzyme-assisted sequencing technology for quantitative, base-resolution profiling of m^6^A. eTAM-seq functions by global adenosine deamination, enabling detection of m^6^A as persistent A. We demonstrate adenosine-to-inosine (I) conversion rates up to 99% using a hyperactive TadA variant. With eTAM-seq, we quantify m^6^A in the whole transcriptomes of HeLa and mouse embryonic stem cells (mESCs). Further, we showcase deep sequencing-free, site-specific m^6^A quantification with as few as 10 cells, an input demand orders of magnitude lower than existing quantitative profiling methods. Collectively, eTAM-seq enables faithful detection and quantification of m^6^A with limited RNA input, launching a robust solution to deciphering the epitranscriptome.

## Results

### Evolved TadA-assisted *N*^6^-methyladenosine sequencing of RNA

**Figure 1 |:**
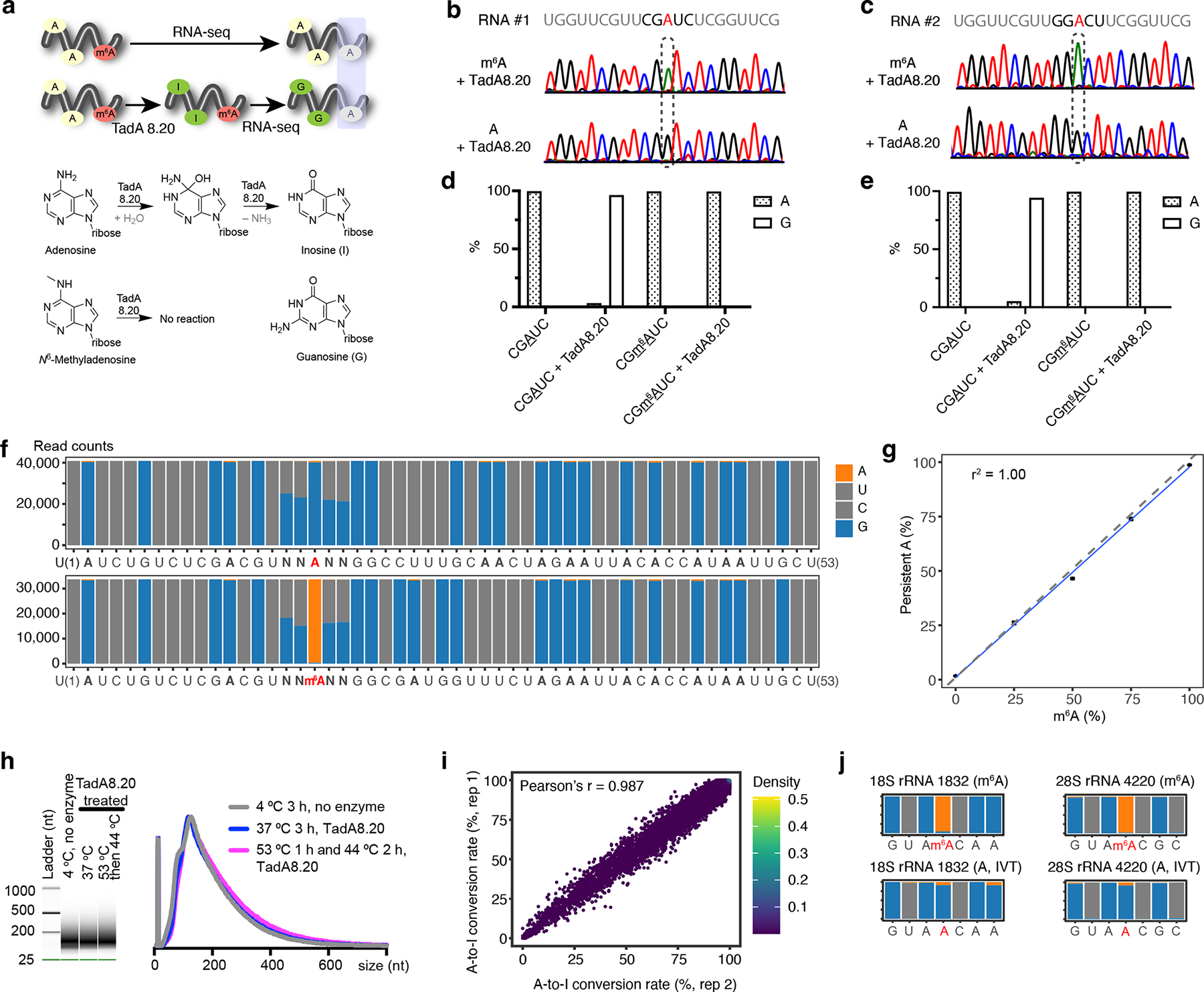
Global A deamination by TadA8.20. **a**. Proposed m^6^A detection scheme. TadA8.20 selectively converts A into I, without acting on m^6^A. I is recognized as G by reverse transcriptases. Persistent A post-TadA8.20 treatment corresponds to m^6^A. **b**, **c**. *In vitro* deamination of RNA probes hosting A or m^6^A in “CGAUC” (**b**) and “GGACU” (**c**) motifs by TadA8.20. Unmethylated and methylated RNA sequences were prepared through *in vitro* transcription using ATP and *N*^6^-methyl-ATP as starting materials, respectively. Treated RNA was reverse transcribed, amplified, and subjected to Sanger sequencing. **d**, **e**. TadA8.20-catalyzed A-to-I conversion rates in “CGAUC” (**d**) and “GGACU” (**e**) probes quantified by next-generation sequencing. **f**. Deamination of synthetic A/m^6^A RNA probes by TadA8.20. 53-nt RNA probes hosting NNANN and NNm^6^ANN motifs were treated by TadA-8.20. Deaminated RNA underwent RT and next-generation sequencing. **g**. Correlation of persistent A signals captured by eTAM-seq and m^6^A contents in RNA probes. **h**. Capillary gel electrophoresis analysis of fragmented HeLa mRNA treated with or without TadA8.20 at different temperatures for 3 h. RNA size distribution is plotted on the right. For eTAM-seq, RNA is incubated with TadA8.20 at 53°C for 1 h followed by 2 h treatment at 44°C. Experiments were repeated independently with similar results. **i**. Transcriptome-wide A-to-I conversion rates in two independent replicates. 10% of A sites with ≥100 counts were randomly sampled to make the scatter plot. Pearson’s r was calculated for all A sites with ≥100 counts. **j**. Two m^6^A sites in human rRNA. Positions 1829–1835 of 18S rRNA and positions 4217–4223 of 28S rRNA are plotted.

Our sequencing platform is inspired by the concept of bisulfite sequencing for methylation detection in DNA^17^, in which all unmethylated C is converted into U without impacting 5-methylcytosine. We envision global deamination of A but not m^6^A (Fig. 1a)—all unmethylated A is converted into I; I base-pairs with C and is read as G by reverse transcriptases. Persistent A corresponds to m^6^A.

Our search for global A deamination routes focused on enzymatic approaches that support high efficiency and mild reaction conditions. We hypothesized that laboratory-evolved hyperactive (deoxy)adenosine deaminases, unlike naturally occurring enzymes whose activity is tamed^18^, may facilitate robust global A deamination. Screening a panel of enzymes, we determined that TadA8.20^19–21^ (Supplementary Fig. 1a), an *E. coli* tRNA adenosine deaminase (TadA) variant evolved to function robustly on DNA with minimal context dependence, fit our criteria: when we placed a single A or m^6^A in different sequence contexts, TadA8.20 deaminated A close to completion without acting on m^6^A (Fig. 1b–1e and Supplementary Fig. 1b). TadA8.20 preserved RNA integrity, as RNA pre- and post-enzymatic treatment produced the same amount of complementary DNA (cDNA) during reverse transcription (Supplementary Fig. 1c).

Deamination efficiency is critical for faithful detection of m^6^A. We screened a series of assay conditions and found that temperature elevation from 37°C to 44°C or 53°C markedly improved A-to-I conversion rates, especially in regions resistant to deamination at 37°C (Supplementary Note 1 and Supplementary Fig. 2). We also showed that TadA8.20 purified in different batches functioned consistently in mediating A-to-I conversion (Supplementary Fig. 3).

We incorporated TadA8.20 into an RNA-seq workflow and developed evolved TadA-assisted *N*^6^-methyladenosine sequencing (eTAM-seq). We first assessed the efficiency and context dependence of the enzyme using synthetic RNA probes with A/m^6^A flanked by two Ns (N = A, C, G, or U). TadA8.20 attained a global A-to-I conversion rate of 99%, close to the efficiency offered by bisulfite treatment of C in RNA^22^, and rejected m^6^A completely (Fig. 1f). Importantly, TadA8.20 efficiently deaminates A in all DRACH sequences (D = A, G, or U; R = A or G; H = A, C, or U), the consensus motif hosting m^6^A modifications in eukaryotes (Supplementary Table 1).

We next investigated whether global A deamination enforced by TadA8.20 enables quantitative detection of m^6^A. We synthesized additional RNA probes containing 25%, 50%, and 75% m^6^A at the N flanked position (NNA/m^6^ANN) and labeled them with unique molecular identifiers (UMIs). After incubation with TadA8.20, we detected 1.69 ± 0.03%, 26.1 ± 0.6%, 46.5 ± 0.3%, 73.9 ± 0.5%, and 98.7 ± 0.1% A in probes hosting 0%, 25%, 50%, 75%, and 100% m^6^A, respectively (Fig. 1g and Supplementary Fig. 4). Since the persistent A ratio correlates linearly with the extent of m^6^A in synthetic RNA probes (r^2^ = 1.00, Fig. 1g), we conclude that eTAM-seq quantifies m^6^A at the site of interest. Collectively, we demonstrate that TadA8.20 is mild, robust, selective, and insensitive to sequence contexts, paving the road for quantitative and base-resolution detection of m^6^A in biological samples.

### eTAM-seq enables base-resolution detection of m^6^A in RNA

We applied eTAM-seq to 50 ng of mRNA extracted from HeLa cells. To reduce secondary structures and to facilitate downstream sequencing, we processed HeLa mRNA into ~150 nt fragments prior to incubation with TadA8.20. Capillary gel electrophoresis of RNA incubated with TadA8.20 for 3 h indicated that the size distribution of RNA remained unchanged with no noticeable sample loss (Fig. 1h). Fragmented mRNA treated with or without Tad8.20 behave similarly during library construction and RNA-seq, suggesting that the increased GC content^23^, a consequence of global A deamination, poses minimal impact on cDNA synthesis, amplification, and sequencing.

Following adapter removal, reads were mapped to the transcriptome allowing both A and G matched to genomic A sites. Given the reduced complexity of the transcriptome post-TadA8.20 treatment, we took conservative measures to ensure mapping accuracy: we only accepted reads ≥ 40 nt and discarded those that could be mapped to more than one genomic locus (Supplementary Table 2). Importantly, mRNA abundances reported by eTAM-seq are consistent with a published RNA-seq dataset^24^ (Pearson’s r = 0.84–0.85, Supplementary Fig. 5a), indicating that eTAM-seq sustains gene expression information captured by canonical RNA-seq. The conversion rate of a given A in the HeLa transcriptome is highly reproducible across biological replicates (Pearson’s r = 0.99, Fig. 1i, Supplementary Fig. 5b and 5c), suggesting that deamination efficiency is governed by intrinsic properties of RNA rather than random factors introduced during sample preparation.

Local secondary structures may shield a subset of A bases from TadA8.20, resulting in false positive persistent A signals in eTAM-seq, similar to bisulfite treatment-resistant cytosines in double-stranded RNA^25^. We developed a statistical model to determine the extent to which a given A site is shielded from TadA8.20 using a modification-free HeLa transcriptome prepared via *in vitro* transcription (IVT; Supplementary Fig. 6a and Supplementary Note 2)^26^. Sequence context, and consequently secondary structures and accessibility to TadA8.20, are consistent in HeLa and IVT samples. Persistent A signals that arise to the same extent in HeLa and modification-free IVT samples likely represent partially shielded unmethylated A sites. In our statistical model, “0” indicates a site fully blocked to TadA8.20 and “1” indicates a site fully accessible. We apply the accessibility parameter to the apparent methylation levels detected by eTAM-seq and determine the true methylation levels (Supplementary Note 3). The accessibility adjustment sustains persistent A signals arising from m^6^A, but eliminates those caused by secondary structures, thereby improving the fidelity of eTAM-seq.

We first analyzed reads mapped to ribosomal RNA (rRNA) because human rRNA not only hosts two known m^6^A sites but is also more structured than mRNA^27^. We detected ~1% rRNA reads in HeLa mRNA (Supplementary Table 2), a level typical for RNA purified by enriching polyadenylated sequences^28^. We observed a lower global A-to-I conversion rate in rRNA (85%), consistent with our hypothesis that highly structured RNA is more resistant to deamination. Nevertheless, HeLa and IVT samples produced the same levels of persistent A signals at structured sites (Supplementary Fig. 6b), which are therefore recognized as less accessible unmethylated A sites. Two well-characterized m^6^A sites in human rRNA, at position 1832 in 18S rRNA^29, 30^ and position 4220 in 28S rRNA^31, 32^, are cleanly detected by eTAM-seq (Fig. 1j).

eTAM-seq detects A chemically modified at the *N*^6^ position. *N*^6^,*N*^6^-dimethyladenosine (m^6^_2_A)^33, 34^, similar to m^6^A, is resistant to TadA8.20-catalyzed deamination and two conserved m^6^_2_A sites in human 18S rRNA^35^, positions 1850 and 1851, were detected by eTAM-seq (Supplementary Fig. 6c). On the other hand, 2’-*O*-methyladenosine (Am) is sensitive to TadA8.20 and will not generate persistent A signals in eTAM-seq (Supplementary Fig. 6d). m^6^_2_A, however, is extremely rare in mRNA and unlikely to make a significant contribution to eTAM-seq signals. *N*^6^,2’-*O*-dimethyladenosine (m^6^Am), another A modification bearing a methyl group at the *N*^6^ position, is located at the first transcribed nucleotide adjacent to the cap of ~10% of all mammalian mRNA^36, 37^. The terminal location of m^6^Am may lower its sequencing coverage. Moreover, m^6^Am was quantified to be 0.027% of A in fragmented and ligated mRNA, 95% lower than that of m^6^A (0.55% of A, Supplementary Fig. 6e). Collectively, we conclude that eTAM-seq predominantly detects m^6^A in mammalian mRNA.

### m^6^A profiling and quantification in the HeLa transcriptome

**Figure 2 |:**
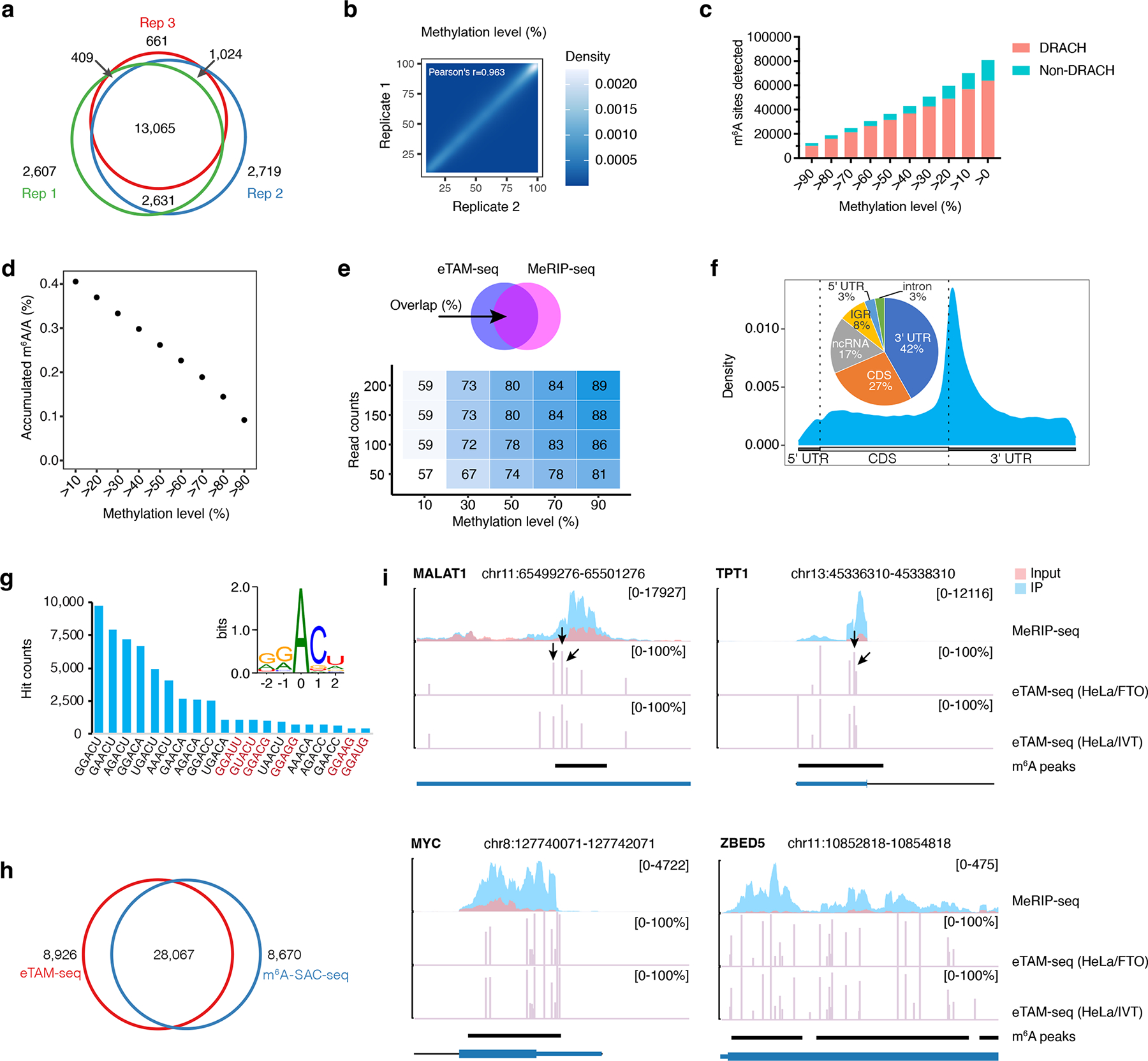
Transcriptome-wide m^6^A profiling in HeLa mRNA by eTAM-seq. **a**. Overlap analysis of m^6^A sites identified in three biological replicates of eTAM-seq (HeLa/IVT). **b**. Methylation levels of common sites detected in eTAM-seq (HeLa/IVT-1) and eTAM-seq (HeLa/IVT-2). Correlative analyses on methylation levels reported by replicate 1 vs 3 and 2 vs 3 are provided in Supplementary Fig. 7. **c**. Hit distributions in DRACH and non-DRACH sequences at different methylation levels. **d**. Cumulative m^6^A signals from highly methylated sites to lowly methylated sites (right to left). m^6^A constitutes 0.41% of all A subject to evaluation in the HeLa transcriptome. **e**. Overlap analysis of m^6^A sites identified by eTAM-seq and peak clusters generated via MeRIP-seq. The overlap between eTAM-seq and MeRIP-seq increases with higher read depth and methylation levels. **f**. Metagene plot of transcriptome-wide distribution of m^6^A. m^6^A distributions across different RNA regions are provided in the inserted pie chart. IGR: intergenic region; ncRNA: non-coding RNA. **g**. Major sequence motifs hosting m^6^A. DRACH motifs are in black and non-DRACH motifs are colored in red. The consensus sequence hosting m^6^A is inserted. **h**. m^6^A sites co-discovered by eTAM-seq and m^6^A-SAC-seq. Hits in DGACU captured by both methods are subject to overlap analysis. m^6^A-SAC-seq dataset: GSE198246^40^. **i**. m^6^A positions and fractions in MALAT1, TPT1, MYC, and ZBED5. eTAM-seq signals are plotted as methylation levels (%) alongside MeRIP-seq peaks in normalized read coverage. Note that eTAM-seq (HeLa/IVT) has slightly higher coverage than eTAM-seq (HeLa/FTO) and may therefore capture more m^6^A sites. MALAT1_2515, 2577, 2611 and TPT1_687, 703 are indicated by arrows. The coding sequence for TPT1 is on the minus strand of the genome.

We next profiled m^6^A in the HeLa transcriptome. To assess reproducibility of eTAM-seq, we carried out three biological replicates: replicate 1 (HeLa-1 and HeLa-IVT1), replicate 2 (HeLa-2 and HeLa-IVT2), and replicate 3 (HeLa-3 and HeLa-IVT3). We processed the three replicates separately and called out m^6^A sites with exposed methylation levels ≥ 10% (methylation level * site accessibility ≥ 10%, Supplementary Note 2). We chose this cutoff to remove sites of extremely low methylation and accessibility. We identified 18,712, 19,439, and 15,159 m^6^A sites from replicates 1, 2, and 3, respectively, 16,376 (88%), 16,600 (85%), and 13,151 (87%) of which were found in DRACH motifs (Fig. 2a and Supplementary Fig. 7). As DRACH motifs host ~70% of m^6^A sites^8^, and only ~7% of all A sites, in mammalian transcriptomes, the observed hit distribution among DRACH and non-DRACH sequences supports the robustness of eTAM-seq. Of the identified hits, only 2,607 (14%), 2,719 (14%), and 661 (4%) are unique to individual replicates (Fig. 2a). Sites common to the three replicates show highly consistent methylation levels (Pearson’s r = 0.96, Fig. 2b, Supplementary Fig. 7, and Supplementary Note 4), confirming the reproducibility of eTAM-seq.

Next, we sequenced replicate 1 deeper to better uncover the m^6^A landscape in HeLa cells. We detected 80,941 m^6^A sites in HeLa mRNA, of which 12,454 sites (15%) showed methylation levels greater than 90% (Supplementary 8a). Methylated sites are enriched in DRACH sequences (Fig. 2c), highlighting the strong motif preference of the m^6^A writer complex. m^6^A constitutes 0.41% of all A subject to evaluation (Fig. 2d). Given that the m^6^A fraction in the HeLa transcriptome was determined to be 0.2–0.6% by liquid chromatography/mass spectrometry^38^ (0.55% in this work, Supplementary Fig. 6e), we conclude that eTAM-seq captures the majority of m^6^A sites in the input RNA.

We extracted m^6^A sites with exposed methylation levels ≥ 10% (69,834) for further analysis. 34,049 (49%) of these m^6^A sites overlap with a published MeRIP-seq dataset^24^ and account for 8,398 (52%) of peaks detected by MeRIP-seq (Supplementary Fig. 8b). When we consider sites of ≥ 200 counts and ≥ 90% methylation, 89% of eTAM-seq hits are co-discovered by MeRIP-seq (Fig. 2e). One MeRIP-seq peak covers 4.1 m^6^A sites averagely (median: 3, Supplementary Fig. 8c). We note that these numbers may not directly translate to other studies as MeRIP-seq can be affected by antibody specificity, immunoprecipitation workflow, and sequencing depth^39^, whereas eTAM-seq may detect additional m^6^A sites if more genomic A sites are effectively sampled. m^6^A sites are enriched around stop codons with significant distribution across 5’ untranslated regions (UTRs), coding sequences (CDS), and 3’ UTRs (Fig. 2f), consistent with the transcriptome-wide m^6^A distribution reported by orthogonal detection methods^6, 7^. A clear DRACH motif emerges in sequences surrounding m^6^A (Fig. 2g). Further sequence context dissection of these m^6^A sites reveals 14.7% from GGACU, 11.8% from GAACU, and 10.5% from AGACU, a distribution fully corroborated by miCLIP^8^ (Fig. 2g and Supplementary Fig. 8d). We also overlapped eTAM-seq hits with m^6^A-SAC-seq^14, 40^, a recently developed base-resolution m^6^A detection method. As m^6^A-SAC-seq is more sensitive to GA sequences, we limited our comparison to DGACH hits. eTAM-seq detects 36,993 m^6^A sites in DGACH, 28,067 (76%) of which are co-discovered by m^6^A-SAC-seq (28,067/36,737, 76%, Fig. 2h and Supplementary Fig. 8e).

Of all sequencing-captured A sites, 92% show accessibility ≥ 0.9 (Supplementary Fig. 8f), indicating that the majority of the A sites in the HeLa transcriptome are sensitive to eTAM-seq. Accessibility of methylated A sites, including those methylated to >90%, is overwhelmingly skewed towards 1 (Supplementary Fig. 8g), *i.e.*, fully deaminated in the IVT control, indicating that persistent A signals observed at these sites in Tad8.20-treated mRNA are a result of methylation instead of incomplete deamination (Supplementary Fig. 9). We extract from the HeLa transcriptome sites subject to endogenous A-to-I editing, which are almost mutually exclusive from eTAM-seq hits (Supplementary Note 5 and Supplementary Fig. 10). Collectively, eTAM-seq is minimally impacted by endogenous RNA editing and is robust against false positive detection.

In addition to the IVT transcriptome, we employed an orthogonal control for eTAM-seq wherein the demethylase FTO was applied to HeLa mRNA to provide a demethylated transcriptome (Supplementary Fig. 11). FTO demethylated a large portion of m^6^A in spike-in RNA probes (Supplementary Fig. 12), in line with previous reports^26^. FTO treatment should only impact the deamination level of methylated A sites. Therefore, positions with significantly lower levels of persistent A following eTAM-seq in the FTO-treated sample (FTO+) compared with the original untreated sample (FTO−) were extracted as m^6^A sites (Supplementary Note 6). With this workflow, we identified 47,840 m^6^A sites, 40,096 (84%) of which show exposed methylation levels ≥ 10% (Supplementary Note 7, Supplementary Fig. 13 and 14). Most hits (95%) are co-discovered with the HeLa/IVT dataset (Supplementary Fig. 14c) and show highly consistent methylation levels (Pearson’s r = 0.996, Supplementary Fig. 14d). Of the 40,096 m^6^A sites, 60% (24,044) overlap with a published MeRIP-seq dataset^24^, and cover 50% of MeRIP-seq peaks (Supplementary Fig. 14e and 14f). Taken together, these results suggest eTAM-seq functions consistently in profiling m^6^A regardless of the mechanism through which the demethylated reference transcriptome (IVT or FTO demethylation) is prepared.

We next evaluated the quantitative feature of eTAM-seq using five representative sites—MALAT1_2515, 2577, 2611 and TPT1_687, 703, the methylation levels of which have been previously determined to be 61%, 80%, 38%, 15%, and 1% by site-specific cleavage and radioactive-labeling followed by ligation-assisted extraction and thin-layer chromatography (SCARLET)^15^. eTAM-seq reported m^6^A fractions of 58%, 81%, and 51% for MALAT1_2515, 2577, and 2611 (Fig. 2i), respectively, in line with SCARLET results. However, we observe much higher modification levels than previously reported for TPT1_687 and 703 – 42% and 77% (Fig. 2i). As results from the eTAM-seq (HeLa/IVT), eTAM-seq (HeLa/FTO), and m^6^A-SAC-seq datasets corroborate each other (Supplementary Fig. 15), we find these quantification results reliable. Discrepancies between our study and the SCARLET study may be explained by 1) differences in HeLa cells and 2) inefficient/off-target probe annealing in SCARLET.

Lastly, we extracted eTAM-seq signals from 18 additional loci and overlayed them with a published MeRIP-seq dataset^24^ (Fig. 2i and Supplementary Fig. 16). In all 20 cases, including MALAT1 and TPT1, eTAM-seq peaks are found in or close to MeRIP-seq peaks. Of the 20 transcripts inspected, 19 host multiple m^6^A sites with several transcripts heavily methylated, such as ZBED5, MYC, and CXCR4, which bear 31, 22, and 23 m^6^A sites, respectively. These results, taken together, confirm eTAM-seq is robust and reliable in capturing and quantifying m^6^A sites in the whole transcriptome.

### Mapping m^6^A in transcriptome of mouse embryonic stem cells

**Figure 3 |:**
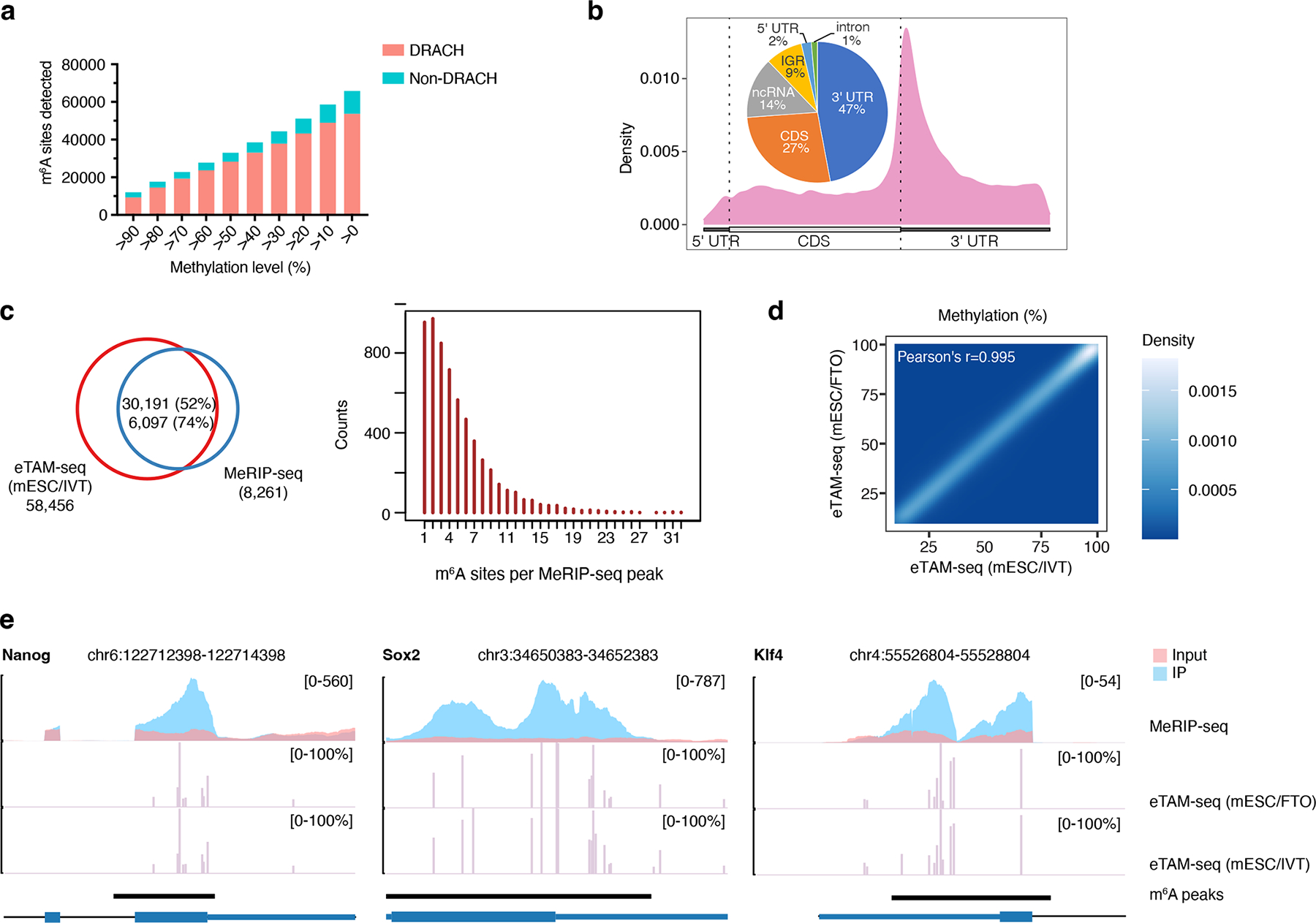
m^6^A profiling in mouse embryonic stem cells (mESCs) by eTAM-seq. **a**. Hit distributions in DRACH and non-DRACH sequences across different methylation levels. m^6^A sites identified by eTAM-seq (mESC/IVT) are plotted. **b**. Metagene plot of transcriptome-wide distribution of m^6^A. m^6^A distributions across different RNA regions are inserted. IGR: intergenic region; ncRNA: non-coding RNA. **c**. Overlap analysis of m^6^A sites identified by eTAM-seq and peak clusters generated via MeRIP-seq. Hits detected by eTAM-seq (mESC/IVT) are overlapped with a published MeRIP-seq dataset (left)^26^. One MeRIP-seq peak covers multiple m^6^A sites (right). Similar analyses using the eTAM-seq (mESC/FTO) dataset are provided in Supplementary Fig. 18. **d**. Methylation levels reported by eTAM-seq (mESC/IVT) and eTAM-seq (mESC/FTO). **e**. m^6^A positions and fractions in selected regions of Nanog, Sox2, and Klf4. eTAM-seq hits are plotted in methylation levels (%) and are juxtaposed with MeRIP-seq peaks in normalized read coverage. For a zoomed-out view of m^6^A distribution in full-length Nanog, Sox2, and Klf4, see Supplementary Fig. 19.

We next applied eTAM-seq to explore the involvement of m^6^A in mouse embryogenesis^41–43^. We carried out two biological replicates of eTAM-seq (mESC/IVT) and detected 24,676 and 26,756 m^6^A sites. Among these, 20,727 are shared and show consistent methylation levels (Pearson’s r = 0.95, Supplementary Note 8 and Supplementary Fig. 17). We sequenced replicate 1 deeper and obtained 65,853 hits, 58,456 of which show exposed methylation levels higher than 10% (Fig. 3a and Supplementary Fig. 18). m^6^A sites in mESCs again reveal a clear DRACH motif (84%) and are enriched around stop codons (Fig. 3b). 30,191 (52%) eTAM-seq-captured m^6^A sites overlap with 6,097 (74%) MeRIP-seq peaks^26^, wherein one MeRIP-seq peak covers 5.0 m^6^A sites averagely (median: 4, Fig. 3c). We also prepared a control mESC transcriptome via FTO treatment. eTAM-seq (mESC/FTO) captured 47,966 sites with exposed methylation levels ≥ 10%. 91% of eTAM-seq (mESC/FTO) hits overlap with eTAM-seq (mESC/IVT) hits and report highly correlated methylation levels (Pearson’s r = 0.995, Fig. 3d).

Methylation of pluripotency transcription factors, Nanog, Sox2, and Klf4, is a hallmark of the naïve pluripotent state^41, 43^ and was clearly captured by eTAM-seq (Fig. 3e). In contrast, a single lowly methylated site persists across two eTAM-seq datasets in Oct4 (22.7±7.6%, Supplementary Fig. 19), another key pluripotency transcription factor and a critical component of the Yamanaka cocktail (OSKM factors)^43, 44^. The absence of significant methylation in Oct4 is consistent with previous reports that the abundance of Oct4 mRNA was not significantly impacted by deleting *Mettl3*, the key m^6^A writer gene^41, 43^. We quantified the modification levels of the 11, 18, and 22 m^6^A sites in Nanog, Sox2, and Klf4, respectively, and found that 1, 7, and 7 of these sites were methylated close to completion (>80%, Fig. 3e and Supplementary Fig. 19). The density and stoichiometries of methylation in these transcripts have never been reported; therefore, we evaluate the fidelity of eTAM-seq by comparing with MeRIP-seq data^26^. We confirm that positions and fractions of m^6^A detected by eTAM-seq recapitulate MeRIP-seq peak clusters (Fig. 3e). Collectively, we successfully applied eTAM-seq to mESCs and detected m^6^A sites in mRNA essential for embryogenesis.

**Figure 4 |:**
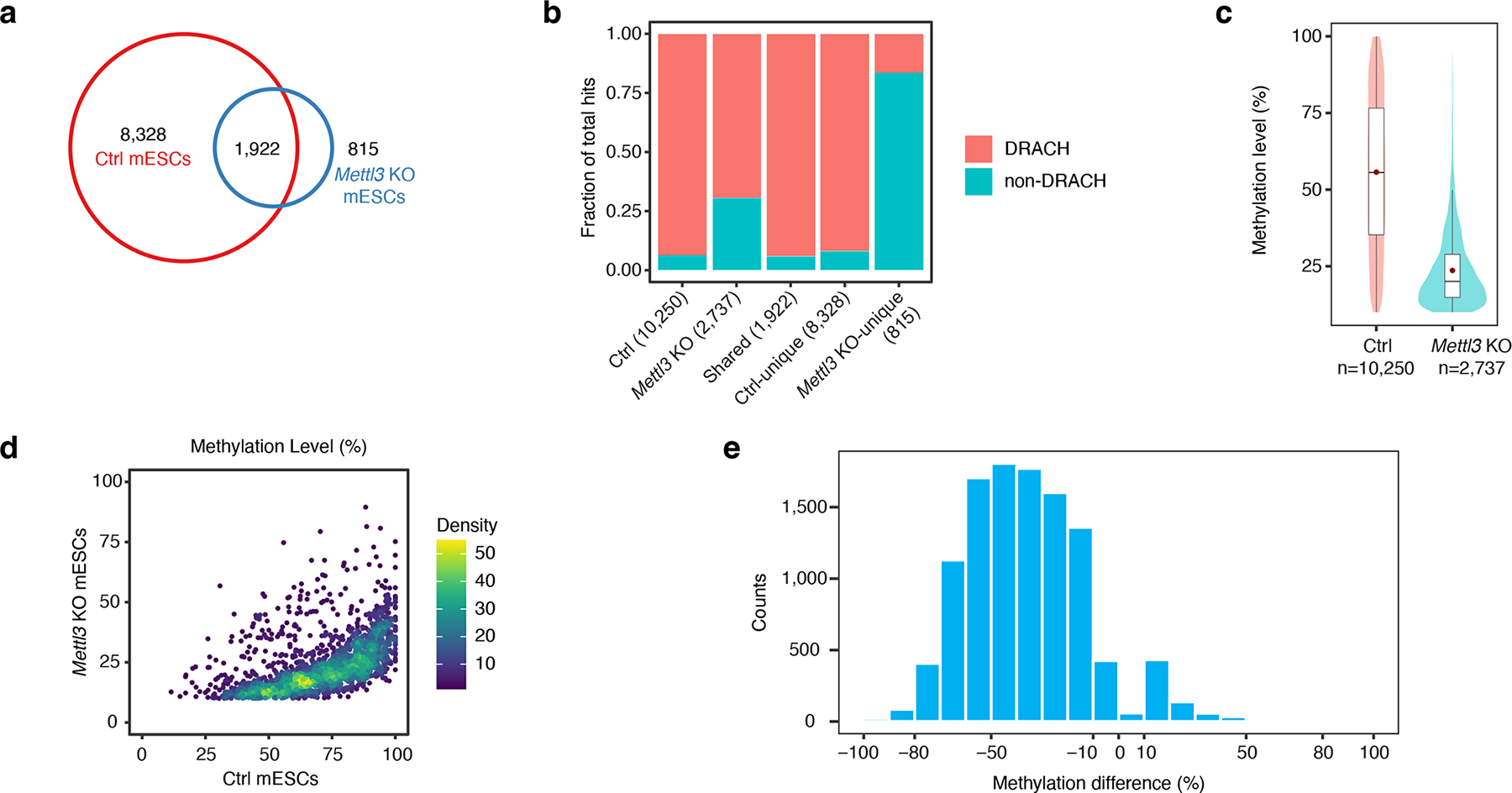
m^6^A is strongly depleted in *Mettl3* KO mESCs. **a**. Venn diagram showing the overlap of eTAM-seq-detected m^6^A sites in ctrl and *Mettl3* KO mESCs. **b**. Hit distributions in DRACH and non-DRACH sequences. **c**. Methylation levels of eTAM-seq-captured m^6^A sites in ctrl and *Mettl3* KO mESCs. Lower and upper hinges in the box plot represent first and third quartiles with the center line and red dot representing the median and the mean, respectively. Whiskers cover ±1.5× of the interquartile range. **d**. Scatter plot of methylation levels for m^6^A sites jointly identified in ctrl and *Mettl3* KO mESCs. **e**. Changes of methylation levels in ctrl and *Mettl3* KO mESCs. Methylation difference for a given A site = methylation level in ctrl mESCs – methylation level in *Mettl3* KO mESCs.

We next generated mESCs (*Mettl3*^*flox/flox*^) in which *Mettl3* can be knocked out by small molecule-induced Cre recombination and applied eTAM-seq to mRNA isolated from *Mettl3* knockout (*Mettl3* KO) mESCs. As a control, we included mESCs wherein Cre-mediated *Mettl3* KO was not induced (ctrl mESCs), i.e., cells with intact m^6^A deposition machinery. Western blot confirms strong depletion of METTL3 in *Mettl3* KO mESCs (Supplementary Fig. 20a). eTAM-seq captured 10,250 m^6^A sites in ctrl mESCs, 9,759 (95%) of which overlap with those identified in wildtype mECSs (Supplementary Fig. 20b and 20c). In contrast, only 2,737 m^6^A sites were identified in *Mettl3* KO mESCs. A moderate overlap was observed among m^6^A sites detected in ctrl and *Mettl3* KO mESCs (1,922, 19% and 70% of ctrl and *Mettl3* KO samples, respectively, Fig. 4a). m^6^A sites detected in ctrl mESCs are almost exclusively found in DRACH motifs (9,602, 94%), whereas 1,900 m^6^A sites (69%) arising in *Mettl3* KO mESCs can be attributed to this consensus motif (Fig. 4b). The median methylation levels in ctrl and *Mettl3* KO mESCs are 55.6% and 20.0%, respectively (Fig. 4c). *Mettl3* KO-specific m^6^A sites (815) show low methylation levels (median: 17.8%) and DRACH occupancy (16%). Global reduction in methylation was clearly observed upon METTL3 depletion (Fig. 4d and 4e). We therefore conclude that METTL3 is the dominant methyltransferase responsible for m^6^A installation on mRNA in mESCs. These results also support the fidelity of eTAM-seq.

### m^6^A loads correlate negatively with mRNA stability

We noted that m^6^A signals tended to cluster in our analyses of individual HeLa and mESC transcripts (Fig. 2i, 3e, and Supplementary Fig. 16). To investigate whether such trends hold true across the HeLa transcriptome, we performed permutation tests under a null hypothesis that the distribution of m^6^A sites is uniform. We sampled 10 times for possible locations of m^6^A (69,834) across all evaluated A sites 1) with no context constraint or 2) forcing m^6^A-carrying 5-nt motifs to match the frequencies observed in eTAM-seq. Median gaps between two neighboring m^6^A sites under these two simulation criteria are 296 and 343 nt, larger than the 62 nt-gap observed in eTAM-seq (Supplementary Fig. 21), supporting the hypothesis that m^6^A has a tendency to cluster.

**Figure 5 |:**
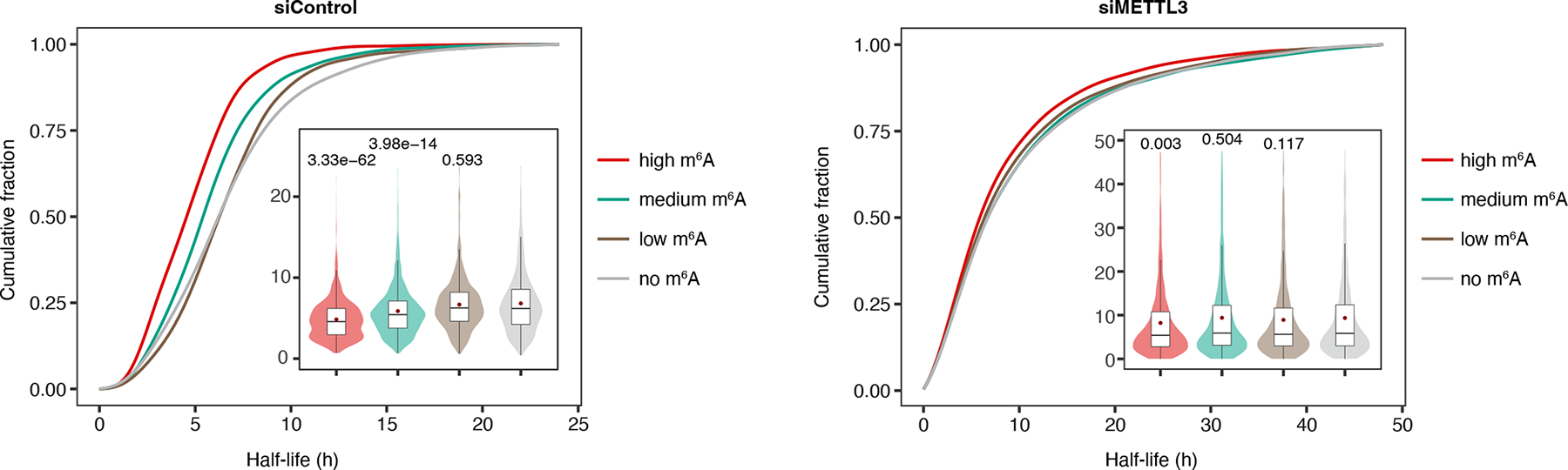
m^6^A impacts transcript stability. Cumulative distributions for transcripts of different half-lives in HeLa cells treated with control and METTL3-targeting siRNA. Transcripts methylated to different levels are analyzed in separate bins (high m^6^A: *n* = 1,593; medium m^6^A: *n* = 1,594; low m^6^A: *n* = 1,593; no m^6^A: *n* = 1,758). Box violin plots of transcript half-lives are inserted. Lower and upper hinges represent first and third quartiles. The center line and the red dot denote the median and the mean, with whiskers covering ± 1.5× of the interquartile range. P-values were determined by one-tailed Wilcoxon rank-sum test using the unmethylated group as a reference. HeLa mRNA half-life dataset: GSE49339^38^.

Previous reports have linked m^6^A with mRNA degradation^38, 45^. We were therefore inspired to examine how the total methylation load on a transcript would impact its stability (HeLa mRNA half-life dataset: GSE49339^38^). We summed methylation levels for individual HeLa mRNA and categorized them into three groups—high, medium, and low methylation, each harboring 1,593 transcripts. A group of 1,758 unmethylated transcripts was included as a reference. The mean half-lives for mRNA bearing high, medium, low, and no methylation were 4.8, 5.9, 6.7, and 6.8 h, respectively, in HeLa cells treated with control siRNA, and were extended to 8.2, 9.4, 8.9, and 9.4 h when METTL3 was knocked down (Fig. 5). While highly methylated mRNA shows an average 29% reduction in half-life compared to unmethylated mRNA in control cells (4.8 versus 6.8 h), the difference largely diminishes in cells with impaired methyltransferase activity, supporting a central role for m^6^A in mediating mRNA decay.

YTHDF proteins, a major family of m^6^A readers, interact with m^6^A-bearing transcripts to exert their regulatory function. Among them, YTHDF2 was reported to drive the degradation of m^6^A-modified transcripts^38, 46–48^. Indeed, the differences in half-life for mRNA of different methylation loads were largely reduced in cells treated with YTHDF2-targeting siRNA (Supplementary Fig. 22), confirming YTHDF2 as a core regulator for the stability of m^6^A-modified mRNA.

### Site-specific m^6^A quantification with limited input

**Figure 6 |:**
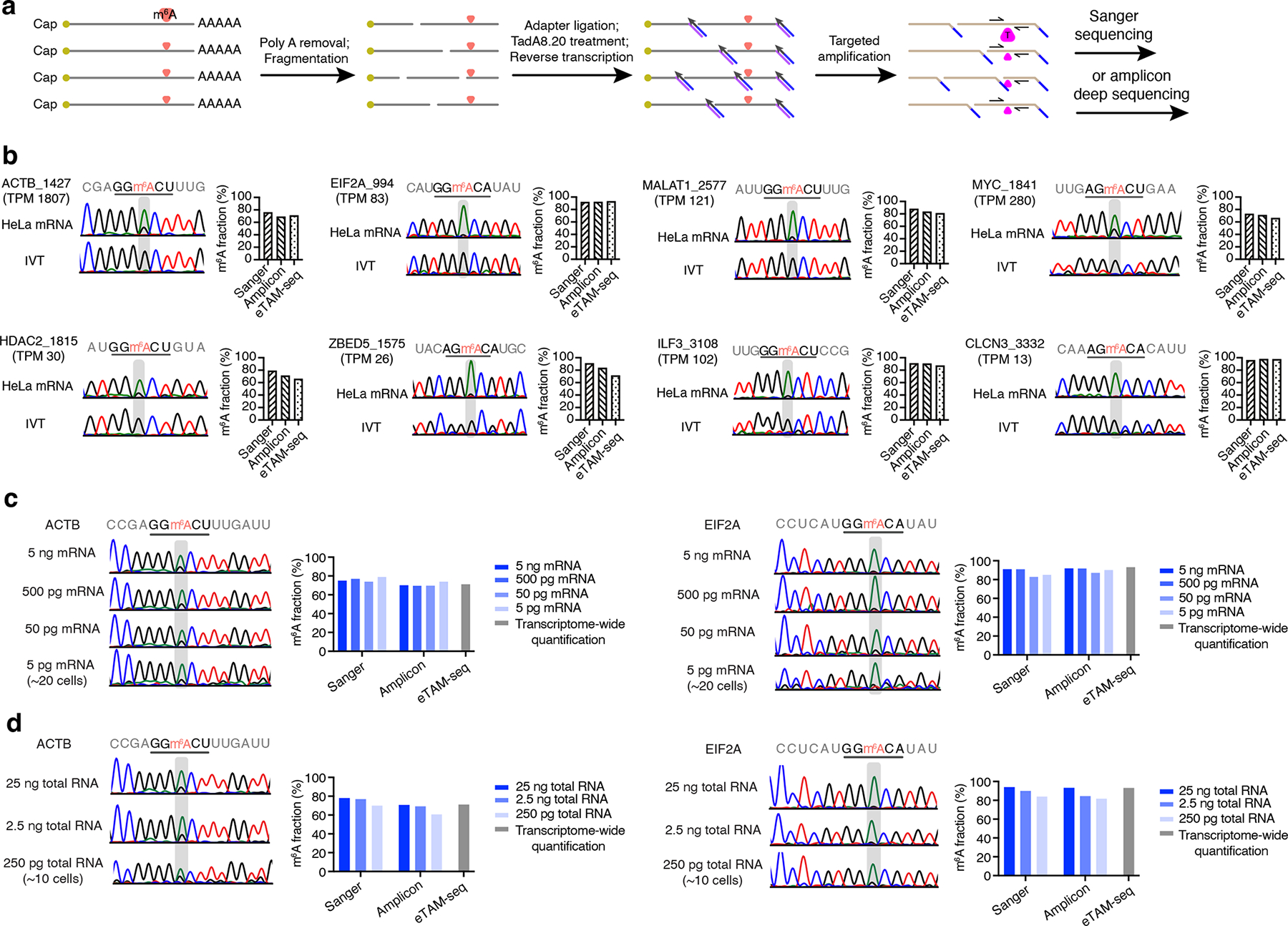
Site-specific, deep sequencing-free m^6^A detection and quantification. **a**. Workflow for eTAM-seq-enabled site-specific quantification of m^6^A. mRNA is fragmented, ligated to a DNA adapter, treated by TadA8.20, and reverse transcribed into cDNA. Site-specific primers are designed to recognize post-deamination RNA sequences and amplify the loci of interest. m^6^A quantification can be achieved by both Sanger sequencing and amplicon deep sequencing. **b**. Quantification of methylation levels for 8 m^6^A sites in HeLa mRNA by Sanger sequencing, amplicon deep sequencing, and RNA-seq. Tad8.20-treated IVT samples are provided for reference only. **c**. Methylation quantification for ACTB_1427 and EIF2A_994 with 5 ng, 500 pg, 50 pg, and 5 pg mRNA. **d**. Methylation quantification for ACTB_1427 and EIF2A_994 with 25 ng, 2.5 ng, and 250 pg total RNA.

eTAM-seq offers a straightforward approach for site-specific detection and quantification of m^6^A, similar to bisulfite sequencing that has been widely applied in assessing DNA methylation at promoter and enhancer sites. We developed a streamlined protocol for site-specific, deep sequencing-free m^6^A quantification: fragmentation, global A deamination, reverse transcription, polymerase chain reaction, and Sanger sequencing (Fig. 6a and Supplementary Fig. 23a). We applied this eTAM-Sanger protocol to 18 m^6^A sites (methylation level: 60–99%, median: 83%) in HeLa transcripts of high, medium, and low abundances (Supplementary Tables 3 and 4). We employed EditR^49^, a program developed to analyze base-editing outcomes from Sanger sequencing data, to quantify the A and G levels in Sanger traces (Supplementary Note 9). To assess fidelity of m^6^A quantification by Sanger sequencing, we simultaneously analyzed the amplicons by small-scale deep sequencing, with 10k reads allocated to each sample.

Methylation levels quantified by whole-transcriptome eTAM-seq were used as references to evaluate eTAM-Sanger results. For all 18 inspected sites, eTAM-Sanger reported methylation levels within small deviations from whole-transcriptome eTAM-seq estimates (0.7%–20%, median deviation: 5.9%, Fig. 6b, Supplementary Fig. 23b and 23c). Methylation stoichiometries were detected within ± 10% for 15 of the 18 sites. We note that it was often challenging to quantify low levels of G signals in Sanger traces, which was a major source of error in eTAM-Sanger. Indeed, amplicon deep sequencing, which is more suited to quantifying mixed base signals, further improved the accuracy, delivering methylation estimates with a median deviation of 2.4% (0.3%–7.2%) from whole-transcriptome eTAM-seq results (Fig. 6b, Supplementary Fig. 23b and 23c). We therefore conclude eTAM-seq supports faithful m^6^A quantification regardless of the readout method—Sanger sequencing, amplicon deep sequencing, or whole-transcriptome sequencing.

Control samples are not a prerequisite for site-specific m^6^A quantification. We determined methylation stoichiometries for JUNB_1352, GRWD1_1487, H2AFX_1331, and PPIB_823 in the absence of IVT samples (Supplementary Fig. 23c). Importantly, m^6^A quantification was achieved for low abundance mRNA, such as CLCN3 (Transcripts Per Million, TPM: 13, Fig. 6b)^24^, confirming that, unlike probe-mediated detection mechanisms which are sensitive towards abundant transcripts, eTAM-Sanger quantifies m^6^A in transcripts spanning a broad range of abundances (TPM 13–1,807).

To assess the detection limit of eTAM-Sanger, we attempted to amplify two m^6^A-bearing sites in ACTB and EIF2A from diluted cDNA samples. We successfully obtained PCR products from cDNA corresponding to 500 pg, 50 pg, and 5 pg starting mRNA for both sites (Supplementary Fig. 24), which produced Sanger traces almost identical to those started with 5 ng mRNA (Fig. 6c). Encourage by these observations, we next exploited eTAM-Sanger for site-specific m^6^A quantification from total RNA. Although rRNA was not depleted in these samples, which is expected to compromise enzymatic treatment, reverse transcription, and target site amplification, we successfully obtained short DNA fragments covering ACTB_1427 and EIF2A_994 with 25 ng, 2.5 ng, and 250 pg total RNA (Supplementary Fig. 24), which corresponds to approximately 1,000, 100, and 10 cells, respectively. These amplicons generated Sanger traces similar to cDNA synthesized from 5 ng mRNA (Fig. 6d). We quantified the methylation levels to be 70–78% for ACTB_1427 and 84–94% for EIF2A_994, within 7% and 9% differences from the levels reported by whole-transcriptome eTAM-seq. Collectively, we showcase reliable, deep sequencing-free m^6^A quantification with ultra-low sample input.

## Discussion

We present in this work a new m^6^A sequencing method, eTAM-seq, that functions through enzyme-assisted A deamination. The comprehensive m^6^A maps generated by eTAM-seq enable us to inspect the distribution and function of m^6^A at unprecedented resolution. We observe a close-to-even distribution for m^6^A sites across different methylation levels in both HeLa and mES cells. We note that eTAM-seq is less sensitive to sites of low methylation levels. There could be numerous lowly modified sites; however, our results suggest that moderately to highly methylated A sites are likely major contributors to the cellular m^6^A pool.

One critical challenge faced by existing m^6^A profiling methods is their reliance on bulk input materials. Almost all methods reported so far suffer from RNA loss during sample preparation. In contrast, eTAM-seq employs an enzymatic deamination mechanism, which, unlike chemical deamination conditions that tend to degrade the input RNA^50^, maintains RNA integrity with no measurable degradation. Low detection limit is a major advantage of eTAM-seq. We demonstrate in this work m^6^A detection and quantification with as few as 10 cells. We envision future efforts extending to single-cell level detection. As eTAM-seq sustains gene expression information captured by canonical RNA-seq, single-cell eTAM-seq may report transcript abundances and m^6^A modifications simultaneously in the same cell.

As an mRNA modification, m^6^A is subject to cellular regulation via active deposition and removal. There is high demand in the epitranscriptomics field for a robust method that informs on the presence and stoichiometry of methylation at sites of interest. We showcase in this work site-specific, deep sequencing-free m^6^A detection with eTAM-Sanger. Our workflow employs routine molecular biology assays such as reverse transcription, PCR, and Sanger sequencing without relying on specialized laboratory techniques, lowering the barrier of m^6^A detection and quantification. Faithful m^6^A quantification has been achieved with as little as 250 pg total RNA, a detection limit orders of magnitude lower than existing quantitative profiling methods. We envision eTAM-seq will allow researchers to routinely survey dynamics of m^6^A in their biological processes of interest.

Deaminase accessibility is a prerequisite for m^6^A detection using eTAM-seq; as such, eTAM-seq may not work well for highly structured RNA. Nevertheless, previous work has shown that most m^6^A sites reside in non-structured RNA regions^51, 52^. We also demonstrate that only 8% and 12% of A sites in the HeLa and mESC transcriptomes, respectively, show accessibility < 0.9. Therefore, we expect the vast majority of mammalian transcriptomes to be sensitive to eTAM-seq.

Stoichiometry estimation by eTAM-seq may be less accurate at lowly methylated sites (e.g., < 25%) or sites of compromised accessibility. We employed control transcriptomes prepared via two orthogonal routes to assess accessibility of given A sites to the deaminase: an *in vitro* transcribed modification-free transcriptome and an FTO-treated *N*^6^-demethylated transcriptome. Both control transcriptomes provide reliable accessibility estimates to enable faithful m^6^A mapping. We propose that both controls are valuable, although for most studies, researchers only need one control to map the positions and fractions of m^6^A. Note that these control samples need to be prepared and sequenced only once for each cell type. For example, the accessibility estimates reported in this work may be directly applied to map m^6^A in HeLa and mESC cells.

Other adenine modifications may also resist TadA8.20-catalyzed deamination and generate eTAM-seq signals. However, m^6^A is much more abundant than other known adenosine modifications in mammalian mRNA and should therefore be responsible for the majority of eTAM-seq signals. Genuine methylation sites can be further confirmed using the dedicated demethylase FTO. Lastly, eTAM-seq can be adapted to map and quantify other adenine modifications in mammalian RNA if the aimed modification can be selectively removed to generate proper controls.

## Online Methods

### Cell culture

Human HeLa cells and mouse embryonic stem cells (mESC) were purchased from ATCC. HeLa cells were grown in DMEM (Gibco, 11965092) media supplemented with 10% FBS (Gibco) and 1% 100× Pen/Strep (Gibco). WT, control knockout, and *Mettl3* conditional knockout (cKO) mESCs were maintained in DMEM (Invitrogen) supplemented with 15% FBS (Gibco), 1% nucleosides (100×) (Millipore), 1 mM L-glutamine (Gibco), 1% nonessential amino acids (Gibco), 0.1 mM 2-mercaptoethanol (Sigma), 1,000 U/ml LIF (Millipore), 3 μM CHIR99021 (Stemcell), and 1 μM PD0325901 (Stemcell). All cells were cultured at 37 °C under 5.0% CO_2_.

*Mettl3* cKO mES cell lines were generated following previously reported methods^53^. Briefly, mESCs derived from *Mettl3*^*flox/flox*^ mouse blastocyst were transfected with 200 ng PB-CAG-Puromycin-P2A-CreERT2 and 100 ng PBase by electroporation. After 24 h, electroporated cells were treated with 1 μg/ml Puromycin to generate stable *Mettl3*^*flox/flox*^; *CreERT2* mES clones. To induce deletion, *Mettl3*^*flox/flox*^; *CreERT2* ESC cells were treated with 1 μg/ml 4-hydroxytamoxifen (Sigma). These *Mettl3* KO cells were cultured for 48 h before harvesting. Untreated *Mettl3*^*flox/flox*^; *CreERT2* ESC cells were used as ctrl mESCs.

### Western blot

Cells were lysed in RIPA lysis buffer (Pierce) supplemented with complete protease inhibitor cocktail (Takara). Lysates were boiled at 95 °C in NuPAGE LDS loading buffer (Invitrogen) for 10 min and then stored at −80 °C for use in the next step. A total of 30 μg protein per sample was loaded into 4–12% NuPAGE Bis-Tris gel and transferred to PVDF membranes (Life Technologies). Membranes were blocked in 5% milk PBST for 30 min at room temperature (RT), incubated in 1:1000 (v/v) dilution of anti-METTL3 antibody (abcam, ab195352) at 4 °C overnight, washed, and incubated in 1:5000 (v/v) dilution of goat anti rabbit igG-HRP (abcam, ab6721) for 1 h at RT. Membrane region lower than 50 kD were used as loading control and directly washed and incubated in 1:1000 (v/v) dilution of anti-GAPDH mAb-HRP (CST, 3683) for 1 h at RT. Protein bands were detected using SuperSignal West Dura Extended Duration Substrate kit (Thermo) and FluroChem R (Proteinsimple).

### Poly-A RNA extraction

Cells were cultured to 70–80% confluency, rinsed with 1× PBS (Gibco), and lysed by the direct addition of TRIzol reagent (Invitrogen). Total RNA was then collected following the manufacturer’s protocol. Poly A^+^ RNA was extracted from purified total RNA using Dynabeads mRNA DIRECT Purification Kits (Invitrogen).

### Overexpression and purification of recombinant TadA8.20 protein

Wild type TadA and TadA8.20 fused to an N-terminal hexahistidine-tagged maltose binding protein (6xHis-MBP) were cloned into a pET28a vector. A TEV protease cleavage site (ENLYFQ|G) was installed between MBP and TadA variants. Expression plasmids will be deposited to Addgene.

BL21 Rosetta 2 (DE3) competent cells were transformed with the recombinant plasmids and grown on Luria broth (LB) agar plates supplemented with 50 μg/mL kanamycin and 25 μg/mL chloramphenicol. Successfully transformed bacteria were always cultured in the presence of 50 μg/mL kanamycin and 25 μg/mL chloramphenicol unless otherwise noted. Single colonies were inoculated into fresh LB medium and grown in an incubator shaker (37°C, 220 rpm) for 12–18 h. A 10 mL saturated start culture was used to inoculate 1 L fresh medium. Bacteria were grown at 37°C until OD_600_ reached 0.5. The culture was cooled down immediately to 4°C and induced with 0.1 mM isopropyl β-d-1-thiogalactopyranoside (IPTG). Bacteria were cultured at 16°C for an additional 20 h before pelleting by centrifugation at 4,000 g.

Bacterial pellets were lysed by sonication in buffer A (50 mM Tris, 500 mM NaCl, 10 mM β-mercaptoethanol, and 10% (v/v) glycerol; pH 7.5). Lysed bacteria were clarified by centrifugation at 4°C, 23,000 g. The supernatant was loaded onto a Ni-NTA Superflow Cartridge (Qiagen, 30761), washed with 30 mL of buffer A supplemented with 50 mM imidazole, and eluted with a gradient of imidazole from 50 mM to 500 mM in buffer A.

The eluted protein was incubated with TEV protease and dialyzed in buffer A at 4°C overnight. The protein mixture was reloaded onto a Ni-NTA Superflow Cartridge, washed with buffer B (50 mM Tris, 1 M NaCl, 10 mM β-mercaptoethanol, and 10% (v/v) glycerol; pH 8.0), and eluted by buffer B supplemented with 50 mM imidazole. Finally, MBP-free TadA8.20 was purified by size-exclusion chromatography (Enrich^™^ SEC 650 10 × 300 mm Column, Bio-Rad, 7801650) and concentrated to approximately 4 mg/mL. The column was balanced and eluted with buffer C (50 mM Tris, 200 mM NaCl, 10 mM β-mercaptoethanol, and 10% (v/v) glycerol; pH 7.5).

### Preparation of A- and m^6^A-bearing *E. coli* tRNA (Arg2, CGT) and RNA probes

Double-stranded DNA templates carrying T7 promoter were prepared by primer extension with two single-stranded DNA oligos (Supplementary Tables 5 and 6). Unmethylated and methylated *E. coli* tRNA (Arg2, CGT), RNA#1, and RNA#2 were synthesized by *in vitro* transcription using T7 RNA polymerase. ATP and *N*^6^-methyl-ATP (TriLink, N-1013) were supplied in the presence of UTP, CTP, and GTP to synthesize unmethylated and methylated RNA, respectively. RNA was purified by E.Z.N.A Micro RNA kits (Omega Bio-Tek, R7034) and quantified by NanoDrop One (Thermo Fisher Scientific).

### Spike-in probes synthesis

Spike-in probes were synthesized and pooled according to a previously published protocol^14^. Sequences for spike-in probes are listed in Supplementary Table 7. Probes 1–8 were mixed in a ratio (w/w) of 20%, 15%, 10%, 5%, 5%, 10%, 15%, 20%, respectively. The final probe mixture is composed of 5 sets of UMI-labeled RNA oligos of 0/25/50/75/100% m^6^A.

### Preparation of IVT transcriptomes

Modification-free control RNAs were prepared from HeLa and mESC total mRNA based on previously published protocols^26, 54^ with minor modifications. Oligo-dT(30)VN primer (TTTTTTTTTTTTTTTTTTTTTTTTTTTTTTVN, 100 pmol) was annealed to 100 ng of purified poly A^+^ RNA at 65°C for 5 min. RNA was reverse transcribed in 20 μL 1× RT buffer (Thermo Scientific, EP0753, containing 50 mM Tris-HCl, pH 8.3; 75 mM KCl, 3 mM MgCl_2_, 10 mM DTT) in the presence of 40 pmol of 5Bio-T7-TSO (/5Biosg/ACTCTAATACGACTCACTATAGGGAGAGGGCrGrGrG), 1 mM of GTP, 5% (w/v) PEG 8000, 0.5 mM each of dNTP, 5 mM of RNaseOUT (Invitrogen, 10777019), and 200 U of Maxima H^−^ Reverse Transcriptase (Thermo Scientific, EP0753) under the following conditions: 42°C for 90 min, 10 cycles of [50°C for 2 min plus 42°C for 2 min], 85°C for 5 min. To the 20 μL of RT reaction, 10 μL of RNase H (NEB, M0297L), 70 μL of RNase-free H_2_O, and 100 μL of Ultra II Q5 Master Mix (NEB, M0544X) were added to make the second-strand synthesis mixture, which was incubated under the following conditions: 37°C for 15 min, 95°C for 1 min, 65°C for 10 min. The reaction was purified with 160 μL (0.8×, v/v) of AMPure XP beads (Beckman Coulter, A63882) following the manufacturer’s directions.

The purified and concentrated dsDNA was *in vitro* transcribed (IVT) in 1× T7 Reaction Buffer (NEB E2040S, containing 40 mM Tris-HCl, 6 mM MgCl_2_, 1 mM DTT, 2 mM spermidine, pH 7.9) with 10 mM of each NTP, and 2 μL of T7 RNA Polymerase Mix (NEB E2040S) in 20 μL volume at 37°C overnight. The IVT mixture was further treated with TURBO DNase (Invitrogen, AM2238) and purified by acid-phenol chloroform (Invitrogen, AM9722) extraction and ethanol precipitation to yield 2.5–10 μg of IVT RNA.

### *In vitro* deamination of RNA probes by TadA8.20

All reactions were carried out in a deamination buffer (50 mM Tris, 25 mM KCl, 2.5 mM MgCl_2_, 2 mM dithiothreitol, and 10% (v/v) glycerol; pH 7.5) in the presence of 10 U SUPERase•In^™^ RNase Inhibitor (Thermo Fisher Scientific, AM2694). RNA was always preheated to 95°C for 3 min and immediately cooled down before use.

To assay deaminase activity on the natural substrate *E. coli* tRNA, 200 ng RNA and 100 nM wild type TadA or TadA8.20 were incubated at 37°C for 1 h. For a typical deamination assay on RNA probes, 10 ng RNA was incubated with 10 μM TadA8.20 in 20 μL deamination buffer at 37°C for 3 h. All reactions were quenched by incubating at 95°C for 10 min. Temperature and pH were adjusted to identify the optimal condition for *in vitro* deamination (Supplementary Fig. 2).

### Reverse transcription polymerase chain reaction (RT-PCR)

To convert RNA into complementary DNA for sequencing purposes, a 2 μL deamination reaction was aliquoted, to which 0.5 μL of 50 μM reverse transcription primer was supplied. Primer annealing was enabled by heating up the mixture to 95°C for 3 min, cooling down at a ramping rate of 2°C/s, and incubation at 25°C for 2 min. To the reaction, 0.5 μL of GoScript reverse transcriptase (Promega, A5003) was added together with 2 μL of 5x GoScript RT buffer, 1 μL of 25 mM MgCl_2_, 0.5 μL of 10 mM dNTPs, and 3.5 μL nuclease-free H_2_O. The reverse transcription reaction was incubated at 42°C for 1 h and then quenched at 65°C for 20 min.

To a 20 μL PCR or quantitative PCR reaction (EvaGreen qPCR Master Mix, Biotium 31041), 0.1 μL of the reverse transcription reaction was supplied as template. A typical PCR program includes initiation at 95°C for 3 min; 30 cycles of amplification (denaturing at 95°C for 10 s, annealing at 60°C for 10 s followed by extension at 72°C for 20 s); and final extension at 72°C for 5 min. qPCR reactions were performed on a CFX96^™^ Real-Time PCR System (Bio-Rad).

Sequences of primers for reverse transcription, site-specific amplification, and Illumina adapter installation are listed in Supplementary Tables 8–10.

### Overexpression and purification of recombinant FTO

The human FTO gene was cloned into a pET28a vector and transformed into BL21(DE3) cells (NEB). Successfully transformed bacteria were cultured at 37°C in 2xYT broth Teknova) to an O.D. of 0.8–1.0. The culture was cooled to 16°C and supplemented with 0.1 mM IPTG (Sigma), 10 μM ZnSO_4_ (sigma), and 2 μM (NH_4_)_2_Fe(SO_4_)_2_ (sigma). Bacteria were cultured overnight at 16°C following induction.

Bacteria were collected via centrifugation and lysed in buffer D (300 mM NaCl (Fisher), 50 mM imidazole (Fisher), and 50 mM of Na_2_HPO_4_ (Sigma); pH 8.0). The lysate was clarified by centrifugation and loaded onto a nickel column (Ni Sepharose 6 FF, Cytiva), washed with buffer E (150 mM NaCl, 25 mM imidazole, and 10 mM Tris-HCl (Invitrogen); pH 7.5), and eluted with buffer F(150 mM NaCl, 250 mM imidazole, and 10 mM Tris-HCl; pH 7.5). The eluate was loaded onto an anion-exchange column (SOURCE 15Q, Cytiva) and fractionated with 0–50% of Buffer G (1.5 M NaCl, 20 mM Tris-HCl; pH 7.5) over 30 min. The resulting protein was concentrated and buffer exchanged using a 10 kD MWCO filter (Cat. No. 28932296, Cytiva) before being flash frozen in 30% glycerol for future use.

### eTAM-seq library preparation

50 ng of purified poly A^+^ RNA and 25 ng of IVT control RNA from HeLa or mES cells were depleted of poly A tails, end-repaired, and ligated to 3’ adapters following a previously published protocol^14^. Briefly, RNA was annealed to 100 pmol of oligo-dT (Thermo Scientific, SO132) and digested by 5 U of RNase H (NEB, M0297L) at 37°C for 30 min. Without purification, the RNA was fragmented in 1× Zinc fragmentation buffer (10 mM ZnCl_2_ and 10 mM Tris-HCl, pH 7.5) at 70°C for 5 min. The fragmentation reaction was quenched by the addition of 10 mM EDTA, then treated with 50 U of T4 PNK (NEB, M0201L) at 37°C for 1 h. The end-repaired RNA was purified by RNA Clean & Concentrator (Zymo) kits, mixed with 2% (w/w) of spike-in probes, and ligated to 20 pmol of 3’ adapter (/5rApp/AGATCGGAAGAGCGTCGTG/3Bio/) using 400 U of T4 RNA ligase 2, truncated KQ (NEB, M0373L) at 25°C for 2 h, then 16°C overnight. Excess adapters were first digested by 5’ Deadenylase (NEB, M0331S) at 30°C for 1h, then by RecJ_f_ (NEB, M0264L) at 37°C for 1h.

The ligated poly A^+^ RNA was divided into two halves and designated to the FTO^−^ and FTO^+^ groups. The FTO^−^, FTO^+^, and IVT groups were immobilized on Dynabeads MyOne Streptavidin C1 (Invitrogen, 65002). The FTO^+^ group was demethylated by incubating with 200 pmol of FTO in 1× FTO reaction buffer (2 mM sodium ascorbate (Sigma), 65 μM ammonium iron(II) sulfate (Sigma), 0.3 mM α-ketoglutarate (Sigma), 0.1 mg/mL BSA (NEB), and 50 mM HEPES-KOH; pH 7.0) supplemented with 10% (v/v) of SUPERase•In RNase Inhibitor (Invitrogen) at 37 °C for 1 h. The beads were washed by resuspension in 0.1% PBST (1× PBS (Gibco) supplemented with 0.1% (v/v) tween 20 (Sigma)), 1x Binding/Wash buffer (1 M NaCl, 0.5 mM EDTA, 5 mM Tris-HCl; pH 7.5), and twice with 10 mM Tris HCl (pH 7.5), consecutively.

The three RNA samples were then deaminated on beads with 200 pmol of TadA8.20 in the deamination buffer (50 mM Tris, 25 mM KCl, 2.5 mM MgCl_2_, 2 mM dithiothreitol, and 10 % (v/v) glycerol; pH 7.5) supplemented with 10% (v/v) of SUPERase•In RNase Inhibitor (Invitrogen, AM2696) at 53°C for 1 h. This reaction was repeated twice at 44°C, 1h each by draining the supernatant on a magnetic rack and resuspending the beads in fresh reaction mixtures, lasting 3 h in total. The beads were washed sequentially by resuspension in 0.1% PBST (v/v), 1× Binding/Wash buffer, and twice in 10 mM Tris HCl (pH 7.5).

RNA was annealed to 2 pmol of RT primer (ACACGACGCTCTTCCGATCT) at 70°C for 2 min, then reverse transcribed in 1× RT buffer (Thermo Scientific, EP0753, containing 50 mM Tris-HCl, pH 8.3; 75 mM KCl, 3 mM MgCl_2_, 10 mM DTT) with 5 mM of RNaseOUT (Invitrogen, 10777019) and 200 U of Maxima H^−^ Reverse Transcriptase (Thermo Scientific, EP0753) at 50°C for 1h. The cDNA was released by boiling the beads in 0.5% (v/v) SDS for 10 min. The eluate was purified by DNA Clean & Concentrator (Zymo) kits. Purified cDNA was ligated to cDNA Adapter (/5Phos/NNNNNN AGATCGGAAGAGCACACGTCTG/3SpC3/) using 30 U of T4 RNA ligase (NEB) at 25°C overnight, following a previously published protocol^14^. The reaction was purified again by DNA Clean & Concentrator (Zymo) kits and then PCR amplified with NEBNext Ultra II Q5 Master Mix (NEB, M0544X) and NEBNext Unique Dual Index Primer for Illumina (NEB, E6440S) following the manufacturer’s directions. Typically, 10–11 cycles of PCR were carried out to generate enough DNA. The resulting library was purified by AMPure XP beads (Beckman Coulter, A63882) following the manufacturer’s directions and submitted for next-generation sequencing.

### Site-specific amplification and barcoding of HeLa mRNA and IVT samples

Purified HeLa poly A^+^ RNA (300 ng) was depleted of poly A tails, end-repaired, ligated to 3’ adapters, and immobilized on Dynabeads MyOne Streptavidin C1 (Invitrogen) as described in eTAM-seq library preparation. RNA was deaminated and reverse transcribed following a similar protocol for NGS library construction. Specifically, RNA samples were deaminated on beads with 200 pmol of TadA8.20 in the deamination buffer (50 mM Tris, 25 mM KCl, 2.5 mM MgCl_2_, 2 mM dithiothreitol, and 10 % (v/v) glycerol; pH 7.5) supplemented with 10% (v/v) of SUPERase•In RNase Inhibitor (Invitrogen) at 37°C for 1 h. This reaction was repeated twice at by draining the supernatant on a magnetic rack and resuspending the beads in fresh reaction mixtures, lasting 3 h in total. The beads were washed by resuspension in 0.1% PBST (v/v), 1× Binding/Wash buffer, and twice in 10 mM Tris HCl, pH 7.5, consecutively. cDNA was eluted by boiling the beads in DNase-free water (Invitrogen) and transferred into new tubes immediately. Sites of interest were PCR amplified from the eluted cDNA using transcript-specific primers.

To demonstrate m^6^A quantification with limited input, the same protocol was applied to 50 ng, 5 ng, or 500 pg of HeLa total RNA. The resulting cDNA was split into two halves to amply ACTB and EIF2A fragments.

Both rounds of PCR were set up using EvaGreen qPCR Master Mix. 1^st^ round PCR was carried out at a 20 μL scale with cDNA generated from different amounts of mRNA or total RNA, and 0.5 μM forward and reverse primers. Primers were designed to recognize sequences post deamination, leaving out 10–20 nt sequences surrounding the target m^6^A sites. PCR reactions were analyzed by agarose gel electrophoresis. 1 μL of PCR products were subjected to enzymatic cleanup (Exonuclease I and Shrimp Alkaline Phosphatase, New England BioLabs, M0293 and M0371) and Sanger sequencing. Gel purification was performed for PCR reactions that did not yield bright and single bands.

2^nd^ round PCR was carried out only for small-scale amplicon deep sequencing. To a 20 μL reaction, 1 μL of the 1^st^ round PCR reaction was supplied as the template together with 0.5 μM Illumina P7 and P5 index primers. Barcoded PCR products were pooled and gel purified using QIAquick Gel Extraction Kit (Qiagen, 28706) before being subjected to next-generation sequencing on an Illumina MiSeq Instrument.

### eTAM-seq data pre-processing

Adapters were removed from raw eTAM-seq data by Cutadapt v1.18^55^. The 6-nt random barcodes at the 5’ end of R2 were extracted by the subcommand extract from UMI-tools^56^ v1.1.1. R2 reads longer than 39 nt were used for further analysis. For HeLa samples, reads were first mapped to human rRNA sequences using HISAT-3N^57^ v2.2.1 (--time --base-change A,G --no-spliced-alignment --no-softclip --norc --no-unal --rna-strandness F). The remaining non-rRNA reads were mapped to the human genome (hg38) and the GENCODE v27 gene annotation (--time --base-change A,G --repeat --repeat-limit 1000 --bowtie2-dp 0 --no-unal --rna-strandness F). For mESC samples, reads were first mapped to mouse rRNA sequences using HISAT-3N v2.2.1, with the remaining non-rRNA reads mapped to the mouse genome (mm10) and the GENCODE vM25 gene annotation. We used the same HSAT-3N parameters for HeLa and mESC data. Only uniquely mapped reads were kept and deduplicated by the subcommand dedup from UMI-tools. To apply the statistical models for m^6^A detection and quantification, we used high-quality datasets in which RNA fragments poorly processed by TadA8.20 (>50% unconverted A) were eliminated. Custom scripts were developed to count converted and unconverted As across the whole genome.

### MeRIP-seq data processing

Adapters in HeLa MeRIP-seq data (GSE46705^24^) were removed by Cutadapt v1.18^55^. Adapter-free reads were mapped to the human genome (hg38) using HISAT2 v 2.1.0^58^ with default parameters. Aligned sequences were divided into different strands and m^6^A peaks were called using MACS2 v2.1.1^59^ with the following parameters: -f BAM -B --SPMR --nomodel --tsize 50 --extsize 150 --keep-dup all. Peaks meeting the cutoff (fold enrichment > 2 and p-value < 0.05) were kept for further analysis. For mESC MeRIP-seq data, called peaks were downloaded from supplementary files of GSE151028^26^. Common peaks from two replicates were kept for further analysis.

### Identification of endogenous RNA-editing sites

RNA-seq data of two HeLa biological replicates (ENCSR000CPR) were downloaded from the ENCODE Project website (https://www.encodeproject.org/). Data processing followed pipelines described in previous reports^60–62^ with minor adjustments. Briefly, the first 5 nt bases of all raw reads were clipped. The trimmed reads were mapped to rRNA using Bowtie2 v2.4.5^63^, with remaining reads mapped to small RNAs. Reads that did not map to rRNA and small RNAs were then mapped to the genome (hg38) by STAR v2.7.9a^64^. Reads that failed to map in proper pairs or did not map into primary alignment were discarded. PCR duplicates were also removed at this stage. The remaining reads from two replicates were merged and subject to mutation calling from the pileup format data. During mutation calling, the following filters were applied to identify high-quality and high-confidence RNA-editing sites: (1) only sites with read mapping quality ≥ 20 and base quality ≥ 25 were considered; (2) sites mapped to common genomic variants in dbSNP (v151) and gnomAD (v3.1.1) with allele frequency > 5% were discarded; (3) sites within 5 bp of known splice junctions were removed; (4) Alu sites were called with read coverage ≥ 10 and mutation rate ≥ 5%; (5) non-Alu sites were called with variant reads ≥ 4 and mutation rate ≥ 10%; (6) non-Alu sites in simple repeats according to the Repeat Masker annotation were discarded; (7) non-Alu sites were subject to the BLAT correction according to previous studies^60, 61^.

### m^6^A detection and quantification from eTAM-seq data

#### Detection with IVT controls.

We first estimated sample-specific conversion rates for mRNA and IVT samples using the majority of A sites, based on the hypothesis that most A sites are unmethylated and accessible to TadA8.20. We plugged in the sample-specific conversion rate to normalize the A and G counts observed at individual A sites in the mRNA sample and calculated their apparent methylation levels using a maximum likelihood estimator based on a binomial model. Meanwhile, we estimated deaminase accessibility for each A site using adjusted A and G counts reported by the IVT sample. We fitted a linear model between site accessibility and total counts of A, G, A+G observed at individual A sites to shrink accessibility estimates and reduce estimation bias. The model was trained using 2,000 randomly sampled sites with 10-fold cross-validation prior to being applied to predict site accessibility. We adjusted the apparent methylation levels calculated from mRNA data with site accessibility and obtained the final estimated methylation levels (true methylation levels). Methylation sites are defined as A sites that 1) have ≥ 10 read counts in both mRNA and IVT samples; 2) pass Fisher tests with a false discovery rate (FDR) < 0.05; 3) show exposed methylation levels (estimated methylation level * site accessibility) ≥ 10%.

#### Detection with FTO controls.

We first estimated sample-specific conversion rates similar to “detection with IVT controls”. As FTO partially demethylated m^6^A, we further estimated an upper bound for FTO efficiency in FTO-treated samples. Assuming a binomial model for both mRNA and FTO-treated mRNA, we jointly estimated site accessibility and methylation levels using a maximum likelihood approach. Methylation sites are defined with the same criteria as described in “detection with IVT controls”.

All functions are implemented in R. Statistical models are detailed in Supplementary Notes 3 and 6.

### Analysis of the impact of m^6^A on mRNA stability

The half-lives of HeLa mRNA were profiled previously via actinomycin D-mediated transcription inhibition followed by RNA-seq at different time points (GSE49339)^38^. Adapters were removed from RNA-seq data by Cutadapt v1.18^55^. Reads were then mapped to the human genome (hg38) using HISAT2 v 2.1.0^58^ with default parameters. Raw reads of each gene were counted by featureCounts from Subread v1.6.4^65^ and normalized by sequencing depth and gene length using the transcripts per million (TPM) method. The fraction of the spike-in RNA in total sequenced RNA is calculated using the following equation: *y* = *d*⋅𝑣⋅*c*/*t*, where *d* is the dilution factor of spike-in added to each RNA sample, 𝑣 is the volume (in microliter) of diluted spike-in RNA, *c* is the concentration (in attomole per microliter) of each spike-in, and *t* is the mass of total RNA in each sample (in microgram). The TPM values for External RNA Controls Consortium (ERCC) spike-in were correlated to their amounts (in attomole) to build a linear regression model in R 3.5.1, y = ax + b, in which y is the amount of the spike-in RNA (in log2 form), and x is the TPM value (in log2 form). The best-fit dose-response curve in each sample was used to estimate the amount of mRNA (in attomole) for each gene, which was subsequently applied to calculate mRNA half-lives following a previously described protocol^66^.

Methylation loads were calculated by integrating all methylation signals detected in given mRNA. For example, a transcript with three m^6^A sites of 0.3, 0.7, and 1.0 methylation corresponds to a methylation load of 0.3+0.7+1.0 = 2.0. We then ranked all methylated transcripts by their methylation loads. Low, medium, and high methylation bins were defined by transcript ranks: top 1/3–high, middle 1/3–medium, and bottom 1/3–low. We included an additional bin to cover methylation-free transcripts. We only considered mRNA carrying at least one eTAM-seq evaluated site. Cumulative analysis was performed to probe the correlation between mRNA half-lives and their total methylation loads.

## Supplementary Material

SI

## Data Availability

All eTAM-seq data have been deposited to the NCBI Gene Expression Omnibus (GEO) and can be accessed through the GEO series accession number GSE201064^67^.
