# Supplementary material for: Transcriptome-wide profiling and quantification of N6-methyladenosine by enzyme-assisted adenosine deamination
Source: Nat Biotechnol. Author manuscript; Available in PMC 2023 Nov 5. (PMC10625715; doi:10.1038/s41587-022-01587-6)
Supplement: SI [file NIHMS1932016-supplement-SI.pdf]

Table of Contents

Supplementary Notes
Supplementary Note 1: Optimize assay conditions to maximize A-to-I conversion yields.
Supplementary Note 2: Estimate site accessibility using IVT controls.
Supplementary Note 3: Statistical model for m^{6}A detection and quantification from eTAM-seq data using an IVT control.
Supplementary Note 4: Compare three biological replicates of eTAM-seq (HeLa/IVT) using merged IVT controls.
Supplementary Note 5: Extract endogenous A-to-I editing sites and analyze the impact of endogenous A-to-I editing on eTAM-seq.
Supplementary Note 6: Statistical model for m^{6}A detection and quantification from eTAM-seq data using an FTO control.
Supplementary Note 7: Biological replicates of eTAM-seq (HeLa/FTO).
Supplementary Note 8: Biological replicates of eTAM-seq on mESCs.
Supplementary Note 9: Site-specific m^{6}A quantification by amplicon deep sequencing and eTAM-Sanger.

Supplementary Tables
Supplementary Table 1. TadA8.20-enabled A-to-I conversion in different sequence contexts as reported by non-methylated RNA probes.
Supplementary Table 2. Sequencing and processing parameters.
Supplementary Table 3. Selected m^{6}A sites for site-specific, deep sequencing-free methylation quantification.
Supplementary Table 4. Site-specific quantification of m^{6}A by eTAM-Sanger, eTAM-amplicon deep sequencing, and transcriptome-wide eTAM-seq.
Supplementary Table 5. Sequences of RNA oligos used in *in vitro* deamination assays.
Supplementary Table 6. Sequences of DNA oligos used to prepare dsDNA templates for *in vitro* transcription.
Supplementary Table 7. Sequences of spike-in RNA probes.
Supplementary Table 8. Sequences of DNA oligos used for reverse transcription of RNA probes.
Supplementary Table 9. Sequences of DNA oligos used to amplify target loci of HeLa mRNA and IVT RNA.
Supplementary Table 10. Sequences of DNA oligos for installation of Illumina adapters.

Supplementary Figures
Supplementary Figure 1. *In vitro* activity of TadA8.20.
Supplementary Figure 2. Optimization of assay conditions for TadA8.20-mediated global A deamination.

Table of Contents Continued

Supplementary Figure 3. Adenosine deamination activity of Tad8.20 prepared in different batches.
Supplementary Figure 4. Deamination of synthetic RNA probes carrying 0%, 25%, 50%, 75% and 100% m^{6}A in NNA/m^{6}ANN by TadA8.20.
Supplementary Figure 5. HeLa mRNA treated by TadA8.20.
Supplementary Figure 6. Preparation of an *in vitro* transcribed transcriptome (IVT) and HeLa rRNA treated by TadA8.20.
Supplementary Figure 7. Reproducibility of eTAM-seq (HeLa/IVT).
Supplementary Figure 8. m^{6}A sites captured by deep sequenced eTAM-seq (HeLa/IVT).
Supplementary Figure 9. Box plot (**a**) and scatter plot (**b**) showing persistent A signals detected in mRNA and IVT samples. Hits are binned by methylation levels reported by eTAM-seq (HeLa/IVT).
Supplementary Figure 10. Endogenous RNA editing poses minimal impact on eTAM-seq.
Supplementary Figure 11. Schematic of transcriptome-wide m^{6}A profiling by eTAM-seq assisted by an *N*^{6}-demethylated control transcriptome.
Supplementary Figure 12. Sequential demethylation and deamination of synthetic RNA probes by FTO and TadA8.20.
Supplementary Figure 13. Reproducibility of eTAM-seq with an FTO-treated control transcriptome.
Supplementary Figure 14. m^{6}A profiling in HeLa cells referenced to an FTO-treated transcriptome.
Supplementary Figure 15. Methylation levels at MALAT1_2515, 2577, 2611 and TPT1_687, 703 reported by eTAM-seq (HeLa/IVT), eTAM-seq (HeLa/FTO), and m^{6}A-SAC-seq.
Supplementary Figure 16. m^{6}A positions and fractions in 16 HeLa transcripts.
Supplementary Figure 17. Biological replicates for mESCs.
Supplementary Figure 18. m^{6}A profiling in mESCs.
Supplementary Figure 19. m^{6}A positions and fractions in selected regions of Oct4 and Rex1 (top) and full-length Nanog, Sox2, and Klf4 (bottom) in mESCs.
Supplementary Figure 20. m^{6}A profiling in ctrl and *Mettl3* KO mESCs.
Supplementary Figure 21. Simulated and observed distances of one m^{6}A site to its nearest neighbor.
Supplementary Figure 22. YTHDF2 regulates the stability of m^{6}A-modified mRNA in HeLa cells.
Supplementary Figure 23. Detection and quantification of m^{6}A by eTAM-seq.
Supplementary Figure 24. Amplification of m^{6}A-bearing transcripts from Tad8.20-treated mRNA and total RNA.

Supplementary Notes

Supplementary Note 1: optimize assay conditions to maximize A-to-I conversion yields

We hypothesized that efficiency of TadA8.20-mediated A-to-I conversion could be improved by 1) reducing RNA secondary structure and 2) increasing enzyme efficiency. We approached this hypothesis from two directions: assay temperature and pH. Higher temperature may denature RNA, thereby exposing previously inaccessible regions. Meanwhile, more hydroxide nucleophile, the key intermediate for the deamination reaction, may arise with higher pH and consequently lead to higher A-to-I conversion efficiency. We summarize our optimization campaign in **Supplementary Fig. 2**.

Conversion efficiency was improved by both higher temperature and higher pH, with temperature making a more significant contribution. Two confounding factors impact the assay as we raise the temperature: 1) resolution of RNA secondary structure and 2) impaired and eventually inactivated enzyme. We noticed that TadA8.20 stays robust up to 44°C and loses activity at 55°C quickly. Meanwhile, when the assay was carried out at 53°C, we observed significantly elevated G signals in regions resistant to A-to-I conversion at 37°C, a fact that we attribute to resolved RNA secondary structure. Higher pH also led to increased A-to-I conversion, albeit to a lesser extent. As RNA is less stable at higher pH, we decide to stick to close-to-neutral pH (pH 7.5). The final choice of assay condition is: 1 h incubation at 53°C (for hard-to-convert regions) followed by 2 h treatment at 44°C with freshly supplemented enzyme (for optimal global A-to-I conversion).

Supplementary Note 2: estimate site accessibility using IVT controls

We propose that accessibility of a given A site stays the same in HeLa and IVT samples because the sequence context, which prompts formation of secondary structures, is consistent in both samples. In IVT samples, lower deamination rates can only arise if a site is partially blocked, whereas in mRNA samples, both the presence of m^6A and compromised accessibility contribute to lower deamination rates. We therefore propose the following relationships and equations:

G%(mRNA) ≤ G%(IVT);
Presence of m^6A = (G%(mRNA) < G%(IVT) with statistical significance);
Apparent methylation level = 1 – G%(mRNA);
Accessibility of an A site = G%(IVT);
True methylation level = 1– G%(mRNA)/G%(IVT).

The accessibility parameter calculated based on the A and G counts observed in IVT samples is applied to the apparent methylation signals detected in mRNA and outputs the true methylation levels. Our statistical model is detailed in **Supplementary Note 3**, but we provide the following simplified worksheet to showcase our workflow.

Note that the methylation level estimation can be more error-prone at sites of low accessibility as only accessible A/m^6A produces eTAM-seq signals. We define exposed methylation level as follows: exposed methylation level = true methylation level * site accessibility. Exposed methylation levels are only used to define the cutoff for high-confidence m^6A sites, with the intention of excluding hits of extremely low methylation and accessibility. **"Methylation levels" in our study always refers to true methylation levels**.

G% (mRNA)	G% (IVT)	Apparent methylation level	Site accessibility	**True methylation level**	Exposed methylation level (only for cutoff)
1	1	0	1	**0**	0
0.8	1	0.2	1	**0.2**	0.2
0.5	1	0.5	1	**0.5**	0.5
0.2	1	0.8	1	**0.8**	0.8
0	1	1	1	**1**	1
0.8	0.8	0.2	0.8	**0**	0
0.5	0.8	0.5	0.8	**0.375**	0.3
0.2	0.8	0.8	0.8	**0.75**	0.6
0	0.8	1	0.8	**1**	0.8
0.5	0.5	0.5	0.5	**0**	0
0.2	0.5	0.8	0.5	**0.6**	0.3
0	0.5	1	0.5	**1**	0.5

Supplementary Note 3: statistical model for m^{6}A detection and quantification from eTAM-seq data using an IVT control

Statistical models are provided at the end of the file.

Supplementary Note 4: compare three biological replicates of eTAM-seq (HeLa/IVT) using merged IVT controls

As sequence context, the primary determinant of site accessibility, remains the same in RNA samples collected from the same genetic background, we envision that *in vitro* transcribed RNA should behave consistently across biological replicates for site accessibility estimation. To this end, we merged HeLa-IVT-1, -2, and -3 for a more comprehensive control transcriptome. We processed three biological replicates of eTAM-seq (HeLa-1-3) using this merged control transcriptome and called out m^{6}A sites with exposed methylation levels ≥ 10%. As the merged IVT control covers significantly more sites, we detected a lot more m^{6}A sites in each biological sample: 42,135, 42,029, and 42,119 for HeLa-1, HeLa-2, and HeLa-3, respective, 34,321 of which persist across three replicates (**Supplementary Fig. 7**). Only 3,330 (8%), 3,161 (8%), and 3,481 (8%) sites are unique to HeLa-1, HeLa-2, and HeLa-3, respectively. The majority of hits, 36,474 for HeLa-1 (87%), 36,435 for HeLa-2 (87%), and 36,129 for HeLa-3 (86%), emerge in DRACH motifs.

When we analyzed eTAM-seq (HeLa/IVT-1), eTAM-seq (HeLa/IVT-2), and eTAM-seq (HeLa/IVT-3) separately, we found that eTAM-seq (HeLa/IVT-1) and eTAM-seq (HeLa/IVT-2) had more unique hits than eTAM-seq (HeLa/IVT-3) (**Fig. 2a**), likely due to deeper sampling of HeLa-IVT-1 and HeLa-IVT-2. We therefore hypothesize that some of these unique hits arise due to heterogeneity in sampling. To test this hypothesis, we overlapped deep sequenced eTAM-seq (HeLa/IVT-1) with eTAM-seq (HeLa/IVT-2) and eTAM-seq (HeLa/IVT-3). In this case, only 961 (4.9%) and 546 (3.6%) hits are unique to eTAM-seq (HeLa/IVT-2) and eTAM-seq (HeLa/IVT-3), respectively. Collectively, we believe a portion of the replicate-unique hits can be attributed to heterogeneity in sampling, rather than false positive detection by eTAM-seq.

Supplementary Note 5: extract endogenous A-to-I editing sites and analyze the impact of endogenous A-to-I editing on eTAM-seq

RNA editing is a natural process widely occurring in eukaryotes. The most common type of RNA editing is A-to-I conversion mediated by adenosine deaminases acting on RNA (ADARs)[1]. As endogenous A-to-I editing shifts the distribution of A and G in RNA-seq at genomic A sites, we set out to analyze its impact on eTAM-seq. We used RNA-seq data of two HeLa biological replicates (ENCSR000CPR) from the ENCODE project website (https://www.encodeproject.org/) for RNA-editing site identification. We combined two samples for mutation calling and applied stringent quality control and multiple filters to obtain high-confidence RNA-editing sites (see **Supplementary Fig. 10a** for detailed processing steps). We identified in total 29,052 RNA-editing sites, with 77.3% (22,472) being A-to-I editing. Of the detected RNA-editing sites, 43% located in introns, 30% in 3' UTRs, 13% in IGRs, 10% in ncRNA, 3% in CDS, and 1% in 5' UTRs, with 79.7% found in Alu elements (**Supplementary Fig. 10b**). Overall, the genomic distribution of RNA-editing sites is distinct from that of m^6A sites detected by eTAM-seq. Only three sites overlap between RNA-editing sites and eTAM-seq hits (**Supplementary Fig. 10c**). We therefore conclude that the fidelity of eTAM-seq is not compromised by endogenous RNA-editing events.

Supplementary Note 6: statistical model for m^6A detection and quantification from eTAM-seq data using an FTO control

Statistical models are provided at the end of the file.

Supplementary Note 7: biological replicates of eTAM-seq (HeLa/FTO)

We treated three biological samples of HeLa mRNA by FTO (**Supplementary Fig. 13**). Both untreated and FTO-treated mRNA samples were further processed by TadA8.20, resulting in three biological replicates – eTAM-seq (HeLa/FTO-1), eTAM-seq (HeLa/FTO-2), and eTAM-seq (HeLa/FTO-3). We processed three replicates separately and called out m^6A sites with exposed methylation levels ≥ 10%. We identified 21,728, 20,337, and 15,789 m^6A sites from individual replicates, 19,646 (90%), 18,542 (91%), and 14,407 (91%) of which were in DRACH motifs. Of the identified hits, 13,147 (61-83%) are common to three replicates and show highly consistent methylation levels (Pearson's r = 0.96-0.97). Only 3,699, 2,492, and 950 sites are unique to eTAM-seq (HeLa/FTO-1), eTAM-seq (HeLa/FTO-2), and eTAM-seq (HeLa/FTO-3), respectively.

In addition to comparing methylation levels reported by different biological replicates, we also performed correlative analysis on methylation levels estimated using different control transcriptomes. Cross comparison of 6 samples (HeLa/IVT-1, HeLa/IVT-2, HeLa/IVT-3, HeLa/FTO-1, HeLa/FTO-2, and HeLa/FTO-3) shows that the estimated methylation levels in the same biological sample are almost identical when referenced to different controls (Pearson's r = 0.99-1.00). Moreover, methylation levels in different biological samples estimated using different controls are highly correlated (Pearson's r > 0.96), confirming the consistency of the m^6A landscape in HeLa cells and the high reproducibility of eTAM-seq.

Supplementary Note 8: biological replicates of eTAM-seq on mESCs

We harvested two batches of mESCs and prepared IVT and FTO-treated samples separately (**Supplementary Fig. 17a** and **17b**). Two biological replicates were obtained accordingly with both IVT and FTO controls: mESC/IVT-1, mESC/IVT-2; mESC/FTO-1, mESC/FTO-2. We detected 24,676 and 26,756 m^6A sites in eTAM-seq (mESC/IVT-1) and eTAM-seq (mESC/IVT-2), respectively, 20,727 of which were shared (**Supplementary Fig. 17c**). Many replicate-unique hits can be attributed to sequencing depth that inevitably varies among samples. Similar to what was observed for HeLa samples, the majority of hits were detected in DRACH

motifs (21,524, 87% for mESC/IVT-1; 21508, 80% for mESC/IVT-2, **Supplementary Fig. 17d**). The methylation levels reported by two biological replicates are highly consistent (Pearson's r = 0.95, **Supplementary Fig. 17e**), suggesting that 1) IVT functions consistently across replicates for accessibility estimation; and 2) the m^6A deposition machinery favors a similar category of target sites in the same cell type, at least at the bulk level.

We next merged two IVT samples and obtained a control transcriptome covering more A sites. With this control transcriptome, we detected many more m^6A sites: 46,377 in mESC-1, 39,824, 86% in DRACH; 43,170 in mESC-2, 34,035, 79% in DRACH. 35,080 of these hits overlap (76% of mESC-1, 81% of mESC-2, **Supplementary Fig. 17f**).

We processed eTAM-seq (mESC/FTO-1) and eTAM-seq (mESC/FTO-2) separately and identified 28,788 (26,122 in DRACH, 91%) and 18,577 (16,210 in DRACH, 87%) m^6A sites, respectively. Similar to what was observed in eTAM-seq (mESC/IVT), the majority of these sites overlap across two replicates (16,076, 56% of mESC/FTO-1 and 87% of mESC/FTO-2, **Supplementary Fig. 17g** and **17h**) with highly consistent methylation levels (Pearson's r = 0.95, **Supplementary Fig. 17i**). mESC samples also behave consistently when different controls, IVT or FTO, are applied. To simplify the results, we present the comparison between eTAM-seq (mESC/IVT-1) and eTAM-seq (mESC/FTO-1) in the main text. Very similar results were obtained when we compared eTAM-seq (mESC/IVT-2) and eTAM-seq (mESC/FTO-2), or cross compared eTAM-seq (mESC/IVT-1) and eTAM-seq (mESC/FTO-2); and eTAM-seq (mESC/IVT-2) and eTAM-seq (mESC/FTO-1) (**Supplementary Fig. 17j**). Collectively, we carried out two biological replicates of eTAM-seq for mESCs, confirming the consistency of m^6A deposition in mESCs and the robustness of eTAM-seq.

Supplementary Note 9

Site-specific m^6A quantification by amplicon deep sequencing. Raw next-generation sequencing data were merged by Pear v0.9.8. before mapping to the target sequences using bwa-mem2 v2.2.1. Mapped reads were sorted and indexed using samtools v1.14. Bases mapped to individual A sites were counted using pysamstats v1.1.1. A and G counts were extracted for individual A sites covered by R2 using re.finditer. Methylation levels are reported as apparent A fractions.

Site-specific m^6A quantification by eTAM-Sanger. The A and G fractions observed at all A sites were quantified by EditR[2]. Methylation levels are reported as apparent A fractions.

Supplementary Tables

Supplementary Table 1 | TadA8.20-enabled A-to-I conversion in different sequence contexts as reported by non-methylated RNA probes. Also provided as a separate EXCEL worksheet.

**ID**	**Motif**<br>**Post-conversion**<br>(pre-conversion)	**Type**	**Probes**<br><br>**A-to-G count**	**Total count**	**conversion rate**
spikein-1	**GGACU**<br>(AAACU/AGACU/GAACU/GGACU)	**DRACH**	576	582	98.97
spikein-1	**UGACU**<br>(UAACU/UGACU)	**DRACH**	270	270	100
spikein-1	**GGACC**<br>(AAACC/AGACC/GAACC/GGACC)	**DRACH**	201	204	98.53
spikein-1	**UGACC**<br>(UAACC/UGACC)	**DRACH**	81	81	100
spikein-1	GGAGG	nonDRACH	3590	3688	97.34
spikein-1	GGAGU	nonDRACH	2339	2380	98.28
spikein-1	GGAUG	nonDRACH	2317	2347	98.72
spikein-1	GUAGG	nonDRACH	2158	2179	99.04
spikein-1	UGAGG	nonDRACH	1825	1854	98.44
spikein-1	GGAUU	nonDRACH	1489	1501	99.2
spikein-1	GUAGU	nonDRACH	1435	1445	99.31
spikein-1	GUAUG	nonDRACH	1300	1317	98.71
spikein-1	GGAGC	nonDRACH	1230	1291	95.27
spikein-1	UGAGU	nonDRACH	1150	1164	98.8
spikein-1	GGACG	nonDRACH	1139	1153	98.79
spikein-1	UGAUG	nonDRACH	1119	1131	98.94
spikein-1	UUAGG	nonDRACH	977	985	99.19
spikein-1	GUAUU	nonDRACH	806	811	99.38
spikein-1	GCAGG	nonDRACH	805	824	97.69
spikein-1	UGAUU	nonDRACH	730	736	99.18
spikein-1	GUAGC	nonDRACH	716	724	98.9
spikein-1	GUACG	nonDRACH	695	701	99.14
spikein-1	CGAGG	nonDRACH	690	710	97.18
spikein-1	UUAGU	nonDRACH	670	673	99.55
spikein-1	GGAUC	nonDRACH	604	615	98.21
spikein-1	UUAUG	nonDRACH	575	579	99.31
spikein-1	UGAGC	nonDRACH	574	582	98.63
spikein-1	GCAUG	nonDRACH	573	581	98.62
spikein-1	UGACG	nonDRACH	547	553	98.92
spikein-1	GCAGU	nonDRACH	541	549	98.54

spikein-1	CGAGU	nonDRACH	451	459	98.26
spikein-1	CGAUG	nonDRACH	428	432	99.07
spikein-1	GCAUU	nonDRACH	388	390	99.49
spikein-1	GUAUC	nonDRACH	373	375	99.47
spikein-1	UUAUU	nonDRACH	352	357	98.6
spikein-1	UCAGG	nonDRACH	341	346	98.55
spikein-1	UUAGC	nonDRACH	338	340	99.41
spikein-1	GUACU	nonDRACH	315	318	99.06
spikein-1	UUACG	nonDRACH	294	296	99.32
spikein-1	CUAGG	nonDRACH	290	293	98.98
spikein-1	CGAUU	nonDRACH	285	288	98.96
spikein-1	UGAUC	nonDRACH	285	288	98.96
spikein-1	GCAGC	nonDRACH	268	271	98.89
spikein-1	UCAGU	nonDRACH	250	253	98.81
spikein-1	CGAGC	nonDRACH	239	248	96.37
spikein-1	CUAGU	nonDRACH	209	214	97.66
spikein-1	CGACG	nonDRACH	200	203	98.52
spikein-1	UCAUG	nonDRACH	197	201	98.01
spikein-1	GCACG	nonDRACH	196	197	99.49
spikein-1	CUAUG	nonDRACH	174	174	100
spikein-1	GCAUC	nonDRACH	162	163	99.39
spikein-1	UCAUU	nonDRACH	159	162	98.15
spikein-1	UUAUC	nonDRACH	150	155	96.77
spikein-1	UUACU	nonDRACH	149	149	100
spikein-1	CUAUU	nonDRACH	141	142	99.3
spikein-1	GCACU	nonDRACH	105	106	99.06
spikein-1	CGACU	nonDRACH	104	105	99.05
spikein-1	UCAGC	nonDRACH	99	100	99
spikein-1	CUAGC	nonDRACH	98	98	100
spikein-1	CUACG	nonDRACH	97	98	98.98
spikein-1	CGAUC	nonDRACH	96	97	98.97
spikein-1	CCAGG	nonDRACH	94	102	92.16
spikein-1	GUACC	nonDRACH	85	87	97.7
spikein-1	UCACG	nonDRACH	76	79	96.2
spikein-1	CCAUG	nonDRACH	68	69	98.55
spikein-1	UCAUC	nonDRACH	67	67	100
spikein-1	CCAGU	nonDRACH	63	65	96.92
spikein-1	UCACU	nonDRACH	56	59	94.92
spikein-1	CCAUU	nonDRACH	47	47	100
spikein-1	CUAUC	nonDRACH	47	47	100

spikein-1	UUACC	nonDRACH	42	42	100
spikein-1	CUACU	nonDRACH	39	39	100
spikein-1	GCACC	nonDRACH	36	36	100
spikein-1	CCACG	nonDRACH	33	36	91.67
spikein-1	CCAGC	nonDRACH	29	32	90.62
spikein-1	CGACC	nonDRACH	29	29	100
spikein-1	UCACC	nonDRACH	15	15	100
spikein-1	CUACC	nonDRACH	14	14	100
spikein-1	CCAUC	nonDRACH	12	12	100
spikein-1	CCACU	nonDRACH	10	10	100
spikein-1	CCACC	nonDRACH	4	5	80

Supplementary Table 2 | The sequencing and processing statistics of eTAM-seq libraries. Also provided as a separate EXCEL worksheet.

Cell	Genotype	treatment	Replicate	Unique reads (rRNA mapping)	Multiple reads (rRNA mapping)	Unique reads (genome mapping)	Multiple reads (genome mapping)	Non-duplicated reads	Reads with >= 50% A2G conversion	Conversion rate (spikein-1)
HeLa	Wild type	mRNA	rep1	525931	2	42293862	3859856	25236436	23913512	98.59%
HeLa	Wild type	mRNA	rep1 (deep)	1225064	8	99690950	8856860	57773534	55086488	98.63%
HeLa	Wild type	mRNA	rep2	722750	4	39551201	3710685	25619897	23579662	98.49%
HeLa	Wild type	mRNA	rep3	856181	19	44356265	4320917	26794896	24688455	98.46%
HeLa	Wild type	FTO+	rep1	548508	5	68386283	4947367	22108633	20971371	98.60%
HeLa	Wild type	FTO+	rep1 (deep)	1053919	7	131470574	9381265	31809525	30396010	98.65%
HeLa	Wild type	FTO+	rep2	793062	4	62013945	4402918	18283777	17370949	98.53%
HeLa	Wild type	FTO+	rep3	603217	5	47751235	3660275	11670026	11000658	98.46%
HeLa	Wild type	IVT	rep1	257725	1	45949687	5386091	28872217	26518648	98.35%
HeLa	Wild type	IVT	rep1 (deep)	617575	7	116496666	13347101	67824846	63114294	98.36%
HeLa	Wild type	IVT	rep2	576437	6	48204770	7864292	30682480	28233147	98.49%
HeLa	Wild type	IVT	rep3	241426	4	23379930	4047620	14871021	13712100	98.45%
mESC	*Mettl3* cko	mRNA	rep1	512028	5	45749545	6940161	21422529	19648088	97.94%
mESC	*Mettl3* cko	FTO+	rep1	772614	2	81475781	11536575	24282747	23229637	98.63%
mESC	*Mettl3* cko ctrl	mRNA	rep1	787989	11	88818103	12812937	22393782	19831784	97.62%
mESC	*Mettl3* cko ctrl	FTO+	rep1	909142	5	97897894	14473199	22044716	20771286	98.32%
mESC	Wild type	mRNA	rep1	854554	2	70180331	13356193	34432294	32578878	98.54%
mESC	Wild type	mRNA	rep1 (deep)	1509709	4	126852810	23558808	53444077	50985475	98.55%
mESC	Wild type	mRNA	rep2	1783460	24	43901877	7906504	24488630	21800245	98.15%
mESC	Wild type	FTO+	rep1	835413	1	73629827	12949575	23638637	22569835	98.58%
mESC	Wild type	FTO+	rep1 (deep)	1483035	1	132019073	22788562	33544932	32187127	98.61%
mESC	Wild type	FTO+	rep2	1550654	17	44528241	7283968	11387552	10598421	98.42%
mESC	Wild type	IVT	rep1	469506	16	43081878	13796185	23188839	20156185	98.39%
mESC	Wild type	IVT	rep1 (deep)	895884	21	83531678	26277099	39188224	34825079	98.45%
mESC	Wild type	IVT	rep2	1331254	20	62735458	18131279	34535483	31701923	98.36%

Supplementary Table 3 | Selected m^6A sites for site-specific, deep sequencing-free methylation quantification.

mRNA	Genomic position	RefSeq ID	Position of m^6A site
MALAT1	chr11_65500338_+	NR_002819.4	2601 (2577 in SCARLET paper[3])
ACTB	chr7_5527533_-	NM_001101.5	1427
CAND1	chr12_67312820_+	NM_018448.5	4043
CIAO1	chr2_96271368_+	NM_004804.3	1163
CLCN3	chr4_169720370_+	NM_001829.4	3332
EIF2A	chr3_150572124_+	NM_032025.5	994
HDAC2	chr6_113940950_-	NM_001527.4	1815
HOXB7	chr17_48607568_-	NM_004502.4	1027
MYC	chr8_127741071_+	NM_002467.6	1841
OGT	chrX_71574128_+	NM_181672.3	3671
SLC7A5	chr16_87831096_-	NM_003486.7	3483
TPX2	chr20_31801189_+	NM_012112.5	2862
ZBED5	chr11_10853818_-	NM_001143667.2	1575
GRWD1	chr19_48453148_+	NM_031485.4	1487
H2AFX	chr11_119094135_-	NM_002105.3	1331
JUNB	chr19_12792837_+	NM_002229.3	1352
MRPL36	chr5_1798728_-	NM_032479.4	266
PPIB	chr15_64155887_-	NM_000942.5	823
ILF3	chr19_10689503_+	NM_012218.4	3108

Supplementary Table 4 | Site-specific quantification of m^6A by eTAM-Sanger, eTAM-amplicon deep sequencing, and transcriptome-wide eTAM-seq.

mRNA	**eTAM-Sanger**	**eTAM-amplicon deep sequencing**	**eTAM-seq**
MALAT1	88	83.58678553	81.0748
ACTB	76	69.10577017	71.1198
CAND1	86	96.84842421	94.4339
CIAO1	81	94.38519448	92.2028
CLCN3	96	97.88333744	97.5899
EIF2A	92	92.32268122	93.2209
HDAC2	79	71.3243147	66.0735
HOXB7	82	85.44073429	83.0128
MYC	73	71.066058	66.3526
OGT	70	67.15176715	59.9254
SLC7A5	98	96.60040334	94.2525
TPX2	87	85.95203133	81.3943
ZBED5	91	83.67654445	71.3695
GRWD1	89	96.78840693	95.1742
H2AFX	82	84.86422318	82.6652
JUNB	96	98.02398244	100
PPIB	67	78.5814466	74.1578
ILF3	91	90.58590278	87.492

Supplementary Table 5 | Sequences of RNA oligos used in *in vitro* deamination assays.

RNA	Sequences
E. coli tRNA(Arg2, CGT)	GCAUCCGUAGCUCAGCUGGAUAGAGUACUCGGCUACGAACCGAGCGGUCGGAGGUUCGAAUCCUCCCGGAUGCACCA
RNA#1	GGCUUCGUUGUUGUGCUGGUUUGUGUUCUCUUGUUCGUUCUGGUUCGUUCGA(m^6A)UCUCGGUUCGUUUGCUGGCUUCUGUUCCGUUU
RNA#2	GGCUUCGUUGUUGUGCUGGUUUGUGUUCUCUUCUUCGUUCUGGUUCGUUGGA(m^6A)CUUCGGUUCGUUUGCUGGCUUCUGUUCCGUUU
RNA#3	GGCUUCGUUGUUGUGCUGGUUGCUGUCUGCCAGGUGAUCGCUGAUGUACUGACAAGCCUCGCGUACCCGAUUAUCCAUCGGUGGAUGGAGCGACUCGUUAAUCGCUUCCAUGCGCCGCAGUAACAAUUGCUCAAGCAGAUUUAUCGCCAGCAGCUCCGAAUAGCGCCCUCUUCUGUUCCGUUU

Supplementary Table 6 | Sequences of DNA oligos used to prepare dsDNA templates for *in vitro* transcription.

tRNA_Fwd_WT1597	TAATACGACTCACTATAGCATCCGTAGCTCAGCTGGATAGAGTACTCGGCTACGAACCG
tRNA_Rev_WT1598	TGGTGCATCCGGGAGGATTCGAACCTCCGACCGCTCGGTTCGTAGCCGAG
RNA#1_Fwd_YX346	TAATACGACTCACTATAGGCTTCGTTGTTGTGCTGGTTTGTGTTCTCTTGTTCGTTCTGG
RNA#1_Rev_YX347	AAACGGAACAGAAGCCAGCAAACGAACCGAGATCGAACGAACCAGAACGAACAAGAGAAC
RNA#2_Fwd_YX351	TAATACGACTCACTATAGGCTTCGTTGTTGTGCTGGTTTGTGTTCTCTTCTTCGTTCTGG
RNA#2_Rev_YX352	AAACGGAACAGAAGCCAGCAAACGAACCGAAGTCCAACGAACCAGAACGAAGAAGAGAAC
RNA#3_Fwd_YX360	TAATACGACTCACTATAGGCTTCGTTGTTGTGCTGGTTGCTGTCTGCCAGGTGATCG
RNA#3_Rev_YX361	AAACGGAACAGAAGAGGGCGCTATTCGGAGCTGC

Supplementary Table 7 | Sequences of spike-in RNA probes.

Probe 1	rUrArUrCrUrGrUrCrUrCrGrArCrGrUrNrNrArNrNrGrGrCrCrUrUrUrGrCrArArCrUrArGrArArUrUrArCrArCrCrArUrArArUrUrGrCrU
Probe 2	rUrArUrCrUrGrUrCrUrCrGrArCrGrUrNrNrArNrNrGrGrCrArUrUrCrArArGrCrCrUrArGrArArUrUrArCrArCrCrArUrArArUrUrGrCrU
Probe 3	rUrArUrCrUrGrUrCrUrCrGrArCrGrUrNrNrArNrNrGrGrCrGrArGrGrUrGrArUrCrUrArGrArArUrUrArCrArCrCrArUrArArUrUrGrCrU
Probe 4	rUrArUrCrUrGrUrCrUrCrGrArCrGrUrNrNrArNrNrGrGrCrUrUrCrArArCrArArCrUrArGrArArUrUrArCrArCrCrArUrArArUrUrGrCrU
Probe 5	rUrArUrCrUrGrUrCrUrCrGrArCrGrUrNrN/iN6Me-rA/rNrNrGrGrCrArUrUrCrArArGrCrCrUrArGrArArUrUrArCrArCrCrArUrArArUrUrGrCrU
Probe 6	rUrArUrCrUrGrUrCrUrCrGrArCrGrUrNrN/iN6Me-rA/rNrNrGrGrCrGrArGrGrUrGrArUrCrUrArGrArArUrUrArCrArCrCrArUrArArUrUrGrCrU
Probe 7	rUrArUrCrUrGrUrCrUrCrGrArCrGrUrNrN/iN6Me-rA/rNrNrGrGrCrUrUrCrArArCrArArCrUrArGrArArUrUrArCrArCrCrArUrArArUrUrGrCrU
Probe 8	rUrArUrCrUrGrUrCrUrCrGrArCrGrUrNrN/iN6Me-rA/rNrNrGrGrCrGrArUrGrGrUrUrUrCrUrArGrArArUrUrArCrArCrCrArUrArArUrUrGrCrU

Supplementary Table 8 | Sequences of DNA oligos used for reverse transcription of RNA probes.

tRNA_RT_YX098	TCCGAATAGCGCCCTTCCCCTTGCCCGGCGTTAATGATTTGCCCAAATGGTGCATCCG
RNA#1_RT_YX348	GCACATCCATCAGGGAGCCCCTAAGTCGAACCATGTGCTCAAACGGAACAGAAG
RNA#2_RT_YX348	GCACATCCATCAGGGAGCCCCTAAGTCGAACCATGTGCTCAAACGGAACAGAAG
RNA#3_RT_YX348	GCACATCCATCAGGGAGCCCCTAAGTCGAACCATGTGCTCAAACGGAACAGAAG

Supplementary Table 9 | Sequences of DNA oligos used to amplify target loci of HeLa mRNA and IVT RNA.

EIF2A_Fwd_YX989	ACACTCTTTCCCTACACGACGCTCTTCCGATCTNNNNTGCGGCCTGCTGTGGC
EIF2A_Rev_YX990	GACTGGAGTTCAGACGTGTGCTCTTCCGATCTCAAACCCAGCCAACACCAAC
ZBED5_Fwd_YX993	ACACTCTTTCCCTACACGACGCTCTTCCGATCTNNNNCGGTGGTCRCTGCCTGTT
ZBED5_Rev_YX994	GACTGGAGTTCAGACGTGTGCTCTTCCGATCTGGCACCACCCCAACYGCCAG
CAND1_Fwd_YX_997	ACACTCTTTCCCTACACGACGCTCTTCCGATCTNNNNGTCTGCTGGCTTGGGGTC
CAND1_Rev_YX_998	GACTGGAGTTCAGACGTGTGCTCTTCCGATCTCGGCGAACAAACACCCAAC
MALAT1_Fwd_YX1001	ACACTCTTTCCCTACACGACGCTCTTCCGATCTNNNNGCCGGCTTGGTGTTTTTGC
MALAT1_Rev_YX1002	GACTGGAGTTCAGACGTGTGCTCTTCCGATCTGGAYYCAAAAAACAACCCCAACC
ACTB_Fwd_YX1005	ACACTCTTTCCCTACACGACGCTCTTCCGATCTNNNNCRRGGTTCGCGGTGTGG
ACTB_Rev_YX1006	GACTGGAGTTCAGACGTGTGCTCTTCCGATCTCACCAAAAAAACAACAACGCGC
MYC_Fwd_YX1009	ACACTCTTTCCCTACACGACGCTCTTCCGATCTNNNNCGGCCTTGGCTGGGTC
MYC_Rev_YX1010	GACTGGAGTTCAGACGTGTGCTCTTCCGATCTCACACCACGGCCAAACCC
SLC7A5_Fwd_YX1025	ACACTCTTTCCCTACACGACGCTCTTCCGATCTNNNNGGCCTGTTCTCTTGGCTGC
SLC7A5_Rev_YX1026	GACTGGAGTTCAGACGTGTGCTCTTCCGATCTGGCAGGYGGGGCACCAG
CIAO1_Fwd_YX1032	ACACTCTTTCCCTACACGACGCTCTTCCGATCTNNNNCCTCTGGGCTGCCTCG
CIAO1_Rev_YX1033	GACTGGAGTTCAGACGTGTGCTCTTCCGATCTCCCGGGGAGYCACCAC
CLCN3_Fwd_YX1039	ACACTCTTTCCCTACACGACGCTCTTCCGATCTNNNNCTGTTGCTCCGGCGTTG
CLCN3_Rev_YX1040	GACTGGAGTTCAGACGTGTGCTCTTCCGATCTCCYCCAGAAACAGGGACYGAC
TPX2_Fwd_YX1013	ACACTCTTTCCCTACACGACGCTCTTCCGATCTNNNNCTTTGTCGTTGGGCRTGGG
TPX2_Rev_YX1014	GACTGGAGTTCAGACGTGTGCTCTTCCGATCTGGCACGGGCAGGCAA
ILF3_Fwd_YX1059	ACACTCTTTCCCTACACGACGCTCTTCCGATCTNNNNGCCCRTGTTTGGCCGTG

ILF3_Rev_YX1060	GACTGGAGTTCAGACGTGTGCTCTTCCGATCTACCAACAAACCACCGAAGACA
HDAC2_Fwd_YX1061	ACACTCTTTCCCTACACGACGCTCTTCCGATCTNNNNCGTTTTGTGCTGCTTTGGC
HDAC2_Rev_YX1062	GACTGGAGTTCAGACGTGTGCTCTTCCGATCTCGAAAAAGCCACCCGAAAACAAA
OGT_Fwd_YX1063	ACACTCTTTCCCTACACGACGCTCTTCCGATCTNNNNGGCCGCTTTGTGGTTCC
OGT_Rev_YX1064	GACTGGAGTTCAGACGTGTGCTCTTCCGATCTAAAGAAAAAAAAACCCAGAAGACCGA
HOXB7_Fwd_YX1070	ACACTCTTTCCCTACACGACGCTCTTCCGATCTNNNNGTTTGGTTTTTTTGTGCGGTGTG
HOXB7_Rev_YX1071	GACTGGAGTTCAGACGTGTGCTCTTCCGATCTCAGAAAGCCACAGAACAGGCAG
JUNB_Fwd_YX1035	ACACTCTTTCCCTACACGACGCTCTTCCGATCTNNNNGRRCGTCCCCTGCCCCTT
JUNB_Rev_YX1036	GACTGGAGTTCAGACGTGTGCTCTTCCGATCTCAGCCGYCCAAGCGAGG
GRWD1_Fwd_YX1067	ACACTCTTTCCCTACACGACGCTCTTCCGATCTNNNNCCTGTGGGCTGCCGT
GRWD1_Rev_YX1068	GACTGGAGTTCAGACGTGTGCTCTTCCGATCTCCCAGAGAACAACACGCCAA
H2AFX_Fwd_YX1028	ACACTCTTTCCCTACACGACGCTCTTCCGATCTNNNNTTGGCTTCRCGGCTGGCT
H2AFX_Rev_YX1029	GACTGGAGTTCAGACGTGTGCTCTTCCGATCTGCYYCCGCGAAAACGACCC
PPIB_Fwd_YX1054	ACACTCTTTCCCTACACGACGCTCTTCCGATCTNNNNCTCGCGTTCCGCGGGC
PPIB_Rev_YX1055	GACTGGAGTTCAGACGTGTGCTCTTCCGATCTCAGGAGCCCGCCACAA

Supplementary Table 10 | Sequences of DNA oligos for installation of Illumina adapters.

tRNA_Fwd_YX115	ACACTCTTTCCCTACACGACGCTCTTCCGATCTNNNNGCATCCGTAGCTCAGCTGG
tRNA_Rev_YX117	GACTGGAGTTCAGACGTGTGCTCTTCCGATCTTCCGAATAGCGCCCTTCC
RNA#1_Fwd_YX349	ACACTCTTTCCCTACACGACGCTCTTCCGATCTNNNNGGCTTCGTTGTTGTGCTGG
RNA#1_Rev_YX132	GACTGGAGTTCAGACGTGTGCTCTTCCGATCTGCACATCCATCAGGGAGCC
RNA#2_Fwd_YX349	ACACTCTTTCCCTACACGACGCTCTTCCGATCTNNNNGGCTTCGTTGTTGTGCTGG
RNA#2_Rev_YX132	GACTGGAGTTCAGACGTGTGCTCTTCCGATCTGCACATCCATCAGGGAGCC
RNA#3_Fwd_YX349	ACACTCTTTCCCTACACGACGCTCTTCCGATCTNNNNGGCTTCGTTGTTGTGCTGG
RNA#3_Rev_YX132	GACTGGAGTTCAGACGTGTGCTCTTCCGATCTGCACATCCATCAGGGAGCC

Supplementary Figures

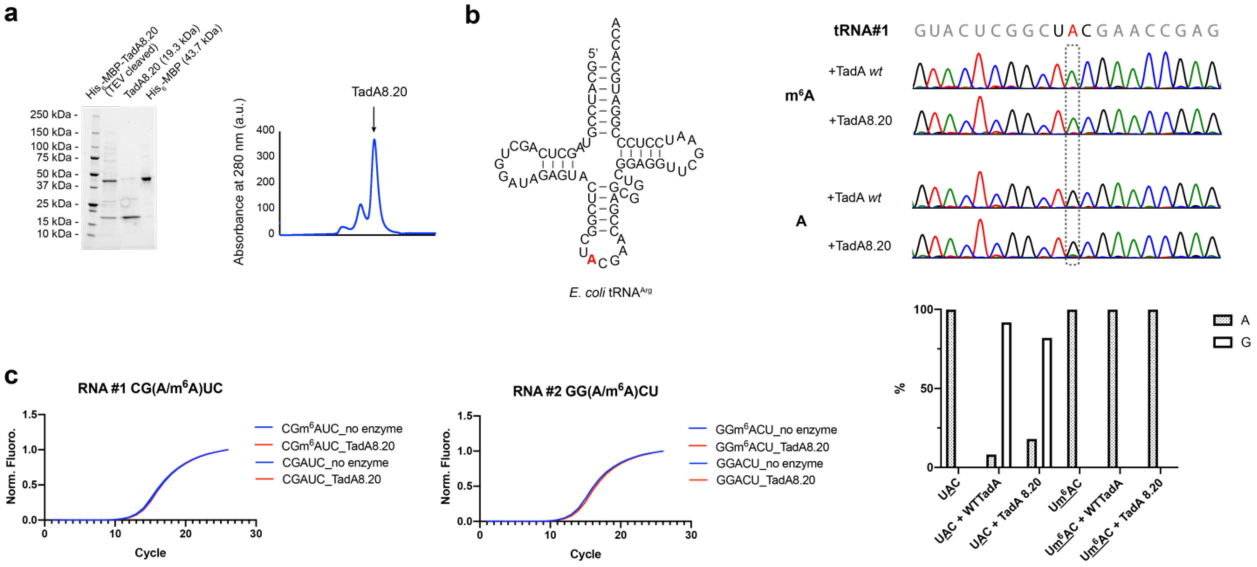

Supplementary Figure 1 | *In vitro* activity of TadA8.20. a. Sodium dodecyl-sulfate polyacrylamide gel electrophoresis (SDS-PAGE, left) and size exclusion chromatography (right) analyses of TadA8.20. Experiments were repeated independently for three times with similar results. **b**. Unmethylated and methylated *E. coli* $tRNA^{Arg}$(ACG) treated with wildtype TadA and TadA8.20. $tRNA^{Arg}$(ACG) is the natural substrate of TadA, i.e., likely the RNA sequence best accepted by TadA. The fact that a TadA enzyme accepts this substrate does not necessarily translate into function on other RNA, especially RNA of user-defined sequences. However, we believe $tRNA^{Arg}$(m^6ACG) is the best substrate to probe whether TadA and TadA derivatives accept m^6A. Unmethylated and methylated tRNA were prepared through *in vitro* transcription using ATP and N^6-methyl-ATP as starting materials, respectively. Treated RNA was reverse transcribed, amplified by PCR, and subjected to Sanger sequencing (top) and next-generation sequencing (bottom). **c**. RT-qPCR analysis of RNA sequences pre- and post-TadA8.20 treatment.

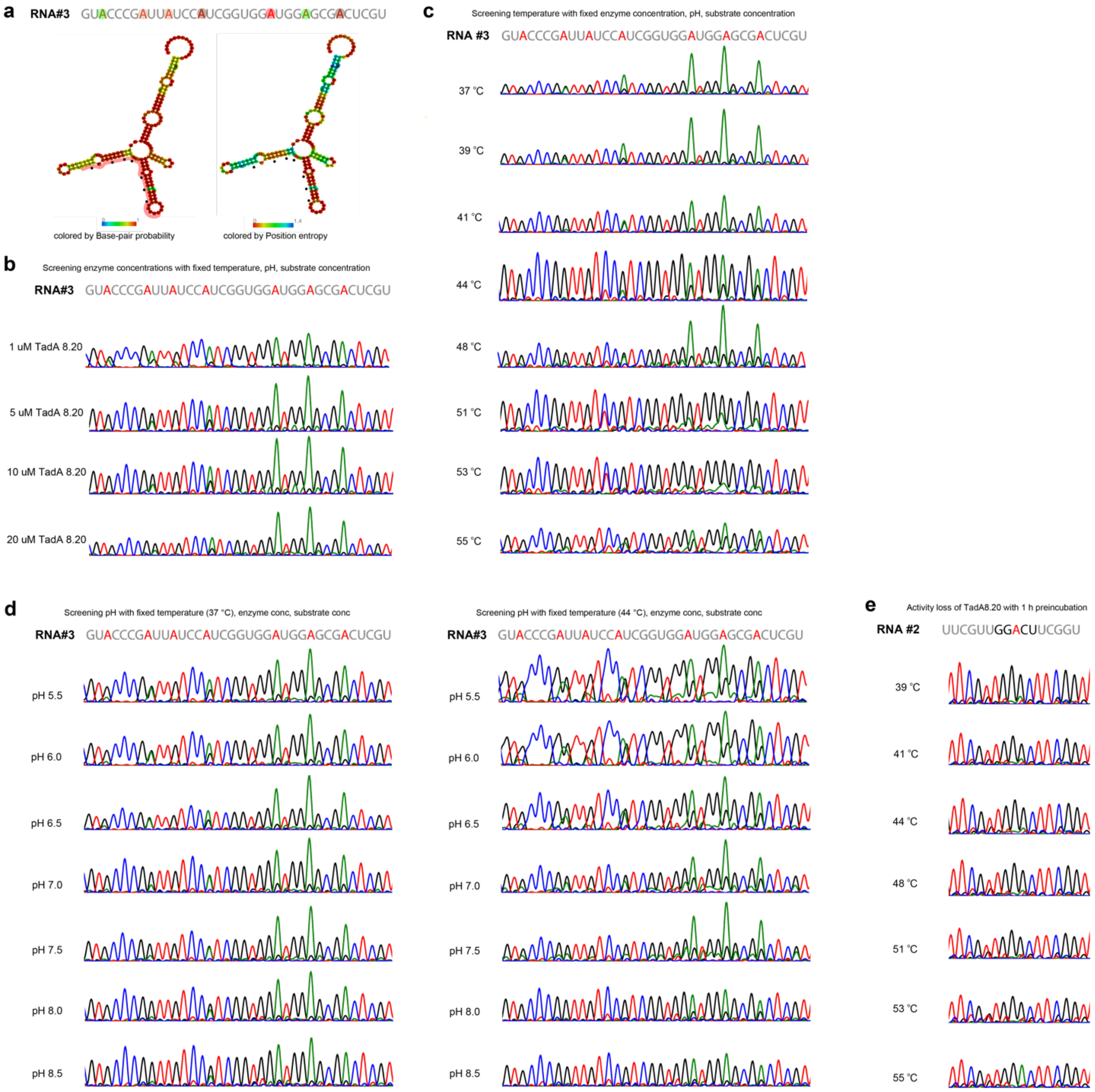

Supplementary Figure 2 | Optimization of assay conditions for TadA8.20-mediated global A deamination. **a**. Predicted secondary structure of RNA #3[4,5]. RNA #3 is an *in vitro* transcribed RNA probe with multiple A sites shielded by secondary structure (**Supplementary Table 5**). We use this probe as a surrogate to evaluate efficiency of TadA8.20-mediated global A deamination. **b-d**. TadA8.20-mediated A-to-I conversion under different enzyme concentrations (**b**), temperatures (**c**), and pH (**d**). **e**. Remaining activity of TadA post-1 h incubation at different temperatures.

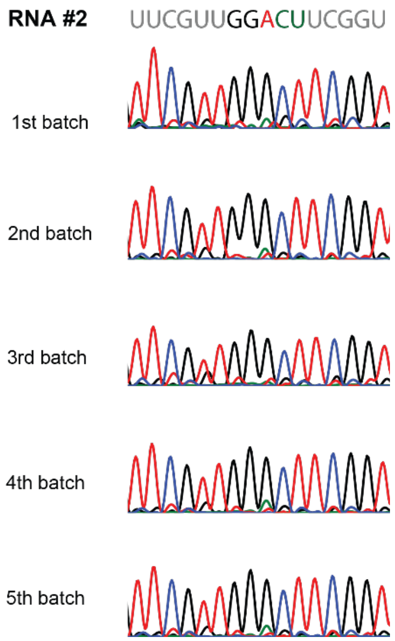

Supplementary Figure 3 | Adenosine deamination activity of Tad8.20 prepared in different batches. All reactions were carried out using the same RNA probe under identical assay conditions (pH 7.5, 37ºC, 1 h).

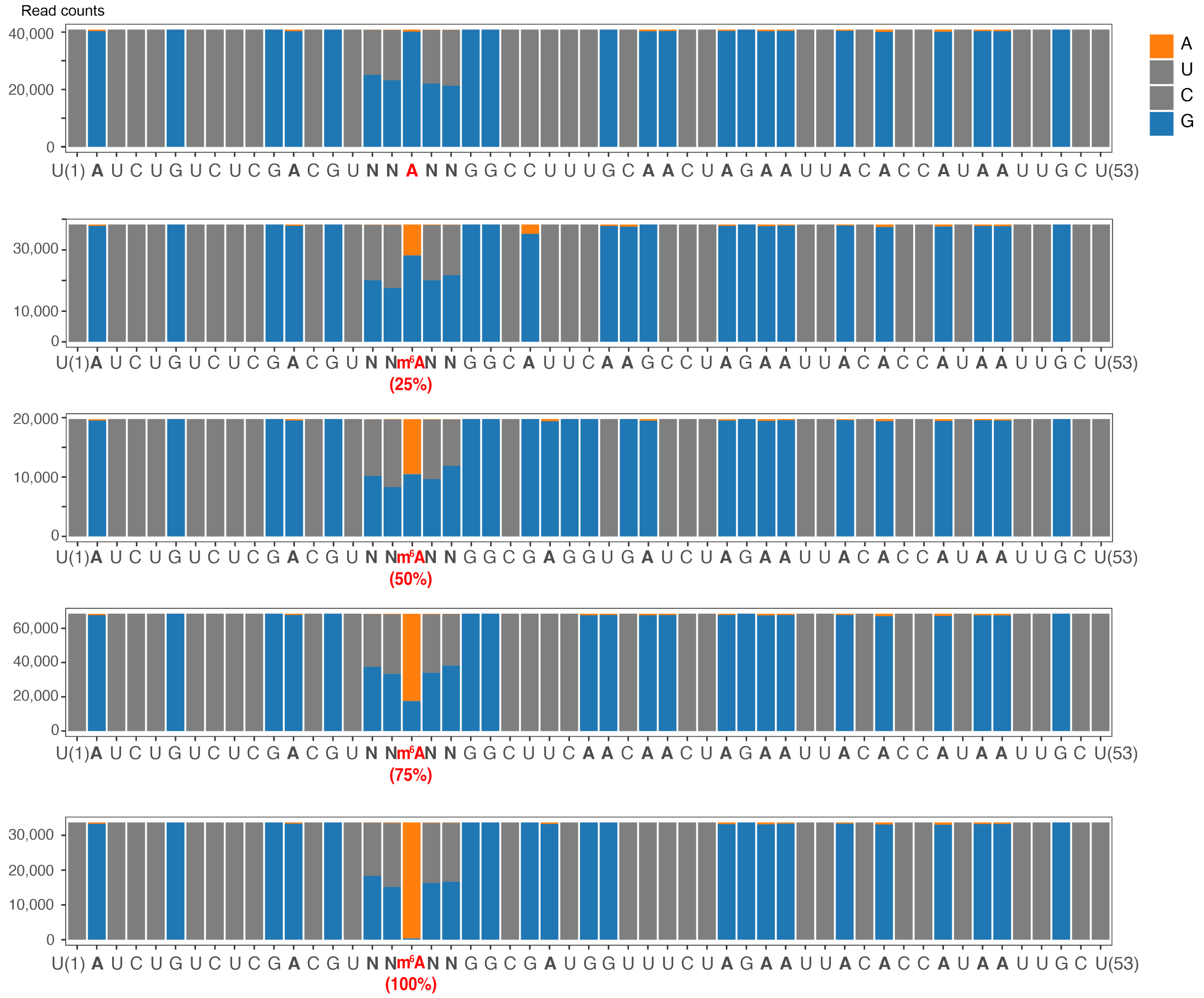

Supplementary Figure 4 | Deamination of synthetic RNA probes carrying 0%, 25%, 50%, 75% and 100% m^6A in NNA/m^6ANN by TadA8.20. RNA was treated by TadA8.20, reverse transcribed, and analyzed by next-generation sequencing.

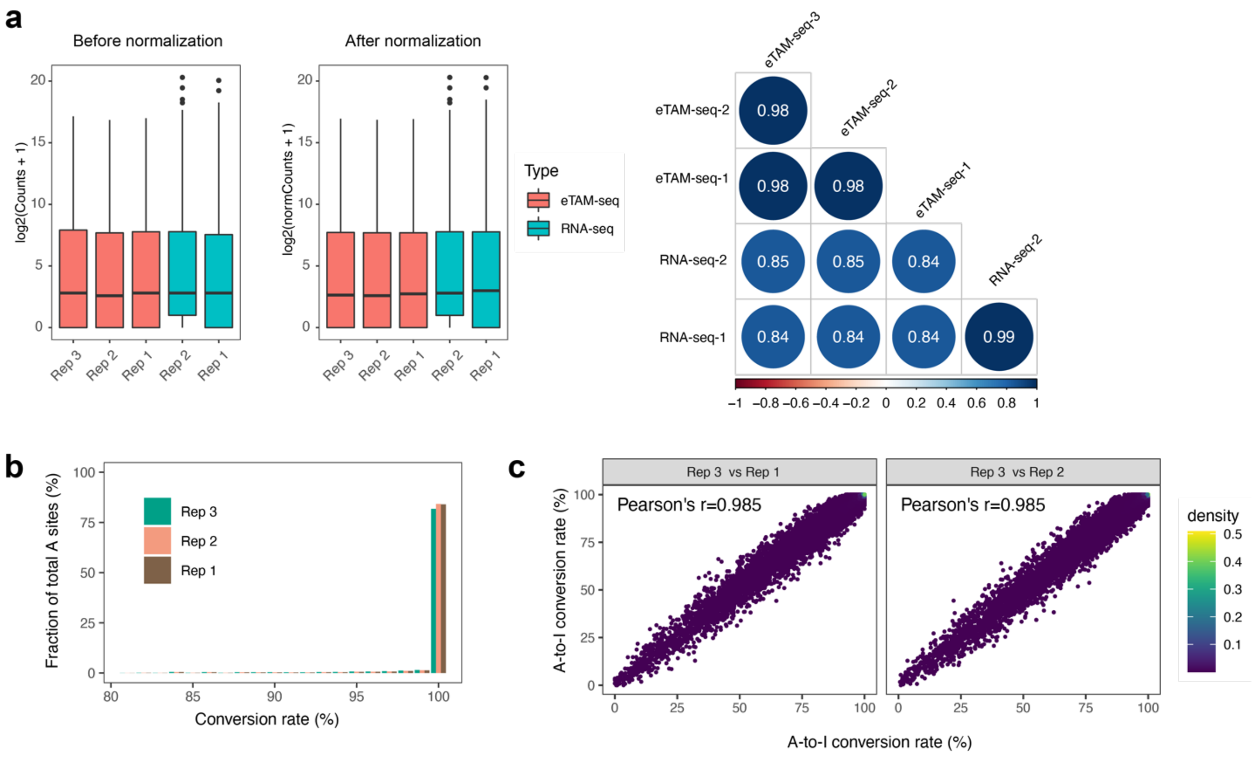

Supplementary Figure 5 | HeLa mRNA treated by TadA8.20. **a**. RNA abundances reported by canonical RNA-seq[6] and eTAM-seq. We down sampled eTAM-seq data to match the depth covered by RNA-seq. In the box plots, lower and upper hinges represent first and third quartiles; the center line represents the median; the red dot represents the mean; and whiskers represent ± 1.5× of the interquartile range. $n = 38{,}600$. **b**. Transcriptome-wide A-to-I conversion rates in three replicates. **c**. Correlation of A-to-I conversion rates in three biological replicates. Scatter plots cover 10% of randomly sampled A sites with ≥ 100 counts. Pearson's r was calculated for all A sites with ≥ 100 counts.

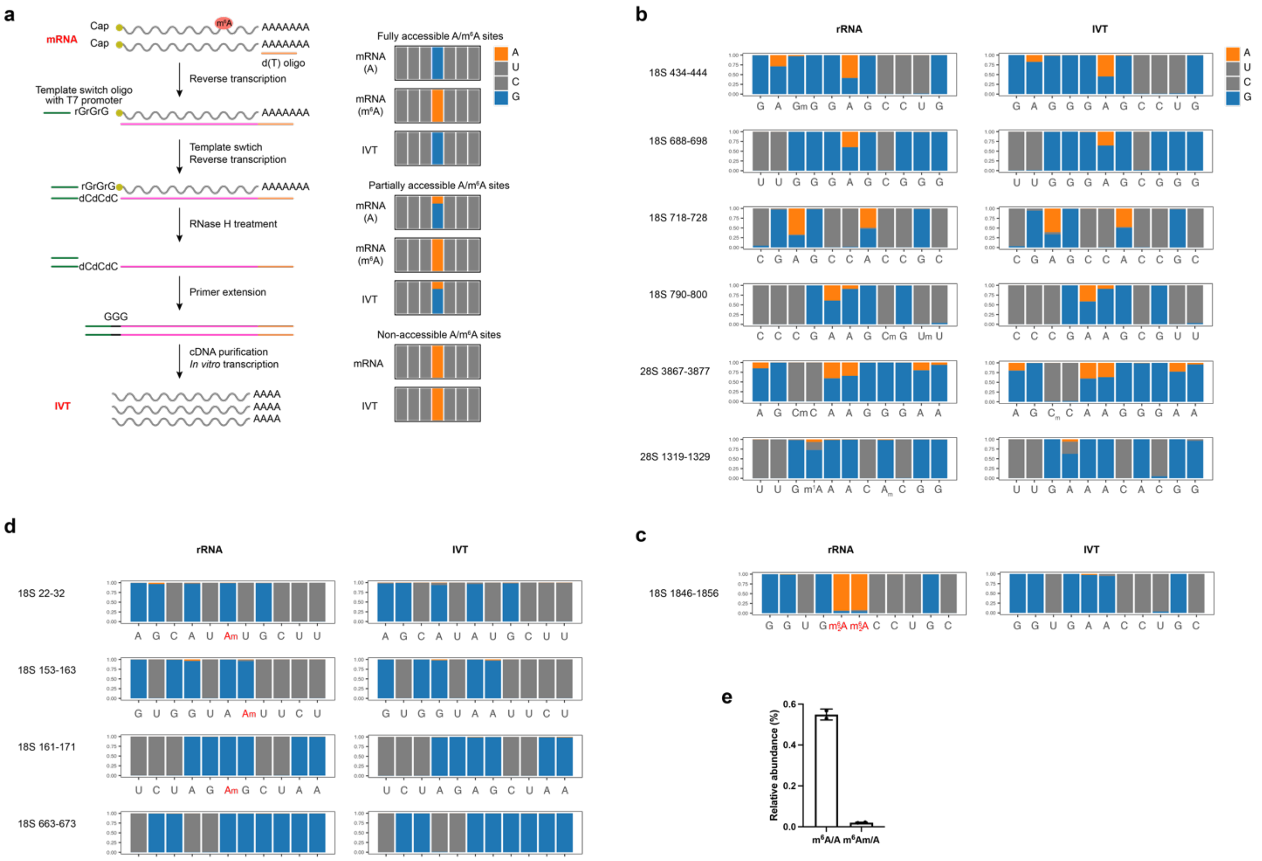

Supplementary Figure 6 | Preparation of an *in vitro* transcribed transcriptome (IVT) and HeLa rRNA treated by TadA8.20. **a**. Preparation of a methylation-free transcriptome by *in vitro* transcription[7,8] and predicted behaviors of fully, partially, and non-accessible A and m^{6}A sites in eTAM-seq. **b**. Selected unmodified A sites resistant to TadA8.20 in human rRNA due to secondary structures. HeLa and *in vitro* transcribed (IVT) RNA samples were both treated with TadA8.20, reverse transcribed, and sequenced. **c**. Two N^6, N^6-dimethyladenosine (m$^6_2$A) sites in human rRNA. **d**. Selected 2′-*O*-methyladenosine (Am) sites in human rRNA. eTAM-seq data of HeLa rRNA and *in vitro* transcribed RNA are plotted side by side for **b**-**d**. **e**. Quantification of m^{6}A and m^{6}Am in poly(A)-free, fragmented, and ligated mRNA by triple quadrupole LC/MS. Results shown are averaged from two independent injections. Error bars represent the standard deviations.

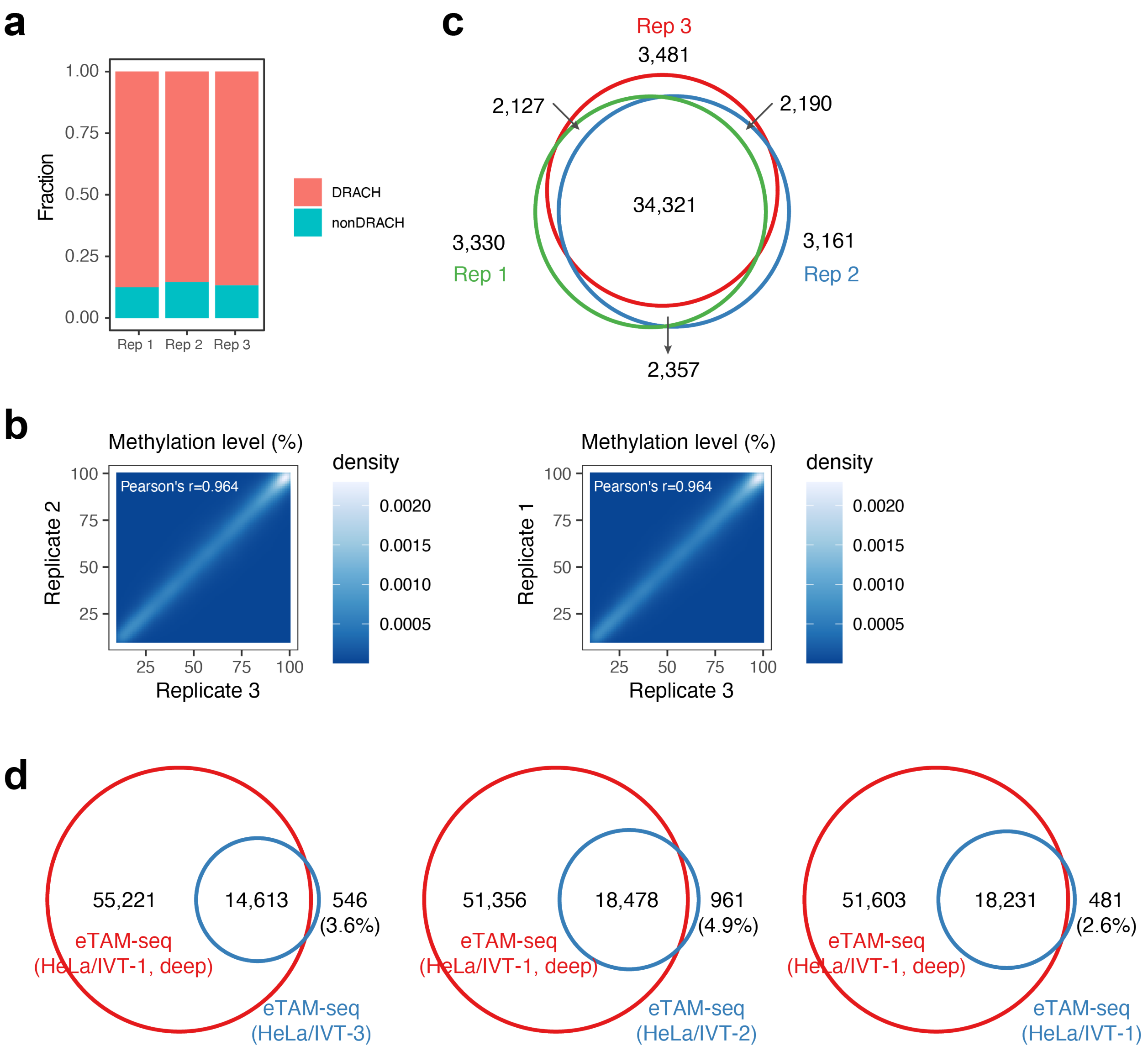

Supplementary Figure 7 | Reproducibility of eTAM-seq (HeLa/IVT). **a**. Hit distributions in DRACH and non-DRACH sequences. Hits are called out using independent IVT controls. **b**. Contour plots of methylation levels reported by three biological replicates. **c**. Overlap analysis of m^{6}A sites identified in three biological replicates of eTAM-seq (HeLa/IVT) with merged IVT controls. **d**. Overlap analysis of m^{6}A sites identified in deep sequenced eTAM-seq (HeLa/IVT-1) and independently processed biological replicates. For more information, see **Supplementary Note 4**.

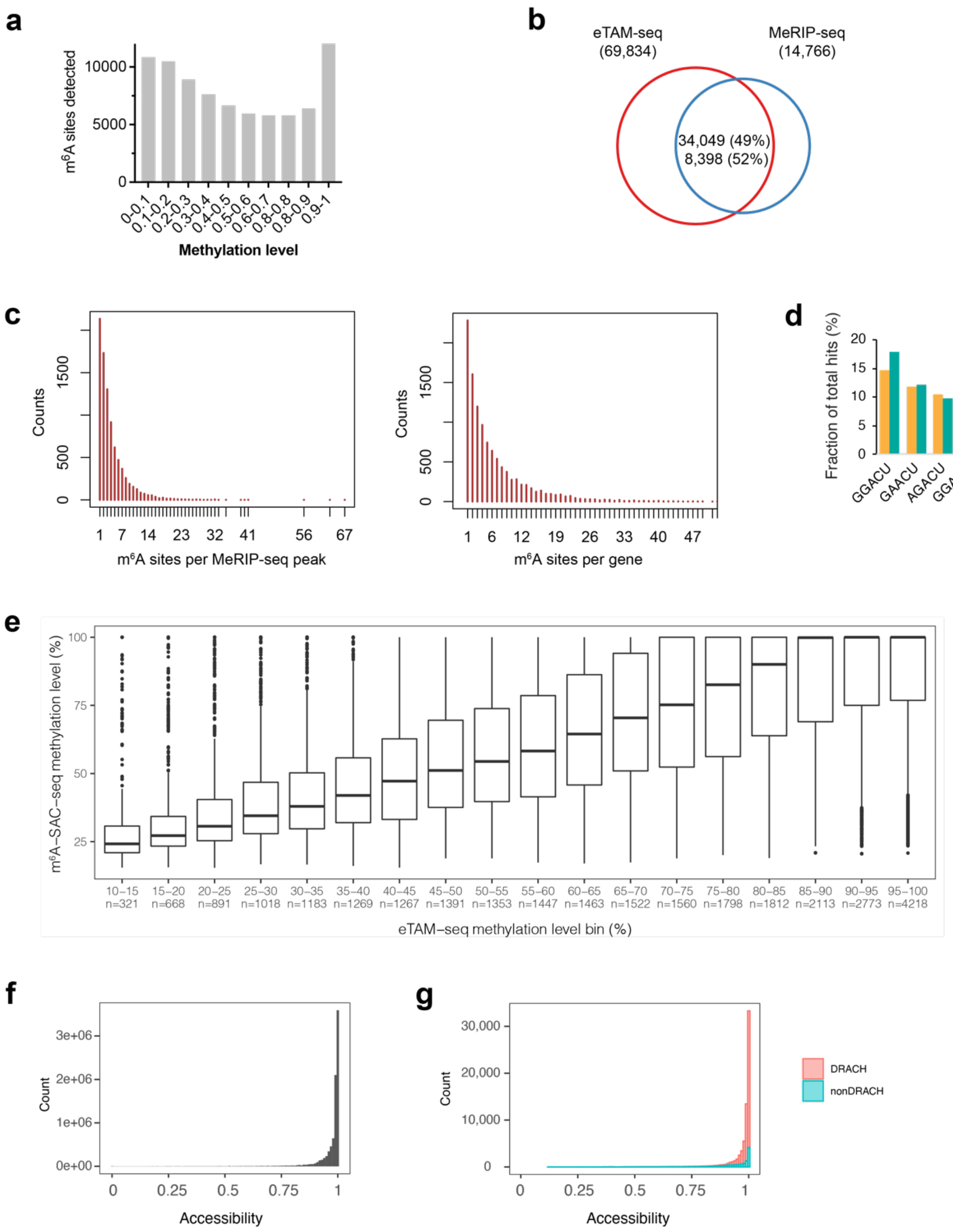

Supplementary Figure 8 | m^{6}A sites captured by deep sequenced eTAM-seq (HeLa/IVT). **a**. Hit distributions among different methylation levels. **b**. Overlap analysis of m^{6}A sites identified by eTAM-seq and peak clusters generated via MeRIP-seq[6]. **c**. Numbers of m^{6}A sites identified per MeRIP-seq peak and per gene. **d**. Major sequence contexts of m^{6}A detected by eTAM-seq and miCLIP[9]. **e**. Comparison of methylation levels for DGACU sites co-discovered by eTAM-seq and m^{6}A-SAC-seq. In the box plots, lower and upper hinges represent first and third quartiles; the center line represents the median; and whiskers represent ± 1.5× of the interquartile range. **f**. Accessibility of all A sites in the HeLa transcriptome. 92% of all evaluated A sites in the HeLa transcriptome showed accessibility ≥ 0.9. **g**. Hit distribution across different accessibility bins.

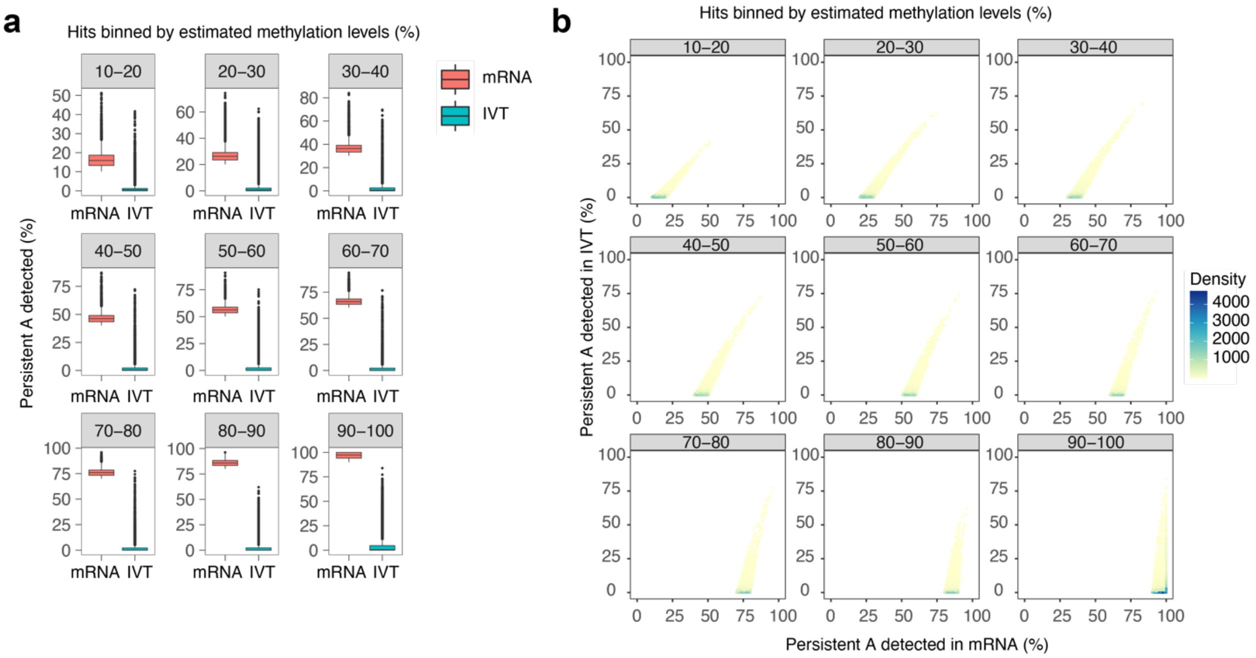

Supplementary Figure 9 | Box plot (**a**) and scatter plot (**b**) showing persistent A signals detected in mRNA and IVT samples. Hits are binned by methylation levels reported by eTAM-seq (HeLa/IVT) (10-20, *n* = 10,250; 20-30, *n* = 8,884; 30-40, *n* = 7,623; 40-50, *n* = 6,655; 50-60, 5,953; 60-70, *n* = 5,799; 70-80, *n* = 5,813; 80-90, *n* = 6,403; 90-100, *n* = 12,454). In the box plots, lower and upper hinges represent first and third quartiles; the center line represents the median; and whiskers represent ± 1.5× of the interquartile range.

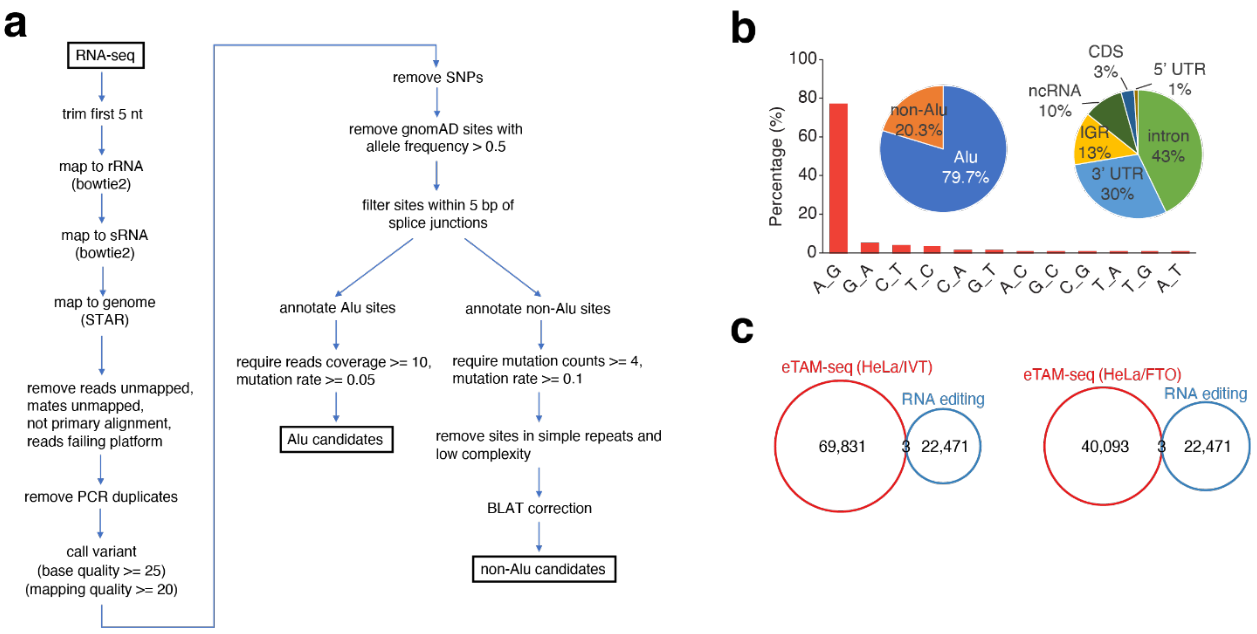

Supplementary Figure 10 | Endogenous RNA editing poses minimal impact on eTAM-seq. **a**. Workflow to call RNA-editing sites. **b**. RNA-editing types and distribution. **c**. Overlap of RNA-editing sites with eTAM-seq hits. Note that RNA editing frequently occurs in repeats. eTAM-seq has lower sensitivity in repeats as we only consider reads uniquely mapped to the genome.

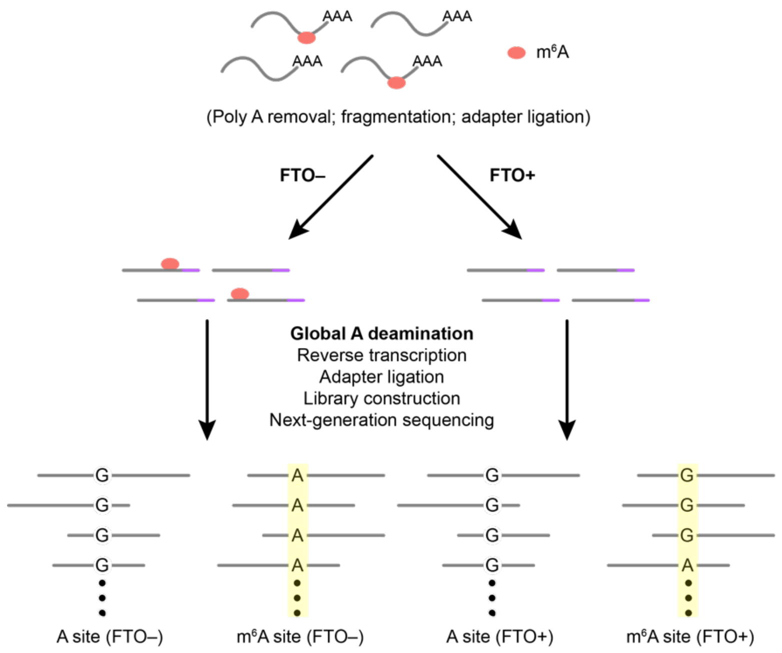

Supplementary Figure 11 | Schematic of transcriptome-wide m^{6}A profiling by eTAM-seq assisted by an N^6-demethylated control transcriptome. m^{6}A sites are identified and quantified by comparing deamination patterns of RNA treated with or without FTO.

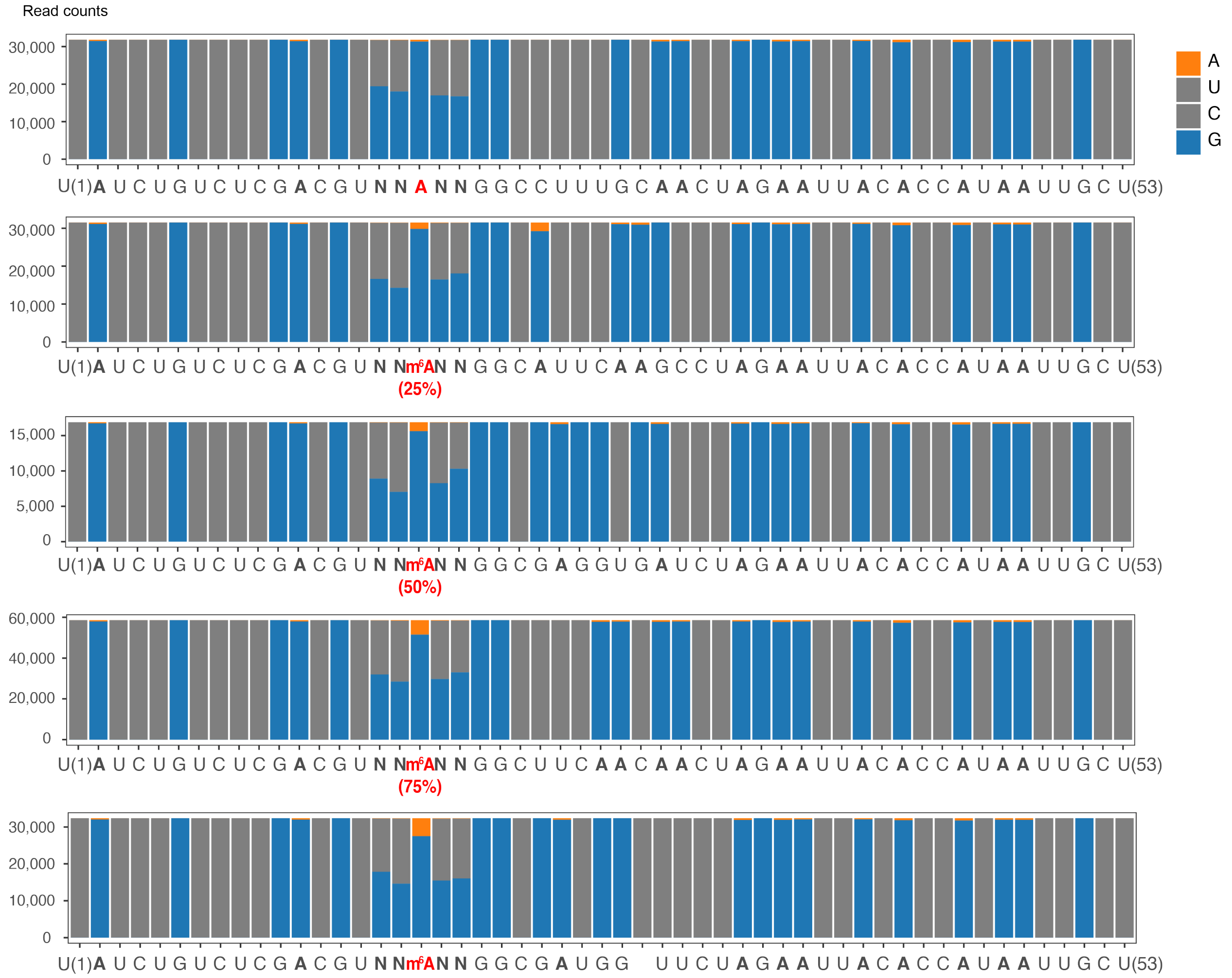

Supplementary Figure 12 | Sequential demethylation and deamination of synthetic RNA probes by FTO and TadA8.20. 53-nt RNA probes that contain 0%, 25%, 50%, 75%, and 100% m^6A in NNA/m^6ANN were treated by FTO, TadA8.20, reverse transcribed, and analyzed by next-generation sequencing.

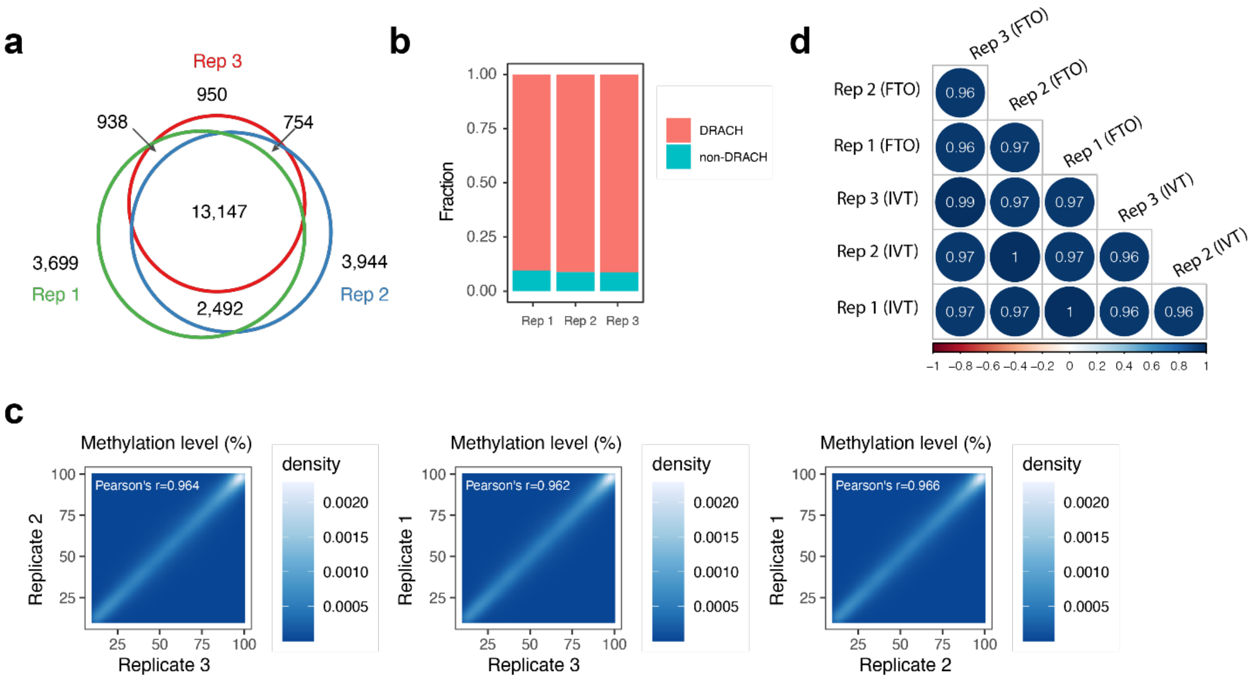

Supplementary Figure 13 | Reproducibility of eTAM-seq with an FTO-treated control transcriptome. **a**. Overlap analysis of m^{6}A sites identified in three biological replicates of eTAM-seq (HeLa/FTO). **b**. Hit distributions in DRACH and non-DRACH sequences. **c**. Comparison of methylation levels reported by three biological replicates of eTAM-seq (HeLa/FTO). **d**. Correlation of methylation levels reported by three biological replicates of eTAM-seq (HeLa/IVT) and eTAM-seq (HeLa/FTO) in Pearson's r.

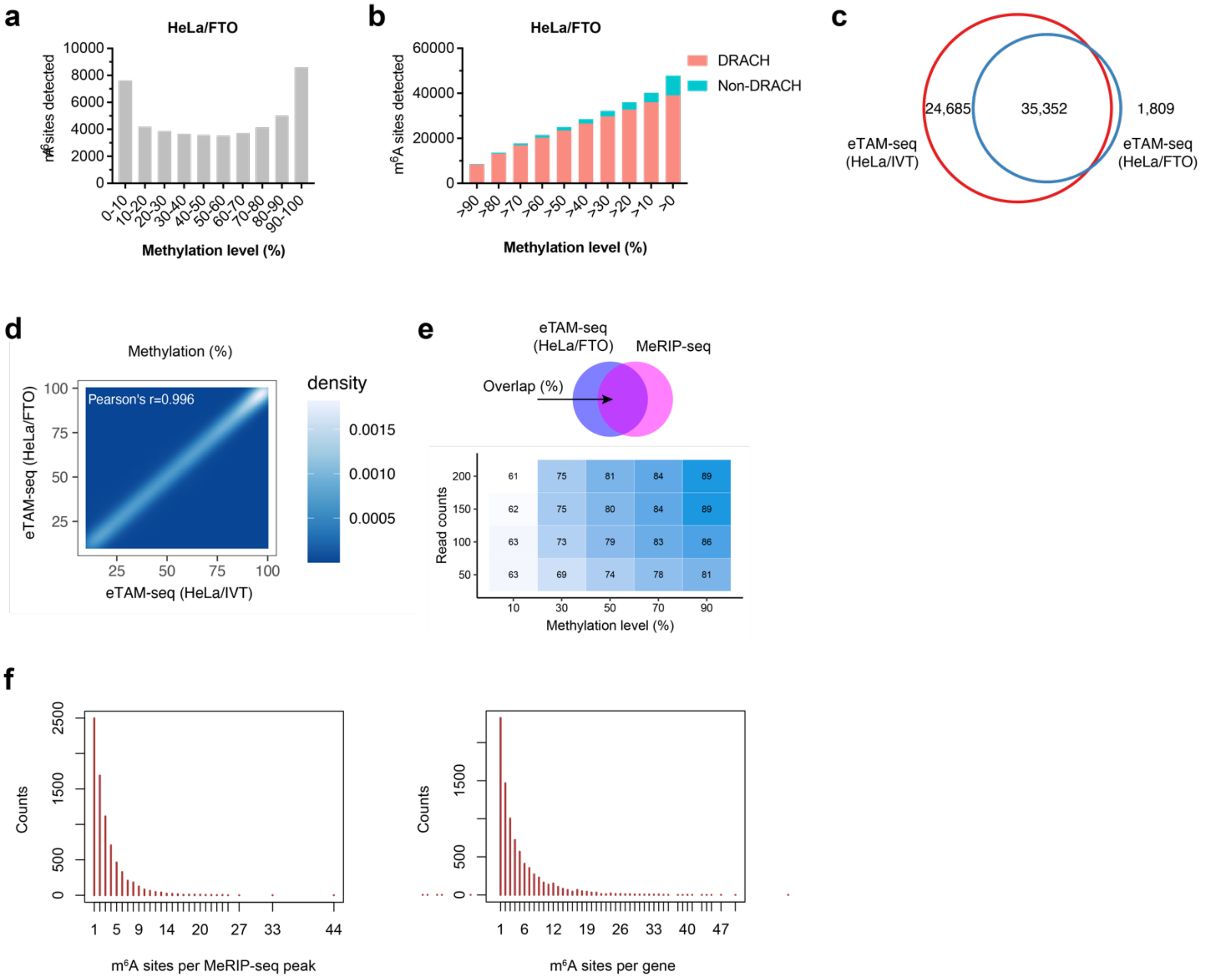

Supplementary Figure 14 | m^{6}A profiling in HeLa cells referenced to an FTO-treated transcriptome. **a**. Hit distributions across different methylation levels. **b**. Hit distributions in DRACH and non-DRACH sequences at different methylation levels. **c**. Overlap analysis of eTAM-seq (HeLa/IVT) and eTAM-seq (HeLa/FTO). The two reference transcriptomes (IVT and FTO) were prepared separately with orthogonal protocols, thereby serving as independent controls. Only A sites sampled in both datasets are considered. **d**. Methylation levels reported by eTAM-seq (HeLa/IVT) and eTAM-seq (HeLa/FTO). **e**. Overlap analysis of m^{6}A sites identified by eTAM-seq (HeLa/FTO) and peak clusters generated by MeRIP-seq[6]. **f**. Numbers of m^{6}A sites identified per MeRIP-seq peak and per gene.

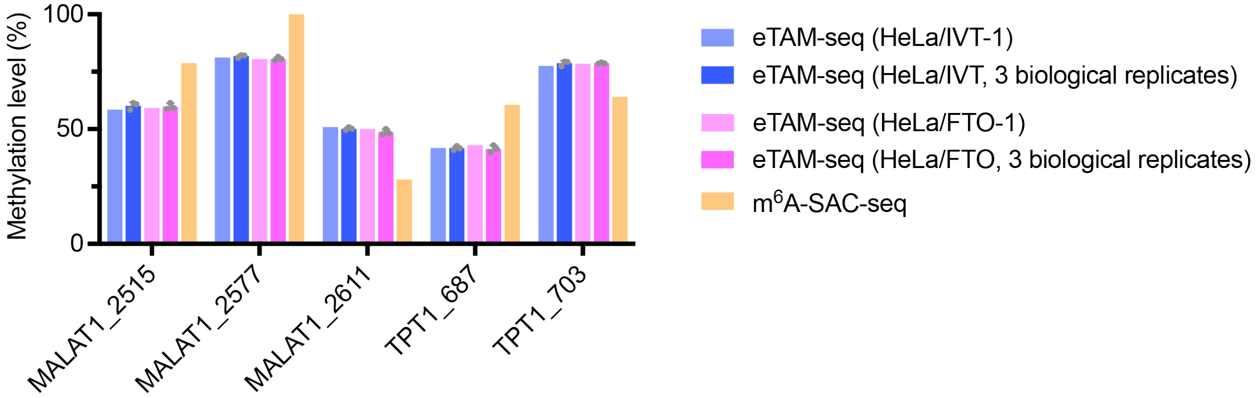

Supplementary Figure 15 | Methylation levels at MALAT1_2515, 2577, 2611 and TPT1_687, 703 reported by eTAM-seq (HeLa/IVT), eTAM-seq (HeLa/FTO), and m^{6}A-SAC-seq[10]. Error bars represent the standard deviations when results from three biological replicates are considered.

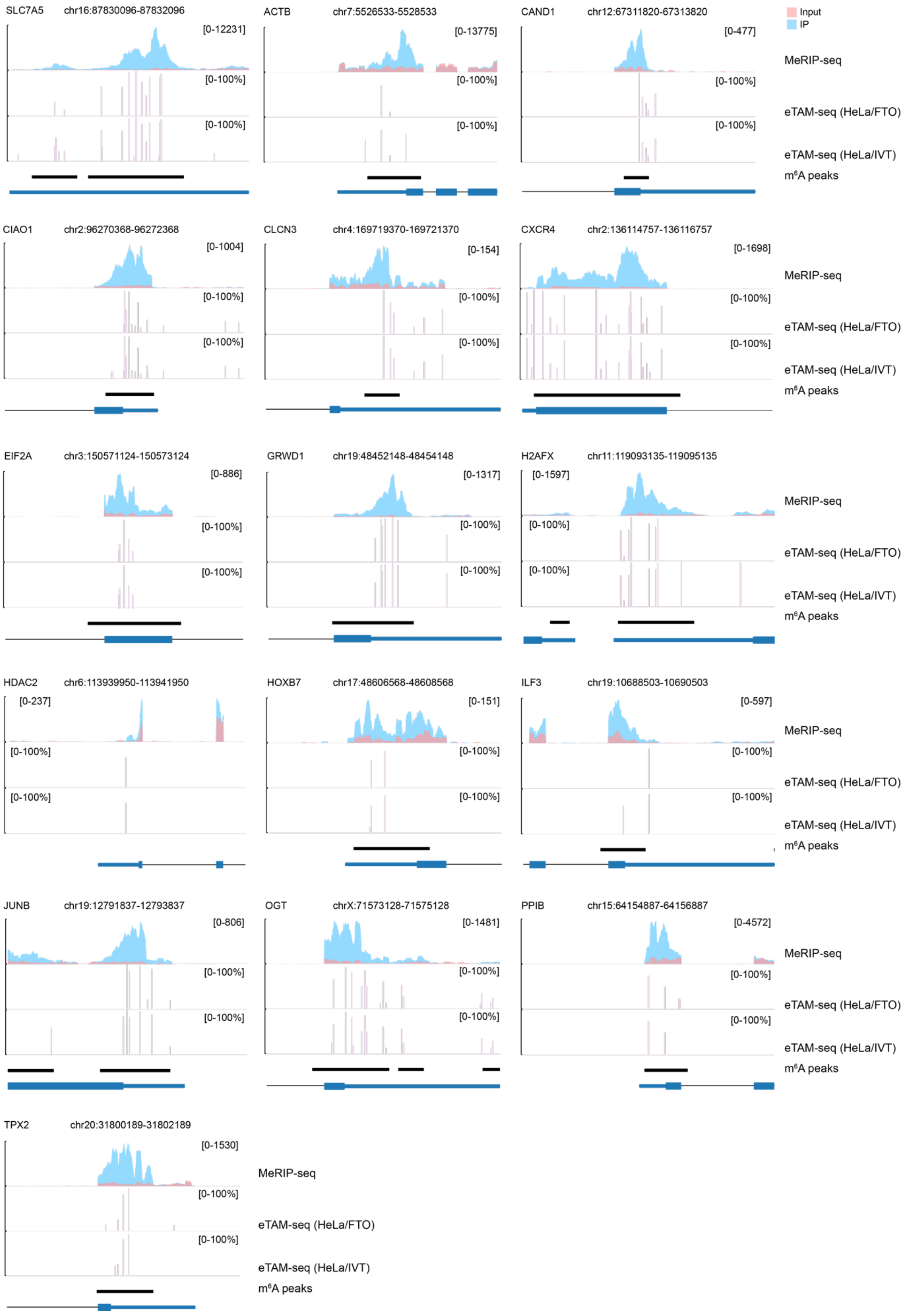
SLC7A5 chr16:87830096-87832096
[0-12231]
[0-100%]
[0-100%]
ACTB chr7:5526533-5528533
[0-13775]
[0-100%]
[0-100%]
CAND1 chr12:67311820-67313820
[0-477]
[0-100%]
[0-100%]
Input
IP
MeRIP-seq
eTAM-seq (HeLa/FTO)
eTAM-seq (HeLa/IVT)
m6A peaks
CIAO1 chr2:96270368-96272368
[0-1004]
[0-100%]
[0-100%]
CLCN3 chr4:169719370-169721370
[0-154]
[0-100%]
[0-100%]
CXCR4 chr2:136114757-136116757
[0-1698]
[0-100%]
[0-100%]
MeRIP-seq
eTAM-seq (HeLa/FTO)
eTAM-seq (HeLa/IVT)
m6A peaks
EIF2A chr3:150571124-150573124
[0-886]
[0-100%]
[0-100%]
GRWD1 chr19:48452148-48454148
[0-1317]
[0-100%]
[0-100%]
H2AFX chr11:119093135-119095135
[0-1597]
[0-100%]
[0-100%]
MeRIP-seq
eTAM-seq (HeLa/FTO)
eTAM-seq (HeLa/IVT)
m6A peaks
HDAC2 chr6:113939950-113941950
[0-237]
[0-100%]
[0-100%]
HOXB7 chr17:48606568-48608568
[0-151]
[0-100%]
[0-100%]
ILF3 chr19:10688503-10690503
[0-597]
[0-100%]
[0-100%]
MeRIP-seq
eTAM-seq (HeLa/FTO)
eTAM-seq (HeLa/IVT)
m6A peaks
JUNB chr19:12791837-12793837
[0-806]
[0-100%]
[0-100%]
OGT chrX:71573128-71575128
[0-1481]
[0-100%]
[0-100%]
PPIB chr15:64154887-64156887
[0-4572]
[0-100%]
[0-100%]
MeRIP-seq
eTAM-seq (HeLa/FTO)
eTAM-seq (HeLa/IVT)
m6A peaks
TPX2 chr20:31800189-31802189
[0-1530]
[0-100%]
[0-100%]
MeRIP-seq
eTAM-seq (HeLa/FTO)
eTAM-seq (HeLa/IVT)
m6A peaks

Supplementary Figure 16 | m^6A positions and fractions in 16 HeLa transcripts: SLC7A5, ACTB, EIF2A, HDAC2, ILF3, CAND1, CIAO1, OGT, TPX2, HOXB7, CXCR4, JUNB, GRWD1, H2AFX, PPIB, and CLCN3. eTAM-seq results are plotted below MeRIP-seq peaks. Normalized read coverage is plotted in MeRIP-seq tracks. Note that eTAM-seq (HeLa/IVT) has slightly higher coverage than eTAM-seq (HeLa/FTO) and may therefore capture more m^6A sites.

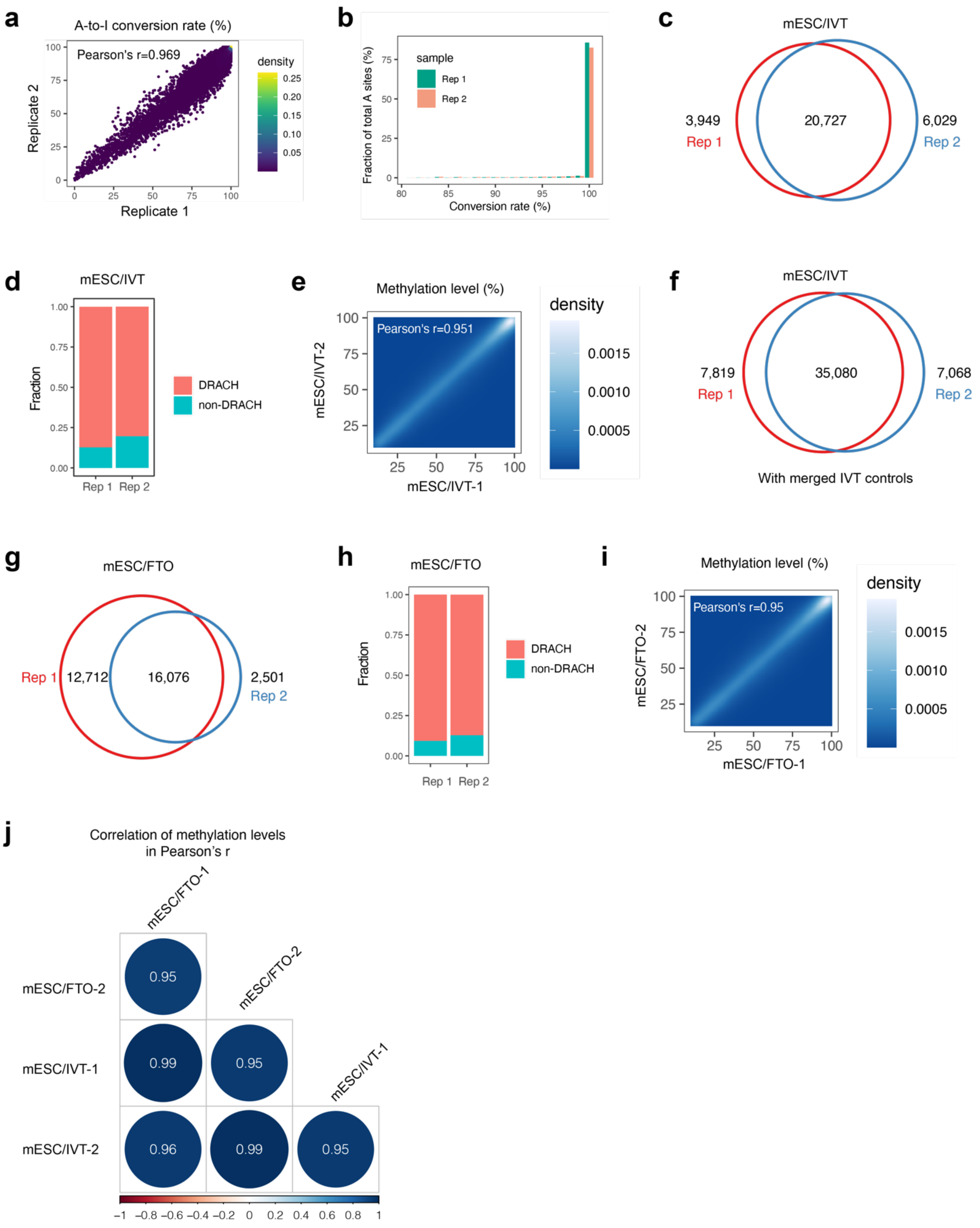

Supplementary Figure 17 | Biological replicates for mESCs. **a**. Correlation of A-to-I conversion rates in two replicates. 10% of A sites with ≥ 100 counts were randomly sampled to make the scatter plot. Pearson's r was calculated using all A sites with ≥ 100 counts. **b**. Transcriptome-wide

A-to-I conversion rates. **c**. Overlap analysis of m^6A sites identified in eTAM-seq (mESC/IVT-1) and eTAM-seq (mESC/IVT-2). **d**. Hit distributions in DRACH and non-DRACH motifs for eTAM-seq (mESC/IVT). **e**. Correlation of methylation levels reported by eTAM-seq (mESC/IVT-1) and eTAM-seq (mESC/IVT-2). **f**. Overlap analysis of m^6A sites identified by eTAM-seq (mESC/FTO-1) and eTAM-seq (mESC/FTO-2). **h**. Hit distributions in DRACH and non-DRACH motifs for eTAM-seq (mESC/FTO). **i**. Correlation of methylation levels reported by eTAM-seq (mESC/FTO-1) and eTAM-seq (mESC/FTO-2). **j**. Correlation of methylation levels reported by two biological replicates of eTAM-seq (mESC/IVT) and eTAM-seq (mESC/FTO) in Pearson's r.

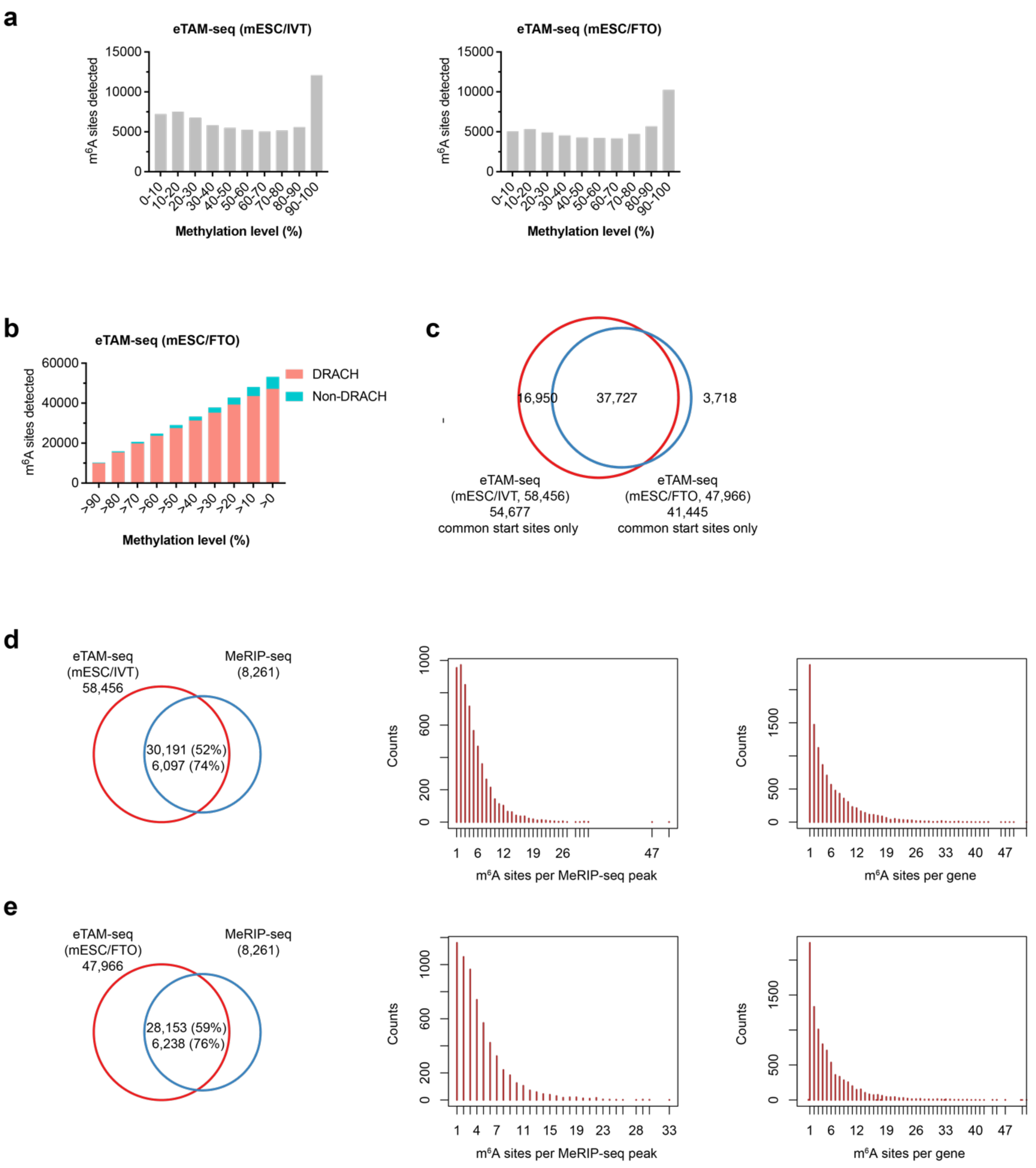

Supplementary Figure 18 | m^6A profiling in mESCs. **a**. Hit distributions among different methylation levels. Hits identified by eTAM-seq (mESC/IVT) and eTAM-seq (mESC/FTO) are presented side by side. **b**. Distributions of m^6A sites identified by eTAM-seq (mESC/FTO) in DRACH and non-DRACH motifs at different methylation levels. **c**. Overlap analysis of m^6A sites identified by eTAM-seq (mESC/IVT) and eTAM-seq (mESC/FTO). A sites sampled in both datasets are considered. **d**. Overlap analysis of m^6A sites identified by eTAM-seq (mESC/IVT) and peak clusters generated via MeRIP-seq[7]. **e**. Overlap analysis of m^6A sites identified by eTAM-seq (mESC/FTO) and peak clusters generated via MeRIP-seq[7]. Single MeRIP-seq peaks and individual genes cover multiple m^6A sites.

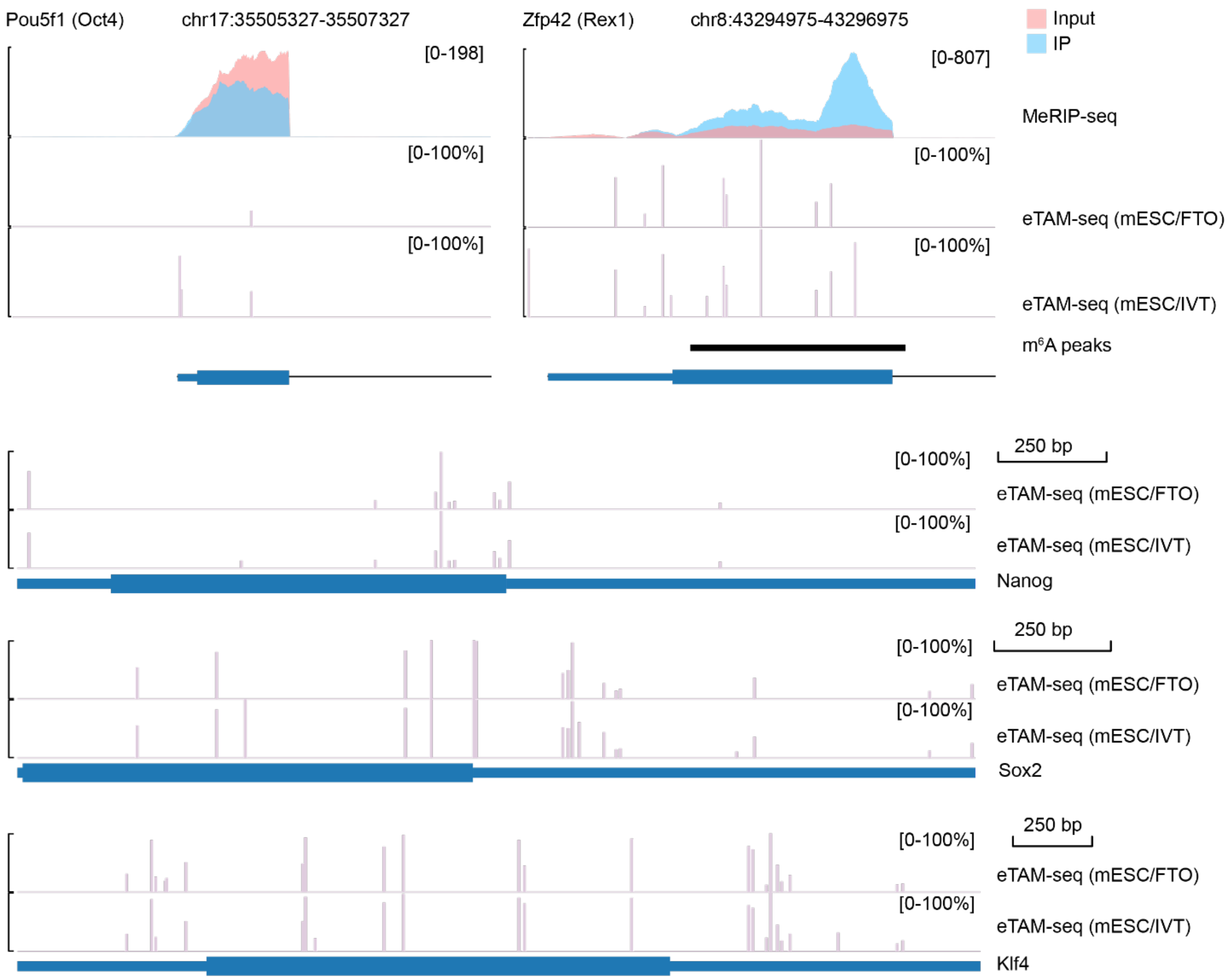

Supplementary Figure 19 | m^{6}A positions and fractions in selected regions of Oct4 and Rex1 (top) and full-length Nanog, Sox2, and Klf4 (bottom) in mESCs. eTAM-seq results are plotted below MeRIP-seq peaks. Normalized read coverage is plotted in MeRIP-seq tracks.

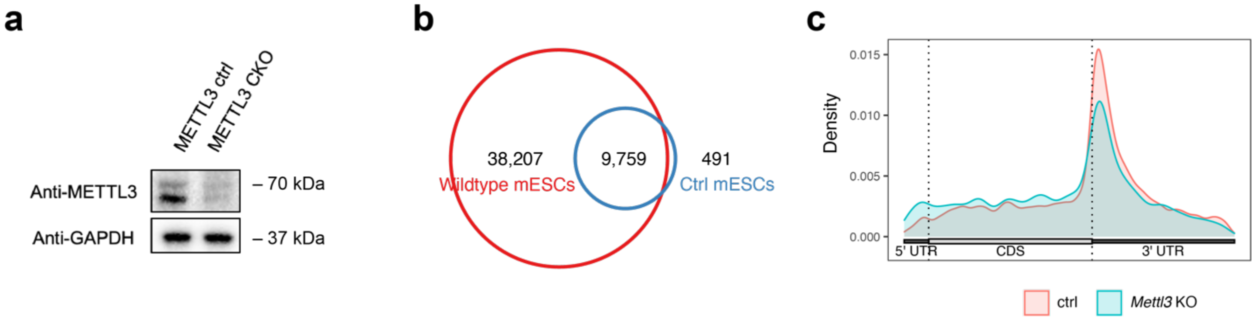

Supplementary Figure 20 | m^{6}A profiling in ctrl and *Mettl3* KO mESCs. **a**. Western blot showing successful knock out of *Mettl3*. Experiments were repeated independently at least three times with similar results. **b**. Overlap analysis of m^{6}A sites detected in ctrl mESCs and wildtype mESCs, both by eTAM-seq (mESC/FTO). **c**. Metagene plot of transcriptome-wide distribution of m^{6}A in ctrl and *Mettl3* KO mESCs.

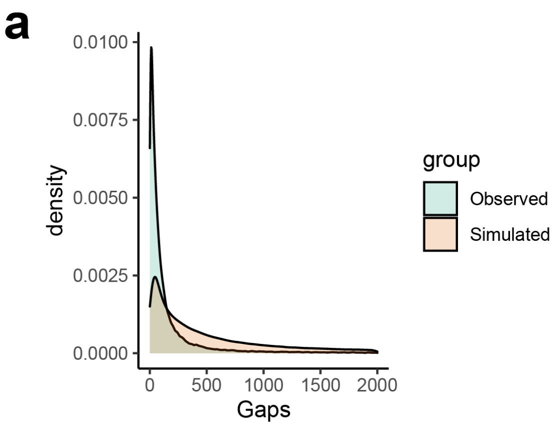

Supplementary Figure 21 | Simulated and observed distances of one m^{6}A site to its nearest neighbor. Two simulations were carried out with no context constraint (**a**) or by forcing m^{6}A-carrying 5-nt motifs to match the frequencies observed in eTAM-seq (**b**).

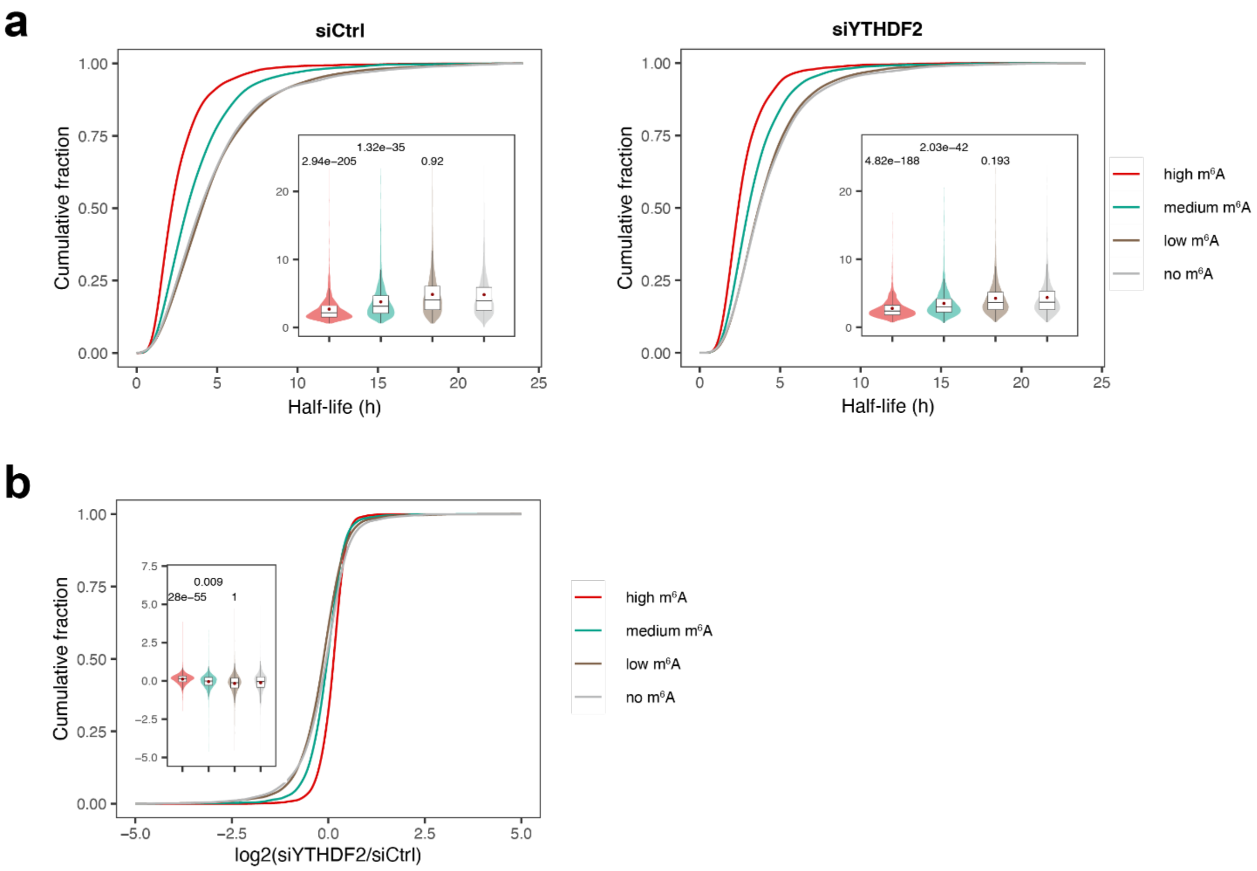

Supplementary Figure 22 | YTHDF2 regulates the stability of m^6A-modified mRNA in HeLa cells. **a**. Cumulative half-life distributions for transcripts in HeLa cells treated with control and YTHDF2-targeting siRNA. **b**. Cumulative distribution for changes of transcript half-lives in HeLa cells treated with control and YTHDF2-targeting siRNA. Transcripts methylated to different levels (high m^6A: n = 2,385; medium m^6A: n = 2,386; low m^6A: n = 2,385; and no m^6A: n = 3,108) are plotted separately with box violin plots inserted. Lower and upper hinges represent first and third quartiles; the center line represents the median; the red dot represents the mean; and whiskers represent ± 1.5× of the interquartile range. P-values were determined by one-tailed Wilcoxon rank-sum test using the unmethylated group as a reference. HeLa mRNA half-life dataset: GSE49339[11].

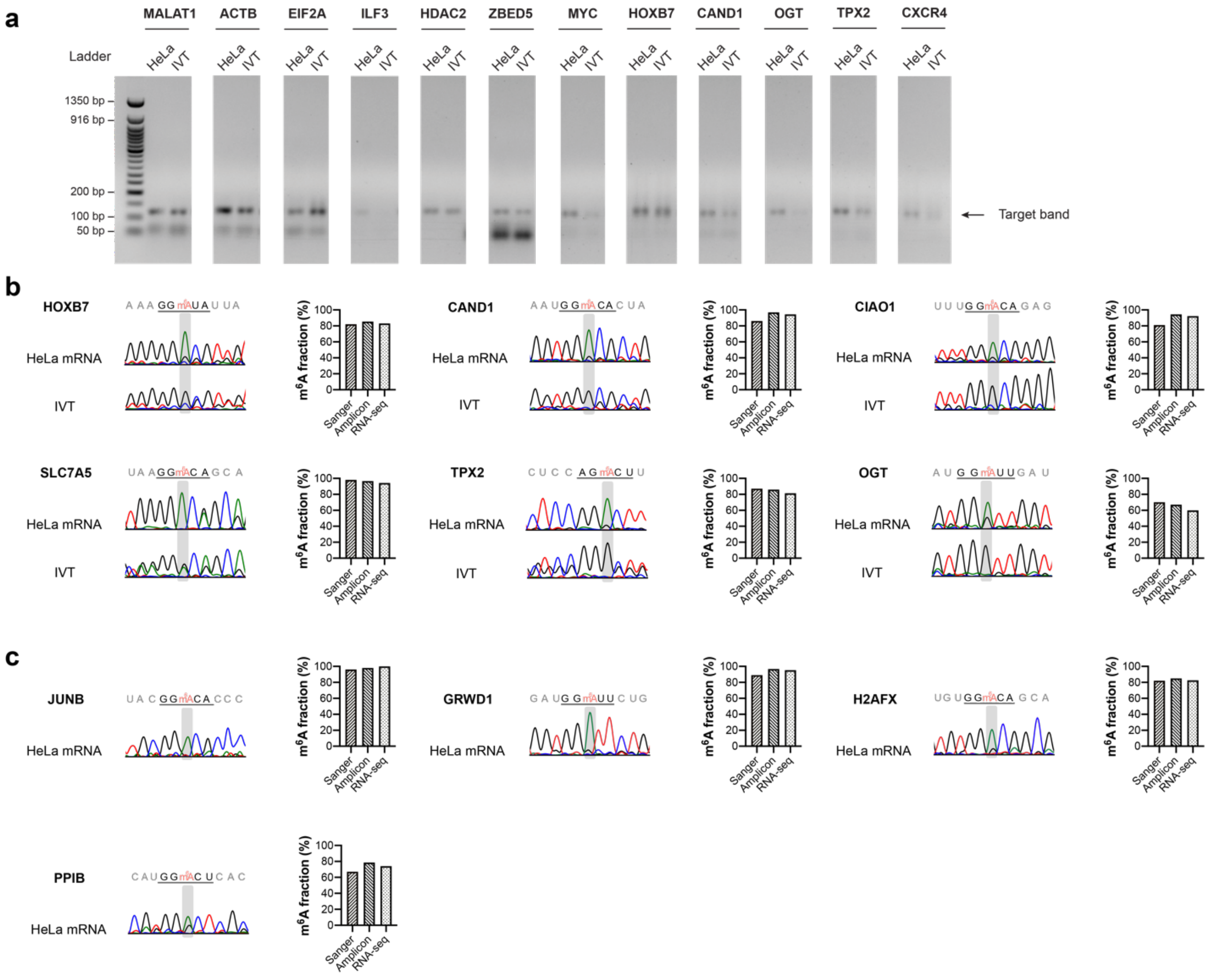

Supplementary Figure 23 | Detection and quantification of m^{6}A by eTAM-seq. **a**. RT-qPCR products covering m^{6}A-bearing regions analyzed by agarose gel electrophoresis. Experiments were carried out twice independently with similar results. **b**. Quantification of 6 additional m^{6}A sites in HeLa mRNA by Sanger sequencing, amplicon deep sequencing, and RNA-seq. **c**. m^{6}A quantification in the absence of IVT controls.

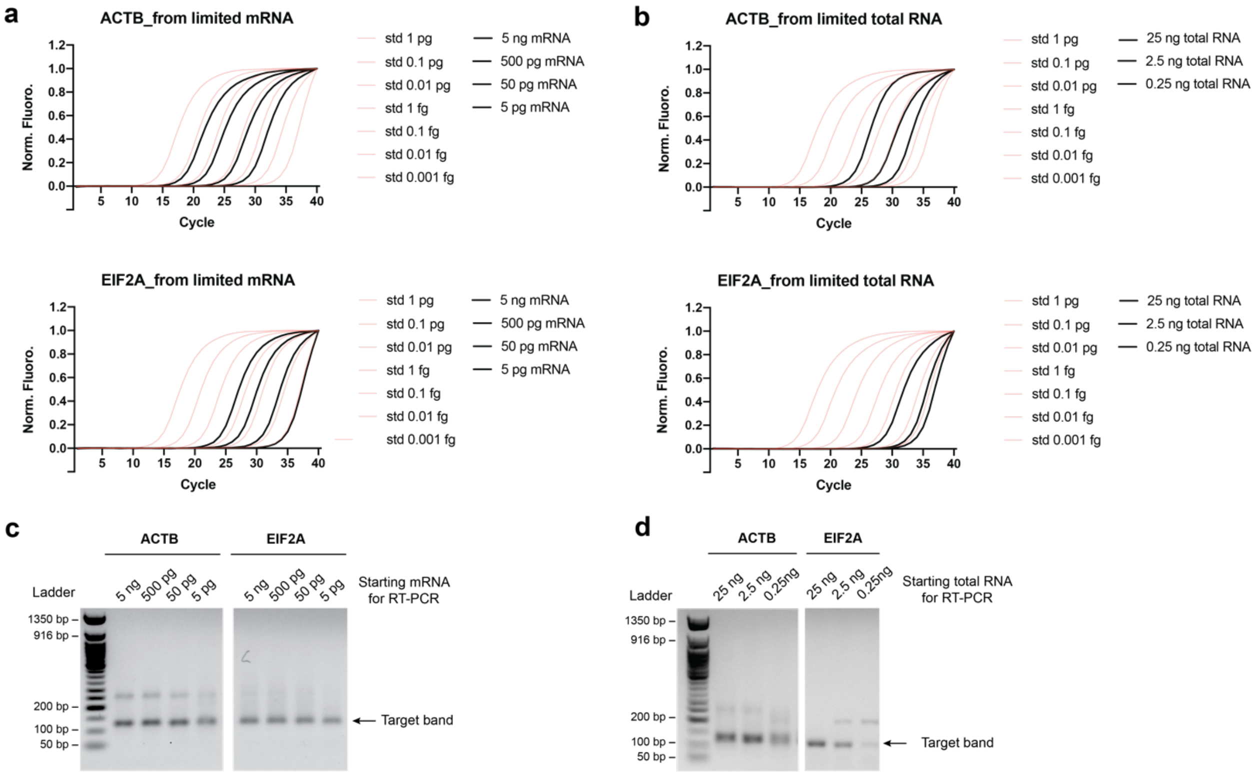

Supplementary Figure 24 | Amplification of m^{6}A-bearing transcripts from Tad8.20-treated mRNA and total RNA. **a**. RT-qPCR traces showing the amplification of fragments covering ACTB-1427 and EIF2A-994 from diluted cDNA. DNA standards were prepared by diluting ssDNA encoding the EIF2A sequence with all A replaced by G. std: standard. **b**. RT-qPCR amplification of fragments covering ACTB-1427 and EIF2A-994 from limited amounts of total RNA. **c**,**d**. Analysis of RT-qPCR products by agarose gel electrophoresis. Experiments were carried out twice independently with similar results.

References

1. Bass, B.L. RNA editing by adenosine deaminases that act on RNA. *Annu. Rev. Biochem.* **71**, 817-846 (2002).
2. Kluesner, M.G. et al. EditR: a method to quantify base editing from Sanger sequencing. *CRISPR J.* **1**, 239-250 (2018).
3. Liu, N. et al. Probing *N^6*-methyladenosine RNA modification status at single nucleotide resolution in mRNA and long noncoding RNA. *RNA* **19**, 1848-1856 (2013).
4. Gruber, A.R., Lorenz, R., Bernhart, S.H., Neubock, R. & Hofacker, I.L. The Vienna RNA websuite. *Nucleic Acids Res.* **36**, W70-74 (2008).
5. Lorenz, R. et al. ViennaRNA package 2.0. *Algorithms Mol. Biol.* **6**, 26 (2011).
6. Liu, J. et al. A METTL3-METTL14 complex mediates mammalian nuclear RNA *N^6*-adenosine methylation. *Nat. Chem. Biol.* **10**, 93-95 (2014).
7. Zhang, Z. et al. Systematic calibration of epitranscriptomic maps using a synthetic modification-free RNA library. *Nat. Methods.* **18**, 1213-1222 (2021).
8. Hagemann-Jensen, M. et al. Single-cell RNA counting at allele and isoform resolution using Smart-seq3. *Nat. Biotechnol.* **38**, 708-714 (2020).
9. Linder, B. et al. Single-nucleotide-resolution mapping of m6A and m6Am throughout the transcriptome. *Nat. Methods* **12**, 767-772 (2015).
10. Ge, R. et al. m(6)A-SAC-seq for quantitative whole transcriptome m(6)A profiling. *Nat. Protoc.*, accepted (2022).
11. Wang, X. et al. N6-methyladenosine-dependent regulation of messenger RNA stability. *Nature* **505**, 117-120 (2014).

Transcriptome-Wide Profiling and Quantification of N6-Methyladenosine by Enzyme-Assisted Adenosine Deamination

Yulan Xiao, Shun Liu, Ruiqi Ge, Yuan Wu, Chuan He, Mengjie Chen and Weixin Tang

Supplementary Note 3

Model for an untreated m6A sample with an IVT control

1 Model Annotations

We introduce the following global parameters:

- k_1: Conversion rate for the untreated m6A sample
- k_2: Conversion rate for the IVT control sample

Look at each site i:

- θ_i: True A proportion, i.e., the underlying proportion of A in total A (including A and m6A) without any treatment
- ω_i: Site-specific accessibility

Expected proportion of nucleotide A at each site i can be represented as:

$$R_{iA}^{-} = 1 - k_1\omega_i\theta_i$$
$$R_{iA}^{\text{ivt}} = 1 - k_2\omega_i\theta_i$$

Expected proportion of nucleotide G at each site i can be represented as:

$$R_{iG}^{-} = k_1\omega_i\theta_i$$
$$R_{iG}^{\text{ivt}} = k_2\omega_i\theta_i$$

Ideally, if $k_1 = k_2 = \omega_i = 1$, $R_{iG}^{-} = \theta_i$, $R_{iG}^{\text{ivt}} = 1$, we can use $R_{iG}^{\text{ivt}} - R_{iG}^{-} = 1 - \theta_i$ to estimate the abundance of m6A. However, in reality, the conversion rates, and site accessibility are not all 100%, $R_{iG}^{\text{ivt}} - R_{iG}^{-} = (k_2 - k_1)\omega_i\theta_i$. We need to estimate conversion rates and site-specific accessibility to quantify the actual methylation level.

2 Estimating conversion rates

Majority of A sites are accessible and un-methylated. For un-methylated and fully accessible A sites, we have:

$$R_{iG}^{-} = k_1$$
$$R_{iG}^{\text{ivt}} = k_2$$

Let $\kappa = k_1/k_2$, $\delta = k_1 - k_2$, r_{iG}^{-} and r_{iG}^{ivt} represent observed G rates, respectively, for each site i, we calculate:

$$\kappa_i = r_{iG}^{-}/r_{iG}^{\text{ivt}}$$
$$\delta_i = r_{iG}^{-} - r_{iG}^{\text{ivt}}$$

$\hat{\kappa} = \text{Median}(\kappa_i)$ and $\hat{\delta} = \text{Median}(\delta_i)$ where i belong to sites after removing 10% outliers. Finally, we estimate conversion rates by:

$$\hat{k_2} = \frac{\hat{\delta}}{\hat{\kappa} - 1}$$
$$\hat{k_1} = \hat{\kappa}\hat{k_2}$$

3 Estimating apparent methylation level

Let us denote apparent methylation level using $\beta_i = \omega_i \theta_i$. Assuming the sampling of G following a binomial model, we use the untreated mRNA sample to quantify apparent methylation level $\hat{\beta}_i$ for each site.

4 Estimating site-specific accessibility

For un-methylated sites, we can estimate their accessibilities by:

$$\hat{\omega}_j^{\text{ivt}} = \frac{1}{(\frac{r_{jA}^{\text{ivt}}}{r_{jG}^{\text{ivt}}} + 1)\hat{k}_2}$$

We then fit a model between initial accessibility estimates and observed counts to further remove unwanted variation. Additionally, we introduce the following variables to present observed counts the IVT control:

- Total counts in IVT: N_j^{ivt}; log total counts in IVT: $n_j^{\text{ivt}} = \log_{10}(N_j^{\text{ivt}} + 1)$.

- Total G counts in IVT: M_j^{ivt}; log total counts in IVT: $m_j^{\text{ivt}} = \log_{10}(M_j^{\text{ivt}} + 1)$.
- Total A counts in IVT: L_j^{ivt}; log total counts in IVT: $l_j^{\text{ivt}} = \log_{10}(L_j^{\text{ivt}} + 1)$.

The following linear model (with intercept) was estimated using randomly sampled 2,000 data points with 10-fold cross-validation.

$$f(\omega^{\text{ivt}}; n^{\text{ivt}}, m^{\text{ivt}}, l^{\text{ivt}}) = \text{lm}\Big(\sqrt{\omega^{\text{ivt}}} \sim n^{\text{ivt}} + m^{\text{ivt}} + l^{\text{ivt}}\Big)$$

The accessibility for each site is predicted as,

$$\tilde{\omega}_i = f(\omega^{\text{ivt}}; n^{\text{ivt}}, m^{\text{ivt}}, l^{\text{ivt}} | n^{\text{ivt}} = n_i^{\text{ivt}}, m^{\text{ivt}} = m_i^{\text{ivt}}, l^{\text{ivt}} = l_i^{\text{ivt}})$$

5 Calibrating β estimates using site-specific accessibility to obtain methylation level

We calibrate the methylation level using $\hat{\theta}_i = \hat{\beta}_i / \tilde{\omega}_i$ where $\hat{\beta}_i$ is apparent methylation level obtained previously.

6 Calling methylated sites.

Final methylation sites are defined as the following:

- Total number of read counts greater than 10 in both IVT and untreated mRNA samples
- Fisher test between IVT and untreated mRNA with adjusted p-values no greater than 0.05
- At least 10% exposed methylation level

Transcriptome-Wide Profiling and Quantification of N6-Methyladenosine by Enzyme-Assisted Adenosine Deamination

Yulan Xiao, Shun Liu, Ruiqi Ge, Yuan Wu, Chuan He, Mengjie Chen and Weixin Tang

Supplementary Note 6

Model for an untreated mRNA sample with an FTO+ control

1 Model Annotations

In FTO- sample, m6A sites stay as m6A, un-methylated A will be converted into G. In FTO+ sample, m6A sites that react with FTO will be converted into G, m6A sites that do not react with FTO will stay methylation, un-methylated A will be converted into G. We assume the following global parameters:

- k_1: Conversion rate for FTO- sample
- k_2: Conversion rate for FTO+ sample
- γ: FTO efficiency in FTO+ sample

Look at each site i:

- θ_i: True A proportion, i.e., the underlying proportion of A in total A (including A and m6A) without any treatment
- ω_i: Site-specific accessibility.

Expected proportion of nucleotide A at each site i can be represented as:

$$R_{iA-} = 1 - k_1\omega_i\theta_i$$
$$R_{iA+} = 1 - k_2[\omega_i\theta_i + \omega_i\gamma(1 - \omega_i\theta_i)]$$

Expected proportion of nucleotide G at each site i can be represented as:

$$R_{iG-} = k_1\omega_i\theta_i$$
$$R_{iG+} = k_2[\omega_i\theta_i + \omega_i\gamma(1 - \omega_i\theta_i)]$$

Ideally, if $k_1 = k_2 = \alpha = \omega_i = 1$, $R_{iG-} = \theta_i$, $R_{iG+} = 1$, we can use $R_{iG+} - R_{iG-} = 1 - \theta_i$ to estimate the abundance of m6A. However, in reality, the conversion rates, FTO efficiency

and site accessibility are not all 100%, $R_{iG+} - R_{iG-} = k_2[\omega_i\theta_i + \omega_i\gamma(1-\omega_i\theta_i)] - k_1\omega_i\theta_i$, if $k_2 - k_1$ is very negative, we even observe reversed trend.

2 Estimating conversion rates

Majority of A sites are accessible and un-methylated.

For un-methylated A sites with full accessibility:

$$R_{iG-} = k_1$$
$$R_{iG+} = k_2$$

Let $\kappa = k_1/k_2$, $\delta = k_1 - k_2$, r_{iG-} and r_{iG+} represent observed G rates, respectively, for each site i, we will calculate:

$$\kappa_i = r_{iG-}/r_{iG+}$$
$$\delta_i = r_{iG-} - r_{iG+}$$

$\hat{\kappa} = \text{Median}(\kappa_i)$ and $\hat{\delta} = \text{Median}(\delta_i)$ where i belong to sites after removing 10% outliers. Finally, we estimate conversion rates by:

$$\hat{k_2} = \frac{\hat{\delta}}{\hat{\kappa} - 1}$$
$$\hat{k_1} = \hat{\kappa}\hat{k_2}$$

3 Estimating FTO efficiency from FTO+ sample.

Let us denote apparent methylation level or exposed methylation using $\beta_i = \omega_i\theta_i$. For highly methylated sites, where β_i is close to 0, rate of nucleotide G in FTO+ reduces to:

$$R_{iG+} = \alpha k_2$$

Assuming the sampling of G following a binomial model, we using the FTO- sample to quantify apparent methylation level $\hat{\beta}_i$ for each site. We select sites with an estimated methylation rate from FTO- greater than 0.95 and observed G rate in FTO+ greater than 0.25 (excluding low accessibility) to obtain an estimate of FTO efficiency, i.e, $\hat{\alpha}$.

4 Jointly estimating site-specific m6A methylation level θ_i and site-specific accessibility ω_i.

To begin, we introduce following variables to present observed counts the FTO- and FTO+:

- Total counts in FTO-: N_{i-}; Total G counts in FTO-: M_{i-};
- Total counts in FTO+: N_{i+}; Total G counts in FTO+: M_{i+}.

We assume reads for FTO- and FTO+ samples distribute according to the following Binomial distributions, respectively:

$$\begin{aligned}&\text{Binomial}\Big(N_{i-}, P_{i1} = \hat{k_1}\beta_i\Big)\\&\text{Binomial}\Big(N_{i+}, P_{i2} = \hat{k_2}[\beta_i + \hat{\alpha}\omega_i(1-\beta_i)]\Big)\end{aligned}$$

We can write the probability model for FTO- and FTO+ and its log likelihood as follows:

$$\begin{aligned}f(\theta_i,\omega_i) &= \binom{N_{i-}}{M_{i-}} P_{i1}^{M_{i-}}\big(1-P_{i1}\big)^{N_{i-}-M_{i-}} \binom{N_{i+}}{M_{i+}} P_{i2}^{M_{i+}}\big(1-P_{i2}\big)^{N_{i+}-M_{i+}}\\ \log f(\theta_i,\omega_i) &= M_{i-}\log\Big[\hat{k_1}\omega_i\theta_i\Big] + \Big(N_{i-}-M_{i-}\Big)\log\Big[1-\hat{k_1}\omega_i\theta_i\Big]\\ &+ M_{i+}\log\Big[\hat{k_2}\big[\omega_i\theta_i + \hat{\alpha}\ \omega_i(1-\omega_i\theta_i)\big]\Big] + \Big(N_{i+}-M_{i+}\Big)\log\Big[1-\hat{k_2}\big[\beta_i + \hat{\alpha}\ \omega_i(1-\omega_i\theta_i)\big]\Big]\end{aligned}$$

We then calculate MLE of θ_i and ω_i by optimizing the above log likelihood function using BFGS algorithm implemented in R function optim(), i.e., $(\hat{\theta}_i, \hat{\omega_i}) = \max_{(\theta_i,\omega_i)} \log f(\hat{\theta}_i, \hat{\omega_i})$.

5 Calling methylated sites.

Final methylation sites are defined as the following:

- Total number of read counts greater than 10 in both FTO+ and FTO- samples
- Fisher test between FTO+ and FTO- with adjusted p-values no greater than 0.05
- At least 10% exposed methylation level
